# Over Tenfold Increase in Current Amplification Due to Anisotropic Polymer Chain Alignment in Organic Electrochemical Transistors

**DOI:** 10.1002/adma.202420323

**Published:** 2025-06-02

**Authors:** Olivier Bardagot, Pablo Durand, Shubhradip Guchait, Han‐Yan Wu, Isabelle Heinzen, Wissal Errafi, Victor Bouylout, Alessandra Pistillo, Chi‐Yuan Yang, Gonzague Rebetez, Priscila Cavassin, Badr Jismy, Julien Réhault, Simone Fabiano, Martin Brinkmann, Nicolas Leclerc, Natalie Banerji

**Affiliations:** ^1^ Department of Chemistry Biochemistry and Pharmaceutical Sciences (DCBP) University of Bern Freiestrasse 3 Bern 3012 Switzerland; ^2^ Institute of Chemistry and Processes for Energy Environment and Health (ICPEES) University of Strasbourg CNRS, 25 rue Becquerel Strasbourg 67087 France; ^3^ Institut Charles Sadron (ICS) University of Strasbourg CNRS, 23 rue du Loess Strasbourg 67034 France; ^4^ Laboratory of Organic Electronics Department of Science and Technology Linköping University Norrköping 60174 Sweden

**Keywords:** anisotropic alignment, high‐temperature rubbing, organic electrochemical transistors, organic mixed ionic‐electronic conductors, pulsing stability, single‐ether side chains, THz spectroscopy

## Abstract

Organic electrochemical transistors (OECTs) are central to the development of highly sensitive (bio)sensors, energy‐efficient neuromorphic devices, and high‐precision electrophysiological monitoring systems. With growing interest in these strategic electronic devices, a novel PBTTT polymer bearing single‐ether side chains (**PBTTT‐^8^O**) in OECTs is investigated. Pristine isotropic non‐aligned OECT performance matches state‐of‐the‐art transconductance, highlighting the potential of single ethers for designing high‐performance organic mixed ionic‐electronic conductors (OMIECs). Moreover, a 13× enhancement of current output is achieved by anisotropic polymer chain alignment of **PBTTT‐^8^O**, opening doors to unprecedented device sensitivity. Compared to pristine ones, aligned OECTs afford a 6× increase in the normalized transconductance (g_m_L/Wd), reaching an unprecedented 2 580 S cm^−1^. Such improvement is mainly due to a gain in carrier mobility µ, as evidenced by four distinct methods. In addition, aligned OECTs exhibit faster doping front propagation, ON switching, and OFF switching compared to pristine ones. This study hence reports a versatile and easily transferable approach to concomitantly boost signal amplification and accelerate the response time of bioelectronic devices.

## Graphical abstract

How to boost the I_DS_ output of an OECT? Anisotropic polymer alignment along the channel direction enables a 13‐fold increase in I_DS_. Such channel engineering opens the door to unprecedented signal amplification (here g_m_L/Wd = 2 580 S cm^−1^ with a PBTTT‐backbone bearing single‐ether side chains), faster ON/OFF switching, and improved stability for the next‐generation bioelectronics.

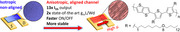

## 1 Introduction

Organic electrochemical transistors (OECTs) exhibit transconductance up to two orders of magnitude higher compared to organic field‐effect transistors (OFETs) thanks to the bulk electrochemical doping of an organic channel. OECTs are the cornerstone of emerging bioelectronic devices such as highly accurate biosensors,^[^ ^1^ ^]^ artificial neurons,^[^ ^2^ ^]^ and electrophysiological signal amplifiers.^[^ ^3^ ^]^ To boost their development, two key parameters must be improved: i) the OECT response time (e.g., to accurately monitor millisecond‐scale neural impulses^[^ ^2^ ^]^), and ii) the signal amplification (e.g., to maximize biosensing sensitivity^[^ ^1^ ^]^).

**Figure 1:**
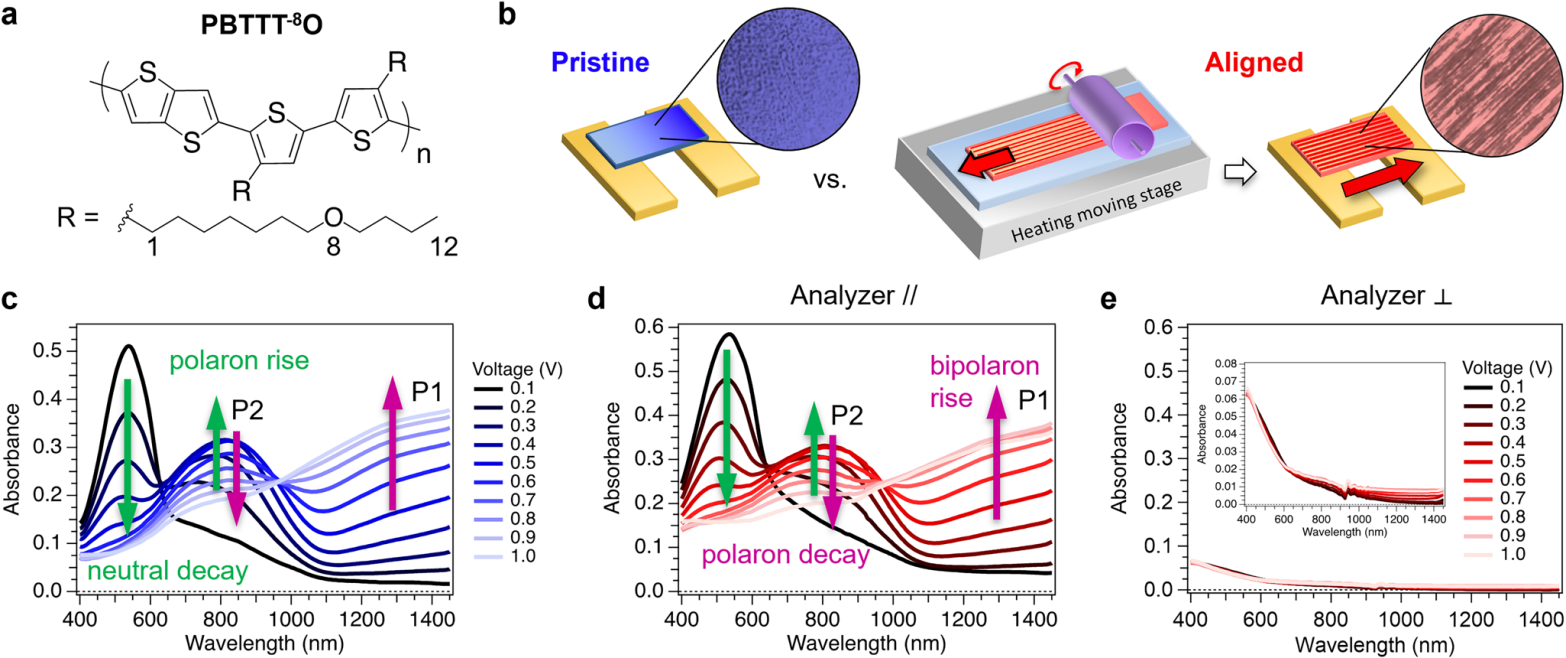
a) Chemical structure of PBTTT‐^8^O. b) Schematic representation of a (left, blue) pristine, non‐aligned, OECT and (right, red) the HTR method and a resulting aligned OECT after floating transfer (circles = polarized optical microscope photos). c–e) Steady‐state Vis‐NIR absorbance spectra upon electrochemical doping from +0.1 to +1.0 V in degassed 0.1 m KPF_6_/H_2_O (dwell time = 150 s) of c) pristine and d,e) aligned short‐circuited OECTs. For aligned OECTs, the light is polarized d) *parallel* to and e) *perpendicular* to the polymer chain alignment direction. Inset: zoom highlighting the negligible changes.

The latter depends on the transconductance (g_m_), which is proportional to the product of the charge carrier mobility and the volumetric capacitance (𝜇C*).^[^ ^4^ ^]^ From 2014 to 2020, the 𝜇C* product has been enhanced by three orders of magnitude, reaching ≈500 F cm^−1^ V^−1^ s^−1^.^[^ ^5^ ^]^ Currently, the main strategies to improve g_m_ explore: i) device engineering (e.g., electrode modification,^[^ ^6^ ^]^ vertical OECTs^[^ ^7^ ^]^), ii) conjugated backbone engineering (e.g., D‐A polymers,^[^ ^8^, ^9^ ^]^ regiochemistry^[^ ^10^, ^11^ ^]^) and iii) side‐chain engineering.^[^ ^5^, ^12^, ^13^, ^14^, ^15^, ^16^ ^]^ In particular, the substitution of alkyl side chains by polar, mostly oligo(ethylene glycol) (OEG), side chains affords higher ion uptake and ionic mobility due to higher polymer/ion affinity.^[^ ^12^, ^13^ ^]^ OECT properties can then be fine‐tuned to optimize both electronic and ionic transports by, for instance, alternating alkyl/polar side chains in copolymers,^[^ ^14^ ^]^ introducing alkyl spacers to move the polar segment away from the backbone,^[^ ^15^ ^]^ or varying the length of the OEG side chains.^[^ ^5^, ^16^, ^17^ ^]^ In 2022, this synthetic effort, combined with palladium scavenging and molar mass selection, led to a record 𝜇C* of ≈2 000 F cm^−1^ V^−1^ s^−1^ for P(g_4_2T‐TT) bearing OEG side chains (chemical structure in Table S1, Supporting Informaton).^[^ ^18^ ^]^ In 2024, a similar 𝜇C* and an enhanced geometry‐normalized peak transconductance (g_m_L/Wd) of ≈687 S cm^−1^ were achieved for PgBTTT bearing OEG side chains.^[^ ^11^ ^]^ Besides the strategies involving molecular engineering, it is possible to enhance charge transport in pristine and doped semiconductors by implementing polymer orientation methods in thin films such as strain‐alignment, epitaxy, or rubbing.^[^ ^19^, ^20^ ^]^ Uniaxial orientation indeed favors transport along the chain direction, which is more efficient than inter‐chain transport. To achieve high levels of orientation of polymers, the method of High‐Temperature Rubbing (HTR) is particularly effective. HTR is also versatile, as demonstrated for a large palette of polymers and different types of substrates.^[^ ^21^, ^22^ ^]^ HTR makes use of shearing forces at the interface between a microfiber‐coated rotating cylinder and a polymer film (**Figure** **1**). For polymers with alkyl segments in the side chains, the level of orientation (quantified by the dichroic ratio) is mostly driven by the temperature of the film during rubbing and by molecular parameters such as the molar mass distribution. Yet, the strategy of uniaxial polymer alignment has been marginally used to enhance OECT performance.

Here, we show the potential of PBTTT‐^8^O (Figure 1a)–a polythiophene decorated with single‐ether side chains–as a novel organic mixed‐ionic electronic conductor (OMIEC). Importantly, we also highlight the promises of anisotropic polymer alignment for OECT development by comparing the signal amplification, ON/OFF response time, and stability of PBTTT‐^8^O‐based OECTs made of (a) ‘pristine’ isotropic non‐aligned channels versus (b) ‘aligned’ anisotropic channels manufactured by HTR. The signal amplification is estimated through the absolute I_DS_ output and compared to literature via calculated g_m_L/Wd—independent of any model—and reported 𝜇C* based on the Bernards‐Malliaras model. The device response time is quantified by fitting the exponential decay/rise of the polymer neutral absorbance band and I_DS_ output over 1000 ON/OFF V_GS_ pulses using time‐resolved *in operando* spectroelectrochemistry. We evidence that PBTTT‐^8^O alignment is an effective strategy to a) enhance both g_m_L/Wd and 𝜇C* by one order of magnitude, and b) speed up the doping (ON) and dedoping (OFF) kinetics of the channel by factors of ≈2× and 4×, respectively. Pulsing stability is also enhanced, regardless of the bias stress applied to the channel. Our results suggest that combining ongoing molecular engineering and highly anisotropic films will advance bioelectronics and other asymmetric electrochemical devices.

## 2 Results and Discussion

### 2.1 Single‐Ether Side Chains and Polymer Alignment

All OECTs studied here are composed of **PBTTT‐^8^O** as channel material (Mw = 54.1 kg mol^−1^, Ɖ = 1.8, HOMO ≈ −4.8 eV^[^ ^23^ ^]^). **PBTTT‐^8^O** bears single‐ether side chains containing only one oxygen atom in the 8^th^ position of the alkyl side chain (Figure 1a).^[^ ^23^ ^]^ Similarly to PgBTTT,^[^ ^10^, ^11^ ^]^ the side chains of **PBTTT‐^8^O** are grafted on the 4,4’‐positions, enhancing the backbone planarity and the hole mobility in OECTs compared to the 3‐3’ 3,3'‐positions of the widely‐studied regioisomer P(g_3_2T‐TT) (structures in Figure S1, Supporting Information).^[^ ^10^ ^]^ The single‐ether side chains are designed for balanced polarity and polymer crystallinity, in contrast to vastly used, yet more disordered, OEG side chains.^[^ ^23^, ^24^ ^]^ For pristine OECTs, the polymer is spin‐coated without post‐treatment (Figure 1b). For aligned OECTs, the polymer is first doctor‐bladed and then aligned by high‐temperature rubbing (HTR) at 170 °C.^[^ ^21^ ^]^ Images of the resulting films are reported in Figures S2–S5 (Supporting Information). By selecting 170 °C as rubbing temperature (T_R_), Durand et al. demonstrated metal‐like transport with conductivities over 10^4^ S cm^−1^ along the chain alignment direction for chemically doped films of **PBTTT‐^8^O**.^[^ ^23^ ^]^ The HTR method affords large dichroic ratios between 7 and 12 for **PBTTT‐^8^O** (Figure S6, Supporting Information). In comparison, dichroic ratios of ≈4 were achieved for P(g2T‐T) upon 200% film stretching (Figure S1, Supporting Information).^[^ ^25^ ^]^ Aligned channels are rough (Figures S2–S4, Supporting Information), so an average thickness (d) is estimated using a calibration curve made from dry pristine films (Figure S6 and Note S2, Supporting Information) and applied to the isotropic absorbance of aligned films reconstructed from polarized absorbance spectra (Figure S7, Supporting Information). The estimated average thickness is confirmed by melt‐annealing at 350 °C to smooth the films (Figure S8, Supporting Information).^[^ ^35^ ^]^

### 2.2 Optical Response

In 0.1 mol L^−1^ aqueous KPF_6_ electrolyte at +0.1 V, the steady‐state absorbance spectra of pristine and aligned samples both exhibit the S_0_‐S_1_ transition as a Gaussian‐like band centered at ≈540 nm with a shoulder at ≈580 nm (neutral state, Figure 1c,d).^[^ ^26^ ^]^ Compared to the dry films (Figure S7, Supporting Information), the injection of PF_6_^−^ anions into the polymer matrix under bias, from +0.1 to +0.5 V, induces the decay of the neutral band and the rise of broad bands centered at ≈800 and >1400 nm, typically assigned to the P2 and P1 bands of polarons, respectively.^[^ ^23^, ^28^ ^]^ At voltages higher than +0.5 V, the P2 peak decays in favor of a more intense absorbance above 1200 nm, ascribed to the conversion of polarons to higher oxidized species (e.g., bipolarons). The broad absorbance observed at +1.0 V with complete disappearance of the neutral band indicates that the whole depth of the film is doped, reaching a high doping level. The absorbance spectra of aligned films probed with light polarized *parallel* to the rubbing direction as a function of the doping voltage exhibit no significant difference from the pristine ones (Figure 1c,d; Figure S9, Supporting Information). Conversely, if the light is polarized *perpendicular* to the rubbing direction, the resulting spectra are essentially independent of the voltage doping and show very low absorbance (Figure 1e). This highlights that few polymer backbones are oriented *perpendicular* to the rubbing grooves and that the high dichroic ratio is preserved despite ion uptake and film swelling during doping. We can hence speculate that the PF_6_^−^ anions are mainly injected in the side chains regions without observable damage of the backbone order, similarly to PF_6_^−^ into pristine PBTTT‐C_14_ films^[^ ^29^ ^]^ and to molecular dopants into aligned **PBTTT‐^8^O** films.^[^ ^23^ ^]^

### 2.3 Signal Amplification

**Figure 2:**
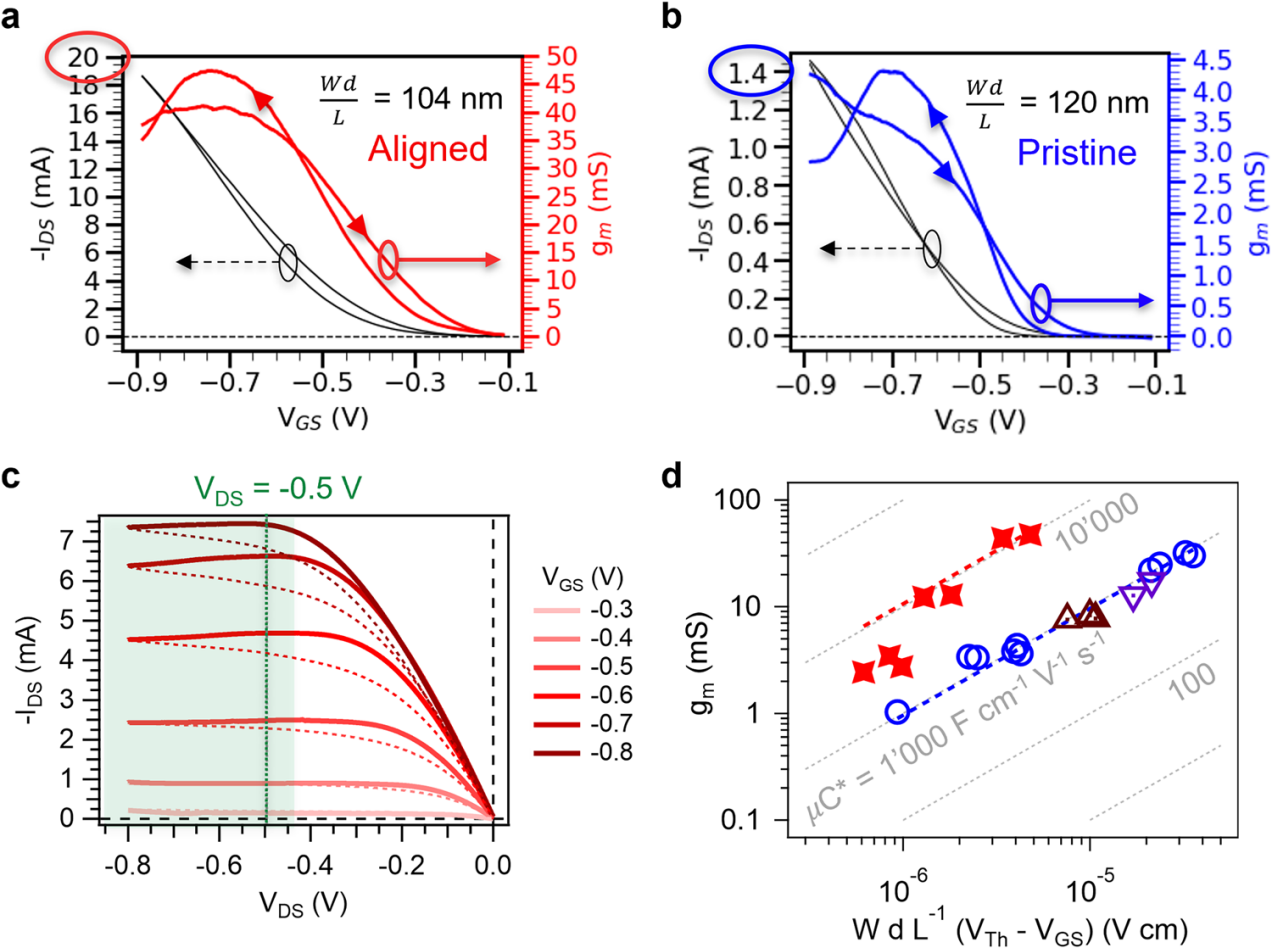
a,b) Transfer characteristics of a) aligned vs. b) pristine PBTTT‐^8^O OECTs (V_DS_ = −0.5 V, scan rate = 2 mV s^−1^). The left y‐axis shows the resulting I_DS_ current, while the colored right y‐axis indicates the corresponding transconductance g_m_ calculated from the derivative of I_DS_ over V_GS_. c) Output characteristics of an aligned OECT, highlighting that the transistor operates in the saturation regime (green region) for V_DS_ = −0.5 V. d) Linear dependence of g_m_ as a function of the channel dimensions and voltage parameters (red stars: doctor‐bladed and HTR‐aligned at 170 °C, blue circles: spin‐coated pristine (non‐aligned, non‐annealed), brown upward triangles: doctor‐bladed but non‐aligned, purple downward triangles: spin‐coated non‐aligned but annealed at 170 °C). The dotted grey lines indicate constant 𝜇C* products as guides to the eye. The channel dimensions, V_Th_, V_GS_ at maximum g_m_, g_m_, and I_ON_/I_OFF_ ratio are detailed for each device in Table S2 (Supporting Information).

Notably, a drain‐source current (I_DS_) current of −19 mA is obtained for a V_GS_ of only −0.9 V for an aligned **PBTTT‐^8^O** OECT (**Figure** **2**a). For direct comparison, a maximum I_DS_ of −1.5 mA is achieved for a pristine **PBTTT‐^8^O** OECT of similar channel dimensions (Figure 2b). A 13× enhancement in I_DS_ output is thus observed directly from the data. Moreover, an absolute value of g_m_ as high as 47 mS is achieved for an 80 nm‐thick aligned channel at a bias voltage lower than ‐0.75 V, making these devices primary candidates for the manufacture of high‐sensitivity (bio)sensors with low‐power consumption (Table S2, Supporting Information). When normalized by the channel geometric factor (Wd/L), the output characteristics also indicate a ≈14× higher I_DS_ for aligned OECTs (Figure 2c) compared to pristine ones (Figure S10, Supporting Information). A drain‐source voltage (V_DS_) of −0.5 V is applied for all transfer characteristics to operate in the saturation regime. All performance metrics are extracted from the forward sweep (Figures S11–S18, Supporting Information). These observations translate into a 6× increase in g_m_L/Wd from 430 ± 70 S cm^−1^ to 2 580 ± 1 220 S cm^−1^, thereby demonstrating the net gain of aligning the polymer chains of **PBTTT‐^8^O** parallel to the channel direction (**Table** **1**, details in Table S2, Supporting Information). Such normalized peak transconductance is among the highest reported to date (Figure 4a), as highlighted by comparison with the g_m_L/Wd values calculated for the literature and listed in Table S1 (Supporting Information). We propose to use g_m_L/Wd as a complementary figure‐of‐merit for OECT benchmarking, as it is independent of any model, conversely to µC*. The effects of doctor‐blading and post‐deposition thermal annealing at T_R_ = 170 °C are negligible on the OECT performance of the non‐aligned polymer (Figures S15 and S16, Supporting Information), allowing us to unequivocally attribute the improved performance to the high anisotropy of the aligned channels (dichroic ratio > 7). Note that a large channel length (L) of 2 mm is used to allow the spectroelectrochemical characterization, which affects the performance metrics. For 10 µm‐long channels, technical challenges with delamination occurred (Note S1 and Figure S5, Supporting Information), but it has been shown that despite higher contact resistance, decreasing the channel length theoretically reduces the total OECT resistances and leads to an even greater increase in g_m_.^[^ ^30^ ^]^ To further compare the results to the current literature, we estimate the *µ*C* product using the Bernards‐Malliaras model, assuming a one‐to‐one conversion of ions to charges. Although debatable, extracting 𝜇C* from this model remains the most common method to quantify the performance of OECTs to date, and enables direct comparisons with the pertinent literature.^[^ ^11^, ^31^, ^32^, ^33^, ^34^ ^]^ One should, however, keep in mind that the *µ*C* figure of merit encompasses both the OMIEC material properties and the OECT device properties.^[^ ^31^ ^]^ In this model, the channel is approximated as a “volumetric capacitor,” and the transconductance of OECTs with different channel dimensions scales linearly with the geometric and bias factors, giving 𝜇C* as the slope of Figure 2d (Equation, (S2) and Figure S12, Supporting Information).^[^ ^4^ ^]^ A state‐of‐the‐art 𝜇C* of 960 ± 30 F cm^−1^ V^−1^ s^−1^ is found for pristine **PBTTT‐^8^O**‐based OECTs in aqueous KPF_6_ electrolyte. Comparison to other p‐type OECTs underlines that, even in its pristine isotropic state, **PBTTT‐^8^O** OECTs rival the best‐performing polymers reported in literature, thereby demonstrating the potential of single‐ether side chains for designing efficient OMIECs. The alignment of **PBTTT‐^8^O** chains along the channel direction by HTR leads to a further 11× enhancement of 𝜇C* (Figure 2d), reaching an unprecedented 10 660 ± 910 F cm^−1^ V^−1^ s^−1^ (Figure 4b). The general benefit of uniaxial polymer alignment is supported by a recent report from Flagg et al. of a 8× improvement in *µ*C* upon anisotropic crystallization of epitaxially‐oriented P3HT along the channel direction.^[^ ^33^ ^]^ However, epitaxial orientation is a rather slow process, difficult to upscale, and requiring chlorinated molecules such as 1,3,5‐trichlorobenzene, as opposed to the more versatile HTR method.

**Table 1:** OECT performance of **PBTTT‐^8^O** (7–10 devices) compared with the highest reported p‐type OMIECs.

Materials	g_m_L/Wd [S cm^−1^]^a)^	µC^*^ [F cm^−1^ V^−1^ s^−1^]^e)^	C^*^ [F cm^−3^]	µ [cm^2^ V^−1^ s^−1^]	V_Th_ [V]^l)^	Refs.
Aligned PBTTT‐^8^O	2 580 ± 1 220^b)^, ^c)^	10 660 ± 910	560 ± 70^f)^	19 ± 4.0^h)^ 15 ± 9.8^i)^ 2.0^j)^ 6.5^k)^	−0.32 ± 0.04	This work
Pristine PBTTT‐^8^O	430 ± 70^b)^, ^c)^	960 ± 30	390 ± 40^f)^	2.5 ± 0.3^h)^ 3.3 ± 0.8^i)^ 0.11^j)^ 3.7^k)^	−0.32 ± 0.06	This work
Pg_3_BTTT	687 ± 91^c)^	1 983 ± 169	/	/	−0.25 ± 0.02	[11]
Pg_3_BTTT	194 ± 2^d)^	502 ± 18	164 ± 7^g)^	3.44 ± 0.13^i)^	−0.24	[10]
P(g**_4_** 2T‐TT) Mn‐selected	146 ± 0.6^d)^	2008 ± 130	308 ± 20^g)^	6.53 ± 0.07^h)^	0.07 ± 0.003	[18]
P(g_3_2T‐T)	130 ± 6.8	556 ± 44	166^g)^	3.35 ± 0.26^i)^	−0.06	[44]
P(g_3_2T‐TT)	135^d)^	227 ± 107	241 ± 94^g)^	0.94 ± 0.25^j)^	0.00	[50]

^a)^ Average geometry‐normalized peak transconductance (g_m_L/Wd), highlighting the remarkable value, independent of any model, achieved compared to the literature;

^b)^ OECTs operating in 0.1 M KPF_6_/H_2_O electrolyte and not NaCl/H_2_O electrolyte;

^c)^ OECTs with mm‐long L and W.

^d)^ Calculated from gm ± Δgm, d ± Δd, L, and W reported in the text of the references.

^e)^ *µ*C* figure‐of‐merit extracted from the slope of Figure 2c, under the assumptions of the Bernards–Malliaras model, for comparison to existing literature;

^f)^ C* estimated by chronoamperometry;

^g)^ C* estimated by electrochemical impedance spectroscopy (EIS);

^h)^ 𝜇 inferred from 𝜇C*/C*;

^i)^ 𝜇 inferred from the slope of √I_DS_^Sat^ vs. V_GS_ plot in the saturation regime and C*;

^j)^ 𝜇 directly measured by impedance matching;

^k)^ 𝜇 directly measured by THz spectroscopy;

^l)^ Average V_Th_ defined as the intercept of the linear regression of √I_DS_^Sat^ vs. V_GS_ plot and the abscissa (Figure S13). The uncertainty given is one standard deviation;

Similar average V_Th_ of −0.33 ± 0.04 V and −0.32 ± 0.06 V are found for both aligned and pristine OECTs, respectively (Figure S13 and Table S2, Supporting Information). Conversely, the use of the single‐ether side chains decreases V_Th_ by −0.09 V (−0.33 V for **PBTTT‐^8^O**) compared to the same polymer backbone bearing OEG side chains (−0.24 V^[^ ^10^ ^]^/−0.25 V^[^ ^11^ ^]^ for PgBTTT). This may be attributed to a deepening of the HOMO level by removing the electron donating alkoxy function.^[^ ^15^ ^]^ Decreasing V_Th_ is a strategy to mitigate oxygen‐mediated degradation,^[^ ^27^ ^]^ and thus may be a path to increase the stability of OECTs. Finally, both pristine and aligned OECTs exhibit large I_ON_/I_OFF_ ratios superior to 10^4^ (Figure S14, Supporting Information).^[^ ^36^ ^]^

To rationalize the origin of the high electrical performance of pristine and aligned OECTs, we estimate the volumetric capacitance C* (Note S5, Supporting Information).^[^ ^37^ ^]^ A 1.4× higher average C* is found for aligned samples (560 ± 70 F cm^−3^) compared to pristine ones (390 ± 40 F cm^−3^) from the slope of the injected carrier density as a function of voltages above V_Th_ using chronoamperometry (Figure S18, Supporting Information, film dimensions in Table S4, Supporting Information). The carrier density probed by chronoamperometry includes the current from both the “nonvolumetric capacitance” (circuit charging, double layer formation) and the ‘volumetric capacitance’ (bulk doping of the polymer), leading to a likely overestimation of C* compared to electrochemical impedance spectroscopy (EIS).^[^ ^16^ ^]^ Unfortunately, the response frequency of PBTTT‐^8^O is too low, rendering C* estimation by EIS not reliable (Figure S19, Supporting Information). Nevertheless, chronoamperometry allows a fair‐comparison between the aligned and nonaligned OECT channels, and the C* values found are on the same order of magnitude as the ones reported in literature by EIS (e.g. C* = 308 F cm^−3^ for P(g_4_2T‐TT),^[^ ^18^ ^]^ Table S1, Supporting Information). Interestingly, the 1.4× increase in C* does not explain the 6× and 11× enhancements in g_m_L/Wd and 𝜇C*, respectively, observed between pristine and aligned OECTs. This suggests that capacitive interfacial effects arising from the rougher surface of aligned films are only a marginal improvement factor. Instead, it is the increase of 𝜇, rather than of C*, that defines their superior performance.

We estimate the hole mobility in the swollen state (𝜇) using four different methods (Note S6, Supporting Information). First, under the strong assumptions of the widely‐used Bernard–Malliaras model (zero contact resistance, channel resistance reciprocal to the gate voltage),^[^ ^31^, ^38^ ^]^ we infer 𝜇 i) by dividing 𝜇C* by C*,^[^ ^9^, ^16^, ^17^, ^18^, ^24^, ^39^, ^40^, ^41^, ^42^ ^]^ and ii) from the slope of the √I_DS_^Sat^ vs. V_GS_ plot and C*.^[^ ^5^, ^8^, ^10^, ^27^, ^43^, ^44^, ^45^, ^46^, ^47^ ^]^ We find an average value of 19 ± 4.0 cm^2^ V^−1^ s^−1^ with the µC*/C* method for the aligned **PBTTT‐^8^O** OECT (Figure 4c), which represents a 7.6× improvement over the non‐aligned device and largely outperforms the current record µC*/C* of 6.5 cm^2^ V^−1^ s^−1^ reported for P(g_4_2T‐TT).^[^ ^18^ ^]^ Using the slope method, the mobility extracted for pristine **PBTTT‐^8^O** is similar to the one of PgBTTT (3.3 ± 0.8 cm^2^ V^−1^ s^−1^ vs 3.4 cm^2^ V^−1^ s^−1^, Table 1),^[^ ^10^ ^]^ and a 4.5× enhancement occurs upon alignment. Second, we use two direct methods – independent of C* – to confirm the enhancement of i) the long‐range mobility (mm‐scale) by impedance matching^[^ ^4^, ^37^, ^48^, ^49^, ^50^ ^]^ and ii) the short‐range mobility (nm‐scale) by in situ electrochemical THz spectroscopy.^[^ ^51^ ^]^ Again, an important 17× enhancement in *µ* is found between aligned and pristine OECTs by impedance matching (Figures S20–S24, Supporting Information). The mobility measured for aligned OECTs is higher than any value reported to date using this method (Table S1, Supporting Information) but is “only” of 2.0 cm^2^ V^−1^ s^−1^. In comparison, an effective short‐range mobility of 6.5 cm^2^ V^−1^ s^−1^ is extracted from the complex THz conductivity (Figures S25 and S26 and Table S5, Supporting Information). A larger mobility at short range is expected, as long‐range disorder, grain‐boundary effects, and interfacial resistances are excluded. We find a 1.8× enhancement of the short‐range *µ* upon chain alignment, which is less pronounced than on the long range (17×). The overall gain in mobility is attributed to enhanced intra‐chain carrier delocalization^[^ ^52^ ^]^ and inter‐chain hopping along π‐stacking sites,^[^ ^53^, ^54^ ^]^ due to a higher molecular order at a nm‐scale (probed by THz) and a favorable orientation of the ordered regions in the channel direction at a mm‐scale (probed by impedance matching), likely offering lower contact resistances.^[^ ^6^ ^]^ The discrepancy between *µ* from direct and indirect methods reflects: i) the importance of comparing *µ* extracted from the same method, ii) the uncertainty on C* and iii) the limitations of the assumptions made in the Bernards‐Malliaras model.^[^ ^38^ ^]^ This comparison encourages to think that mobility values reported from indirect methods are overestimated. Besides, the slope of Figure 2d may equal a term more complex than the product of independent *µ* and C* and the “one‐to‐one conversion” assumption may not strictly hold, resulting in a potential overestimation of *µ*C* (Figure S12, Supporting Information).^[^ ^34^ ^]^ Nonetheless, the gain in *µ*, as well as the over tenfold amplification of the I_DS_ current and record normalized transconductance g_m_L/Wd achieved with chain alignment, are unequivocal.

### 2.4 Response Time

**Figure 3:**
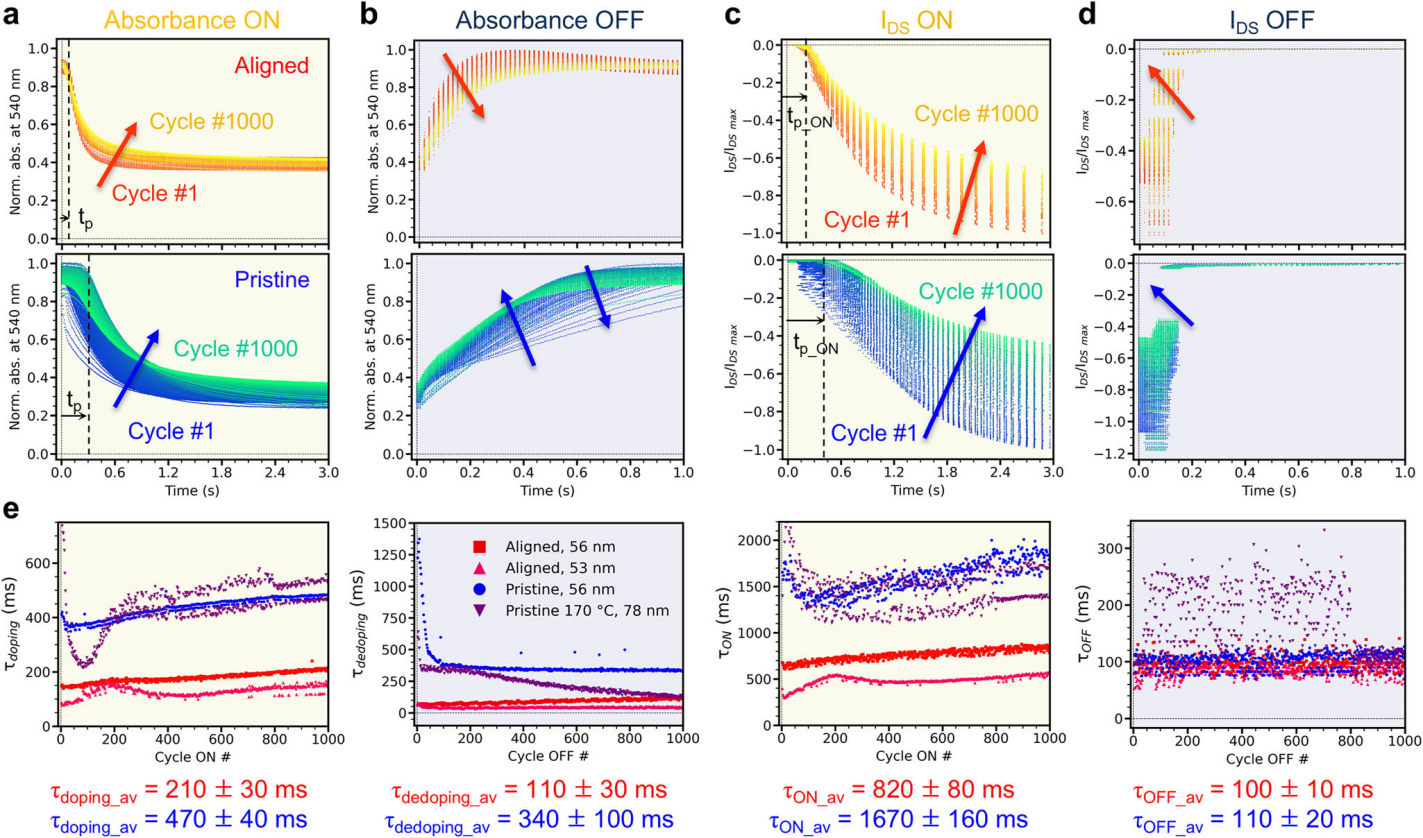
Evolution of a,b) the normalized absorbance at 540 nm (∼neutral band, wavelength sampling) and c,d) resulting I_DS_ current upon a–c) 1000 ON and b–d) 1000 OFF switching from −0.8 to +0.5 V (full range, dwell ON/OFF = 3/1 s, stress duration = 4000 s). (top) Aligned and (bottom) pristine PBTTT‐^8^O OECTs (both, d = 56 nm). e) Cycle‐after‐cycle evolution of the optical doping (τ_doping_) and dedoping (τ_dedoping_), and I_DS_ ON (τ_ON_) and OFF (τ_OFF_) time constants for aligned (red and pink), pristine (blue), and pristine but annealed (purple) OECTs.

In addition to the high transconductance and low threshold voltage of OECTs, the broader application of such devices requires rapid ON/OFF switching speeds and long‐term operational stability. As gating an OECT with rapid V_GS_ pulses is a practical operating mode for biosensing^[^ ^1^, ^55^ ^]^ and neuromorphics,^[^ ^2^ ^]^ we analyze the doping (ON) and dedoping (OFF) kinetics of **PBTTT‐^8^O**‐based OECTs over 1000 ON/OFF pulsing cycles. We monitor the transient evolution of the Vis‐NIR absorbance spectra of the channel and resulting I_DS_ current by *in‐operando* spectroelectrochemistry (**Figure** **3**). Normalized absorbance and I_DS_ data of each of the successive 1000 cycles are fitted using a mono‐exponential function (Figures S27 and S28 and Note S7, Supporting Information). Upon doping by switching from +0.5 to −0.8 V, the decay of the neutral polymer absorbance at 540 nm occurs with an average optical time constant (τ_doping_) of 210 ± 30 ms for the aligned OECTs. This is 2.2× faster than for pristine films with an identical channel thickness of 56 nm (470 ± 30 ms, Figure 3a). It translates into a 2.0× faster I_DS_ ON‐switching time (τ_ON_ of 820 ± 80 ms and 1670 ± 160 ms, respectively, Figure 3c). The faster OECT response is also manifested by a shorter doping front propagation time (t_p_^[^ ^56^ ^]^), fully consistent with the higher *µ* found for aligned channels (Figure S29, Supporting Information). When dedoping by switching from −0.8 to +0.5 V, the rise of the neutral band occurs with an average optical time constant (τ_dedoping_) of 110 ± 30 ms for the aligned OECT, which is 3.0× faster than for the pristine one (340 ± 100 ms, Figure 3b). The OFF‐switching of I_DS_ falls within the limit of the acquisition speed of our setup for both OECTs (τ_OFF_ ≈ 100 ms, Figure 3d) but a 4.8× faster OFF‐switching is confirmed for an aligned OECT probed with a higher time resolution (τ_OFF_ ≈ 23 ms vs 110 ms for the pristine one, Figure S30, Supporting Information). Note that faster τ_OFF_ (τ_dedoping_) compared to τ_ON_ (τ_doping_) is attributed to carrier‐density‐dependent mobility and faster dedoping than doping kinetics of the polymer, as shown by Guo et al.^[^ ^56^ ^]^ (Figure 3e). Faster time constants could be achieved by decreasing the width and length of the aligned channels.^[^ ^57^ ^]^

**Figure 4:**
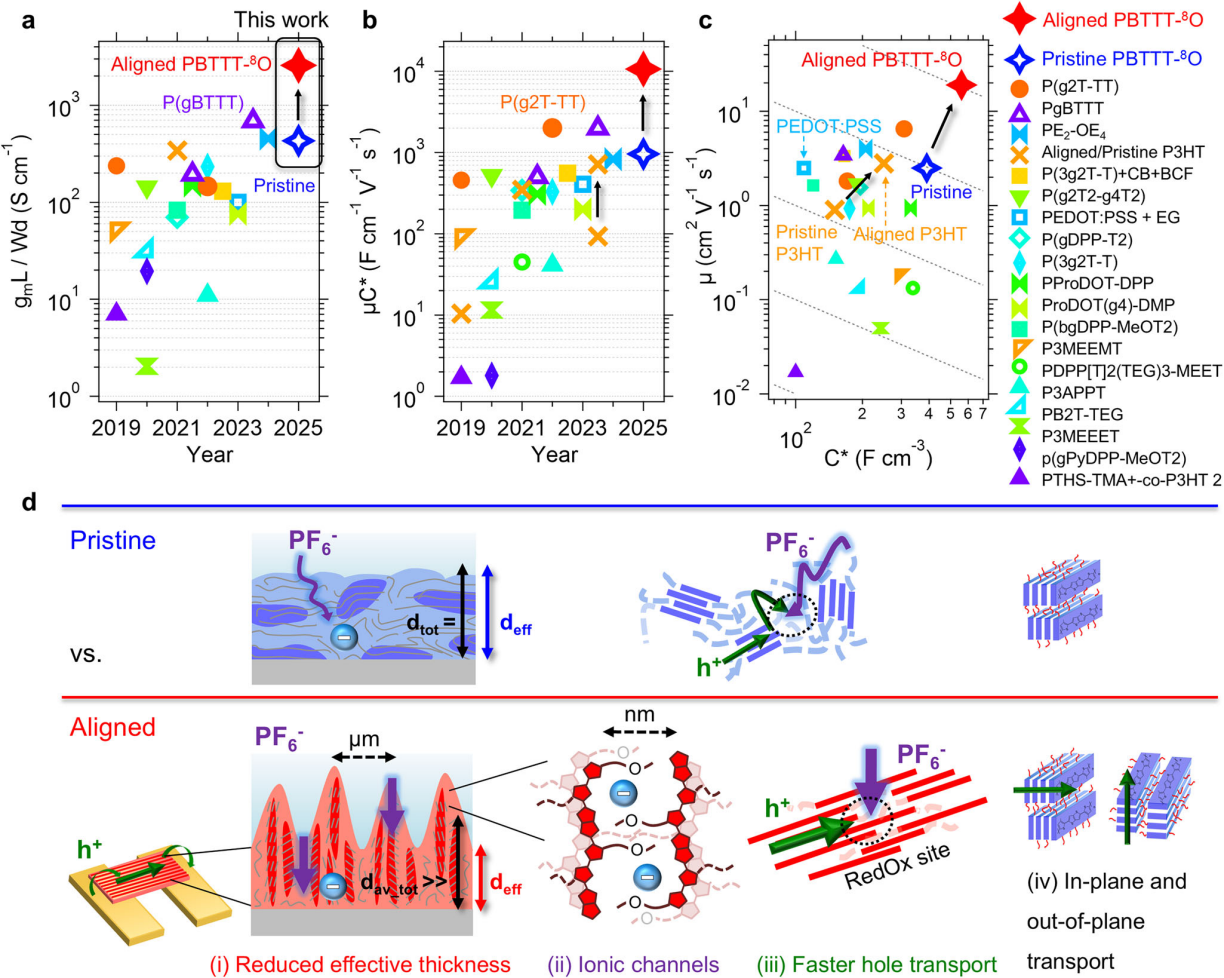
a) Progress in g_m_W/dL and b) 𝜇C* made over the years for p‐type OECTs reported in the literature (details in Table S1, Supporting Information). This graph underlines the high performance of pristine PBTTT‐^8^O (blue open star), and the sixfold g_m_W/dL enhancement achieved by high‐temperature rubbing (red star). c) Comparison with the literature of C* and inferred hole mobility 𝜇 from indirect methods. a,b) The black arrows highlight the similar gain in *µ*C*, C*, and *µ* thanks to anisotropic alignment reported in this work and for P3HT by Flagg et al.^[^ ^33^ ^]^ d) Schematic representation of the main parameters thought to allow higher signal amplification and faster ON/OFF response.

Hypothetical reasons for the faster dedoping, doping, and front propagation times found for aligned OECTs are manyfold. i) Ion injection/expulsion may be favored by the formation of “hills” and “grooves” at the µm‐scale caused by mechanical HTR, increasing the surface/volume ratio of the polymer channel and reducing the ‘effective thickness’ that the ions must penetrate (Figures S3 and S4, Supporting Information). Such an effect was recently observed for porous channels, where the increase of interfacial area allowed accelerated ON switching of OECTs.^[^ ^58^, ^59^ ^]^ ii) After alignment, the large fraction of well‐ordered yet soft regions formed by the side chains may act as ‘ionic channels’ to facilitate hydrated ion insertion within the bulk of the film,^[^ ^60^ ^]^ without hindering the long‐range hole transport properties in the neighboring aligned polymer chains.^[^ ^40^ ^]^ This is supported by previous studies showing that molecular dopants intercalate in the side chain layers of **PBTTT‐^8^O**.^[^ ^61^, ^62^ ^]^ iii) The enhanced in‐plane hole mobility (*µ_aligned_* > *µ_pristine_*) allows faster injection of charge carriers and hopping across the channel to the oxidation sites near the ions.^[^ ^63^ ^]^ iv) Moreover, the out‐of‐plane hole mobility through the channel thickness might be improved, allowing faster (de)doping of the entire film volume, since aligned **PBTTT‐^8^O** films exhibit both edge‐on and face‐on organization when rubbed,^[^ ^61^ ^]^ while pristine films are predominantly edge‐on (as shown by electron diffraction, Figure S32, Supporting Information).^[^ ^20^ ^]^ v) A lower activation barrier for carrier formation in the more rigid aligned chains with higher order is expected, since carriers are entropically favored on planar backbones.^[^ ^64^ ^]^ These hypotheses would also explain why both µ and C* are enhanced concurrently upon high‐temperature rubbing. **Figure** **4**d is a simplified visualization of these hypotheses.

### 2.5 Operational Stability

**Figure 5:**
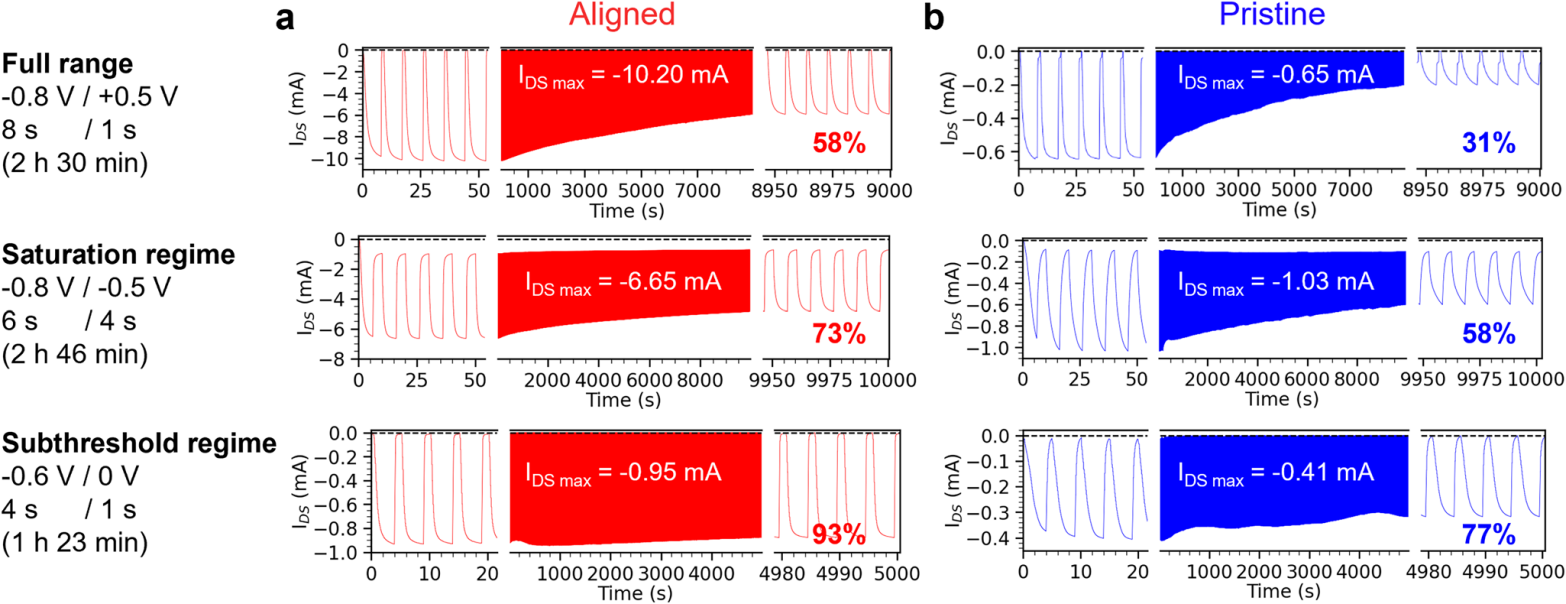
Pulsing stability over 1000 cycles of a) aligned and b) pristine PBTTT‐^8^O OECTs in (from top to bottom) the full operating range, the saturation regime and the subthreshold regime (Ti/Au SD electrodes, V_DS_ = ‐0.5 V, d = 45–57 nm, W = 2.7–3.1 mm, L = 2.0 mm, $\frac{Wd}{L}$ = 68–81 nm). Inset: I_DS_ retention after 1000 ON/OFF cycles normalized by the maximum I_DS_ reached for all cycles.

In addition, the pulsing experiment allows us to access the operational stability of OECTs in “real‐use” conditions. In these experiments, the gold‐undercoating chromium adhesion layer is replaced by titanium to rule out potential oxidation of Cr(0) and dissolution of hydrated Cr oxide over cycling.^[^ ^65^ ^]^ Three regimes of ON/OFF voltages were tested: the full operating range (from +0.5 to −0.8 V), the saturation regime (from −0.5 to −0.8 V), and the subthreshold regime (from 0 to −0.6 V). We observe a clear increase of the retention of I_DS_ from 58% to 73% to 93% for aligned OECTs for these regimes, respectively, after 1000 ON and 1000 OFF cycles (**Figure** **5**; Figure S33, Supporting Information). An identical trend, but with lower I_DS_ retention, is observed for pristine OECTs (31%, 58%, 77% for each regime, respectively). Polymer alignment by high‐temperature rubbing, hence, enhances the operational stability of OECTs on top of increasing the signal amplification. The main cause of I_DS_ loss is not the degradation of the polymer (only 12% loss after 1000 cycles, as quantified by cyclic voltammetry (CV), Figure S34b and Note S8, Supporting Information), but rather assigned to an increase of the contact resistance over repeated swelling/deswelling of the channel.^[^ ^66^ ^]^ The lower doping extent (Figure 3a), slower τ_ON_ (Figure 3e) and shift to higher potentials of the oxidation peak in CV (Figure S34c, Supporting Information) cycle after cycle indeed points toward a decrease of the effective voltage felt by the channel at a constant applied V_GS_, resulting in a lower I_DS_.^[^ ^17^ ^]^ This deduction is supported by the gain in stability when operating the OECT in the subthreshold regime, in accordance with the results of Keene et al.^[^ ^67^ ^]^ Running five consecutive transfer characteristics supports that the device stability, here 76% g_m_‐retention for aligned versus 68% for pristine, is dominated by the total bias‐stress duration and the doping extent reached, rather than the number of ON/OFF cycles (Figure S35 and Table S7, Supporting Information). Operating aligned OECTs in the subthreshold regime is hence recommended for applications requiring long‐term stability, such as electrophysiological monitoring at low power consumption.^[^ ^68^ ^]^

## 3 Conclusion

In this work, we study the impact of high‐temperature rubbing on the signal amplification, ON/OFF response time, and stability of OECTs made of **PBTTT‐^8^O** bearing single‐ether side chains in aqueous KPF_6_ electrolyte. First, our results show that state‐of‐the‐art performance can be achieved with single‐ether side chains, whose balanced properties in terms of crystallinity (better order) and polarity (ion uptake),^[^ ^62^ ^]^ make it a promising alternative to all‐glycol side chains for the design of efficient OMIECs operating in aqueous electrolytes. Second, our results unambiguously reveal the potential of polymer alignment along the OECT channel direction to induce an over tenfold amplification of the I_DS_ output signal compared to conventional pristine non‐aligned channels, affording high normalized peak transconductance g_m_L/Wd (over 2500 S cm^−1^), high 𝜇C* (over 10 000 F cm^−1^ V^−1^ s^−1^ under the assumptions of the Bernards‐Malliaras model), as well as faster ON and OFF response times and improved operational stability. These benefits are dominantly attributed to enhanced hole mobility, as consistently estimated for four independent methods. In particular, the short‐range conductivity can be better maintained at the long range when the polymer chains are aligned. Anisotropic polymer alignment, hence, appears to be an efficient strategy to address the challenge of accommodating solvated ions within the polymer matrix without disrupting the long‐range electronic pathways. High‐temperature rubbing is a particularly appealing method for aligning polymers as it can be implemented on a large library of materials, both p‐type and n‐type, and is compatible with roll‐to‐roll printing for large‐scale device production. Despite being applicable to different OECT architectures (planar, vertical, porous), the optimization of highly anisotropic polymer channels remains largely unexplored. Channel engineering is foreseen to unlock technological bottlenecks to foster the progress of a vast range of health and energy applications relying on electrochemical doping of semiconducting polymers.

## 4 Experimental Section

### Materials

Ultra‐flat quartz coated glass (S151) was purchased from Ossila. Potassium hexafluorophosphate (KPF_6_, 99.5%) and o‐dichlorobenzene (anhydrous, 99.8%) were purchased from Sigma‐Aldrich. The Ag/AgCl gate electrode (E205 pellet 1.0 × 2.5 mm (D x L)) was purchased from Warner Instruments. All chemicals in this study were used as received. The synthesis of **PBTTT‐^8^O** (Mw = 54.1 kg mol^−1^, Ɖ = 1.8) is detailed in the literature.^[^ ^23^ ^]^

### OECT Fabrication

Patterned Cr/Au (10/60 nm) drain‐source (DS) electrodes were thermally evaporated on S151 slides to form pre‐patterned OECT substrates. The substrates were sequentially sonicated in Hellmanex (1% by vol.), bi‐distilled water, acetone, and isopropyl alcohol (10 min each, room temperature (RT)) and UV‐O_3_ treated (30 min, RT). **PBTTT‐^8^O** was dissolved in o‐dichlorobenzene. For pristine OECTs, a hot solution (110 °C) at 12 mg mL^−1^ was spin‐coated onto RT cleaned substrates (1000 rpm 90 s). Different thicknesses were obtained by diluting the stock solution to concentrations of 10 and 5 mg mL^−1^. No post‐deposition treatment was performed, unless for controlled experiments. For aligned OECTs, **PBTTT‐^8^O** hot solution (50 °C) at 10 mg mL^−1^ was doctor‐bladed onto 160 °C glass substrates covered with a thin sacrificial NaPSS layer, previously spin‐coated from a RT aqueous solution at 10 mg mL^−1^ (3000 rpm/60 s). The alignment was performed by high‐temperature rubbing using a home‐made equipment consisting of a rotating cylinder covered with a microfiber cloth while the sample was mounted on a translating heating stage. The best orientation was observed for the rubbing temperature T_R_ = 170 °C (pressure of 2–3 bar, 600 RPM, microfiber cloth).^[^ ^61^ ^]^ The aligned films were then floated onto deionized water, recovered on the pre‐patterned Cr/Au OECT substrates, and left to entirely dry in ambient. No post‐transfer treatment was performed. The films were partly removed using a high‐precision Q‐tip to delimit the polymer channels (dimensions given in Supporting Information). The DS contacts were passivated with Kapton tape to reduce current leakage through the electrolyte. The samples were sealed with a pseudo‐reference Ag/AgCl gate electrode in a home‐made spectroelectrochemical cell. Freshly prepared KPF_6_ electrolyte in bi‐distilled water (0.1 mol L^−1^) was then injected to complete the OECTs for all experiments. Note that the channel length is 2 mm. Decreasing the channel length would theoretically decrease the contact resistances and lead to a further increase in g_m_.^[^ ^30^ ^]^ Manufacture of OECTs with 10 µm‐long channels is reported in note S1. Unfortunately, the channel is damaged during the peeling‐off of the sacrificial layer, which prevented the appropriate characterization of these devices (Figure S5, Supporting Information). Optimizing the miniaturization and biocompatibility of aligned OECTs is the scope of a follow‐up study.

### OECT Characterization

Transfer and output characteristics, chronoamperometry, and time‐resolved pulsing stability coupled with Vis‐NIR spectroelectrochemistry were performed using a home‐built setup controlled by LabVIEW. For transfer and output characteristics, a Keithley 2400 (Tektronix) was used to apply the V_DS_ voltage and probe the I_DS_ current. In all cases, the V_GS_ voltage was applied and measured with a data acquisition card (USB‐6211, National Instruments). For chronoamperometry, the I_GS_ current was converted into a voltage using a low‐noise current preamplifier (SR570, Stanford Research Systems) and recorded with the data acquisition card (time‐resolution of 8 𝜇s). The Vis‐NIR absorbance spectra were acquired with a Flame UV–vis spectrometer and a FlameNIR spectrometer (Ocean Optics) triggered by the data acquisition card. The incident white light was generated by a halogen light source (HL‐2000, Ocean Optics). It was collimated through a glass slide (0.55 mm), 2 mm of the electrolyte, the transistor channel, and the S151 slide (1.1 mm). See notes S3–S5 for details. For the spectral measurements, the electrolyte was degassed to avoid underlying signatures due to oxygen doping.

### Direct Mobility Measurements

The impedance matching measurements were conducted following a previously reported procedure.^[^ ^37^ ^]^ Briefly, a constant *V*_DS_ of −0.5 V and a sinusoidal *V*_GS_ signal of varying frequency with a baseline at −0.9 V and an amplitude of 10 mV were applied with a functional unit (PXIe‐4163, National Instruments). The *I_GS_* and *I_DS_* currents were recorded with the same unit controlled by LabVIEW. The in situ electrochemical THz spectroscopy measurements were conducted using a home‐built setup described previously.^[^ ^51^ ^]^ The transmitted THz waveforms were acquired after a dwell time of 150 s at a doping voltage of −0.8 V and a dedoping voltage of +0.3 V for the reference. See note S6 for details.

## Conflict of Interest

The authors declare no conflict of interest.

## Author Contributions

O.B. designed and directed the study. P.D. and B. J. synthetized and characterized the **PBTTT‐^8^O** polymer. S.G. and V.B. manufactured and characterized the aligned films. O.B. manufactured the pristine and aligned OECTs. O.B., G.R., and J. R. developed the instrument coupling electrical transistor characterization and spectroelectrochemistry. W.E. performed the pulsing stability measurements. A.P. performed the CV stability experiment. H. W. and C. Y. performed the EIS and impedance matching experiments and analyzed the data. I.H. performed the THz spectroscopy experiments and analyzed the data. P.C. ran preliminary tests to accurately perform chronoamperometry. O.B. performed all the other experiments and analyzed the data. All authors contributed to data interpretation. O.B. and N.B. wrote the manuscript with inputs from S. F., M.B. and N.L. All authors have given approval to the final version of the manuscript.

## Supporting information



Supporting Information

## Data Availability

The data that support the findings of this work are available as open access in the BORIS repository of the University of Bern at https://boris‐portal.unibe.ch/handle/20.500.12422/210999.
