# Supplementary material for: Over Tenfold Increase in Current Amplification Due to Anisotropic Polymer Chain Alignment in Organic Electrochemical Transistors
Source: Adv Mater. 2025 Jun 2;37(32):2420323. doi: 10.1002/adma.202420323 (PMC12355448; doi:10.1002/adma.202420323)
Supplement: Supplementary file 1 — Supporting Information [file ADMA-37-2420323-s001.pdf]

ADVANCED MATERIALS

Supporting Information

for *Adv. Mater.*, DOI 10.1002/adma.202420323

Over Tenfold Increase in Current Amplification Due to Anisotropic Polymer Chain Alignment in Organic Electrochemical Transistors

Olivier Bardagot, Pablo Durand, Shubhradip Guchait, Han-Yan Wu, Isabelle Heinzen, Wissal Errafi, Victor Bouylout, Alessandra Pistillo, Chi-Yuan Yang, Gonzague Rebetez, Priscila Cavassin, Badr Jismy, Julien Réhault, Simone Fabiano, Martin Brinkmann, Nicolas Leclerc and Natalie Banerji**

SUPPLEMENTARY INFORMATION

Over 10-fold increase in current amplification due to anisotropic polymer chain alignment in organic electrochemical transistors

Olivier Bardagot, Pablo Durand, Shubhradip Guchait, Han-Yan Wu, Isabelle Heinzein, Wissal Errafi, Victor Bouylout, Alessandra Pistillo, Chi-Yuan Yang, Gonzague Rebetez, Priscila Cavassin, Badr Jismy, Julien Réhault, Simone Fabiano, Martin Brinkmann, Nicolas Leclerc, and Natalie Banerji**

*Corresponding author emails: olivier.bardagot@cnrs.fr, natalie.banerji@unibe.ch

This Supplementary Information includes:

Supplementary Figures 2

Supplementary Tables 26

Supplementary Notes 33

Note S1. OECT fabrication. 33

Note S2. Thickness determination of aligned films. 34

Note S3. Steady-state Vis-NIR spectroelectrochemistry 35

Note S4. Output characteristics, transfer characteristics and Bernards-Malliaras model 35

Note S5. Determination of *C**. 37

Note S6. Determination of μ_{hole}. 39

i. Inferred from μC^* and C^* 39

ii. Inferred from the slope of $\sqrt{I_{DS}^{Sat}}$ vs. V_{GS} plot and C^* 39

iii. Directly measured from impedance matching 39

iv. Directly measured from *in-situ* THz spectroscopy 40

Note S7. Determination of doping, dedoping, ON and OFF time constants 42

Note S8. Cyclic voltammetry 44

References 44

Supplementary Figures

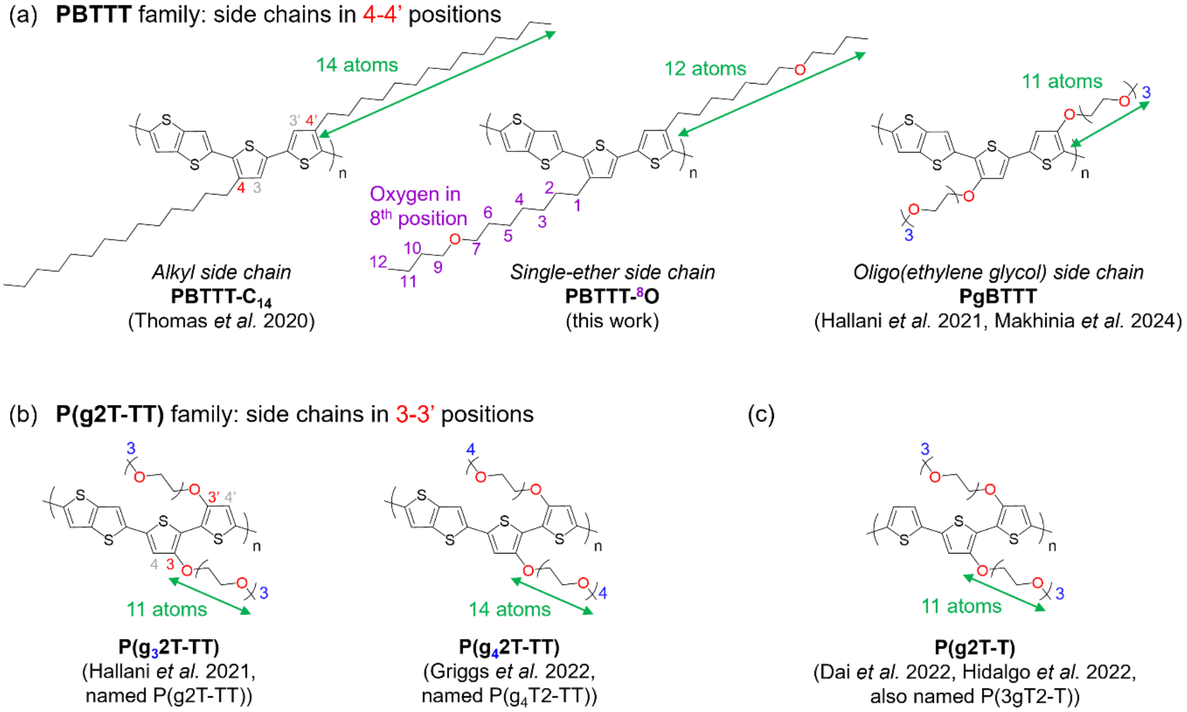

Figure S1. Chemical structures of the polymers discussed in the main text. (a) PBTTT family, (b) P(2T-TT) family, (c) P(2T-T) family. Note that in case of single-ether side chains, the position of the oxygen along the chain plays a role in the OECT properties. Luscombe, Ginger, *and coll.* demonstrated that placing the oxygen in the 8th position compared to the 2nd position leads to enhanced passive swelling, higher *C**, and faster electrochemical (de)doping kinetics for P3HT-analogues.[1]

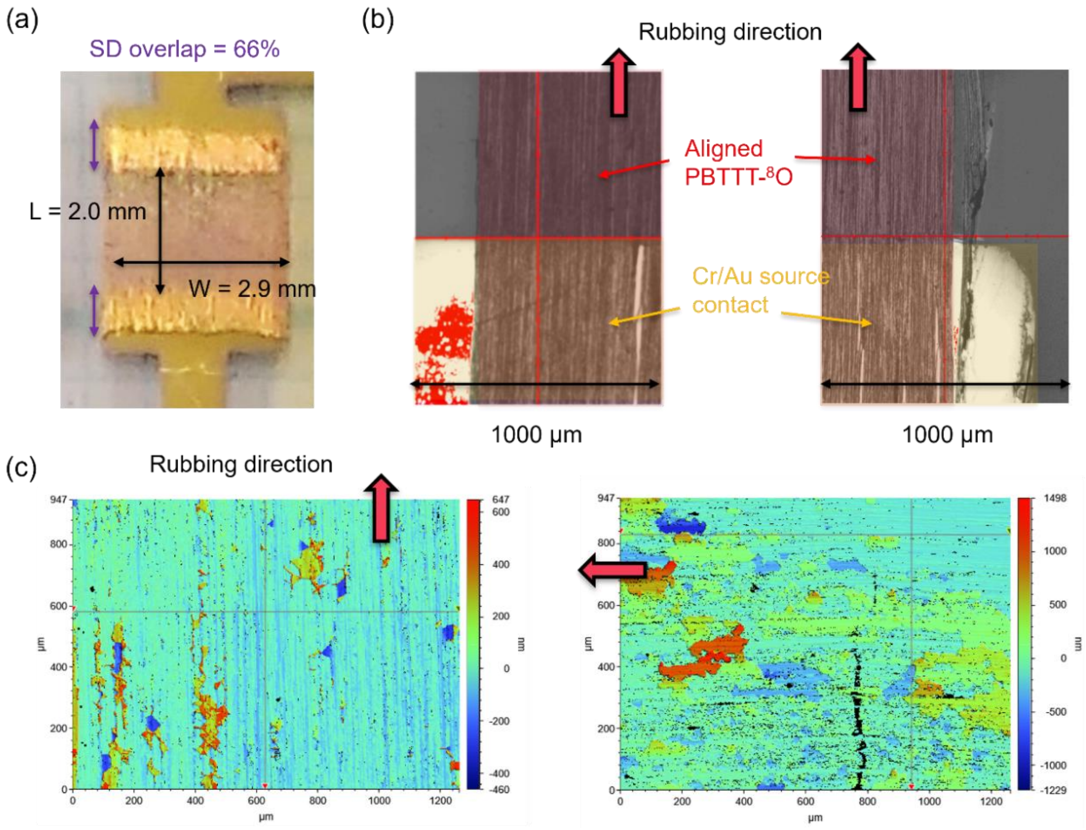

Figure S2. Images of mm-long aligned OECTs illustrating that the mechanical rubbing does not only order polymer chains at a nm-scale but also create ‘hills’ and ‘grooves’ at a µm-scale (see SEM images in **Figure S3**), hence increasing the surface/volume ratio compared to pristine films (see AFM images in **Figure S4**). (a) Picture of the aligned channel displaying the L and W dimensions, and the source-drain (SD) contact overlap (ratio of the area of polymer covering the S and D contacts over the area of the channel LxW). (b) Optical microscope images of two distinct samples highlighting the very accurate perpendicular alignment of the polymer chains regarding to the edge of the source contact. (c) White-light interferometry images illustrating the ‘hills’ and ‘grooves’ at a µm-scale formed by mechanical rubbing. Note that the polarization of the light caused by the polymer alignment hampers the quality of interferometry images regardless of the orientation of the sample.

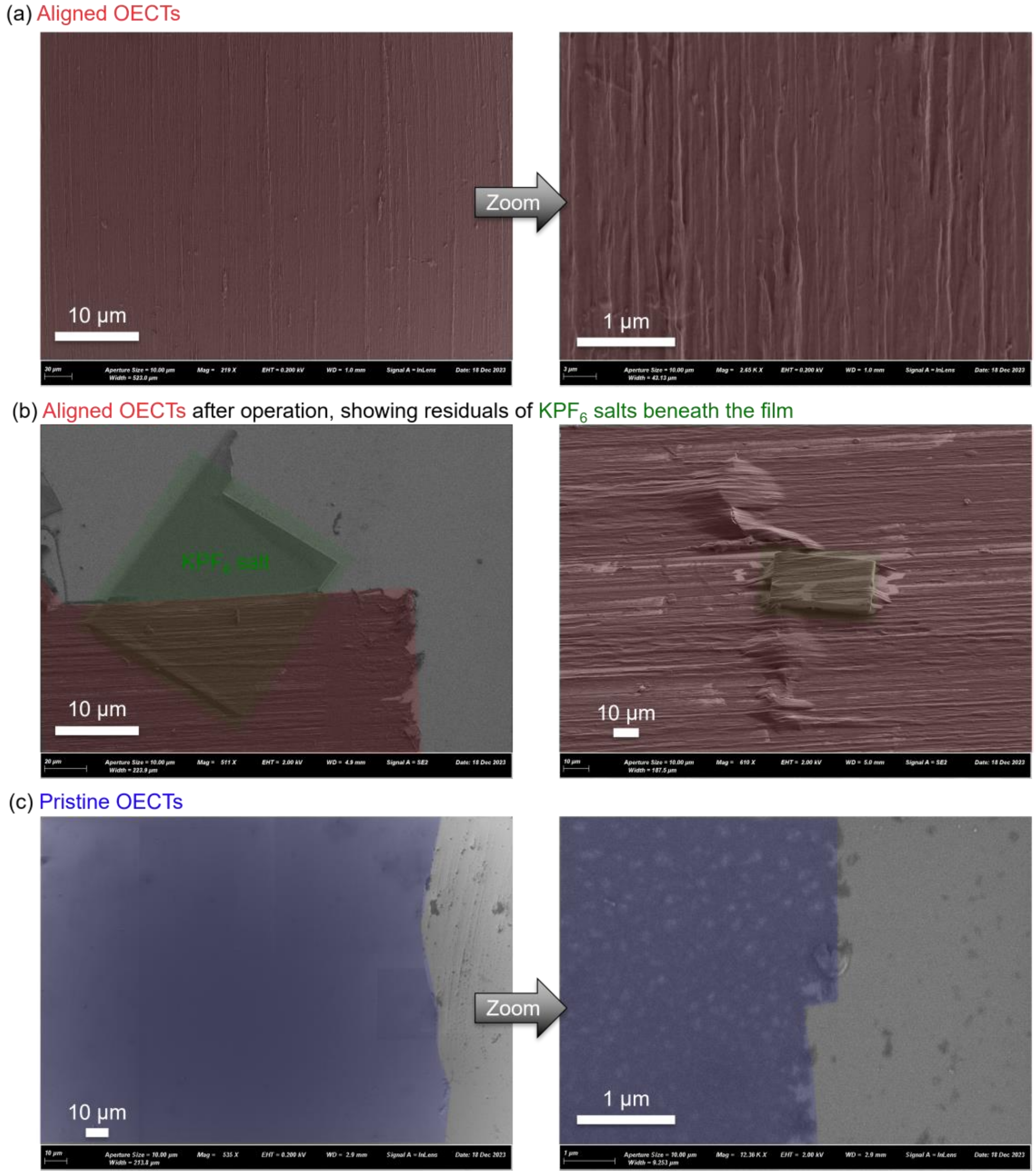

Figure S3. Scanning Electron Microscope (SEM) images of (a-b) aligned **PBTTT-⁸O** films (red) (a) before showing an observable anisotropy at the µm-scale, and (b) after testing in 0.1M KPF_6/H_2O electrolyte showing residuals of KPF_6 salts beneath the film (green) supporting doping through the entire film thickness. (c) SEM images of pristine **PBTTT-⁸O** films (blue) showing no significant feature.

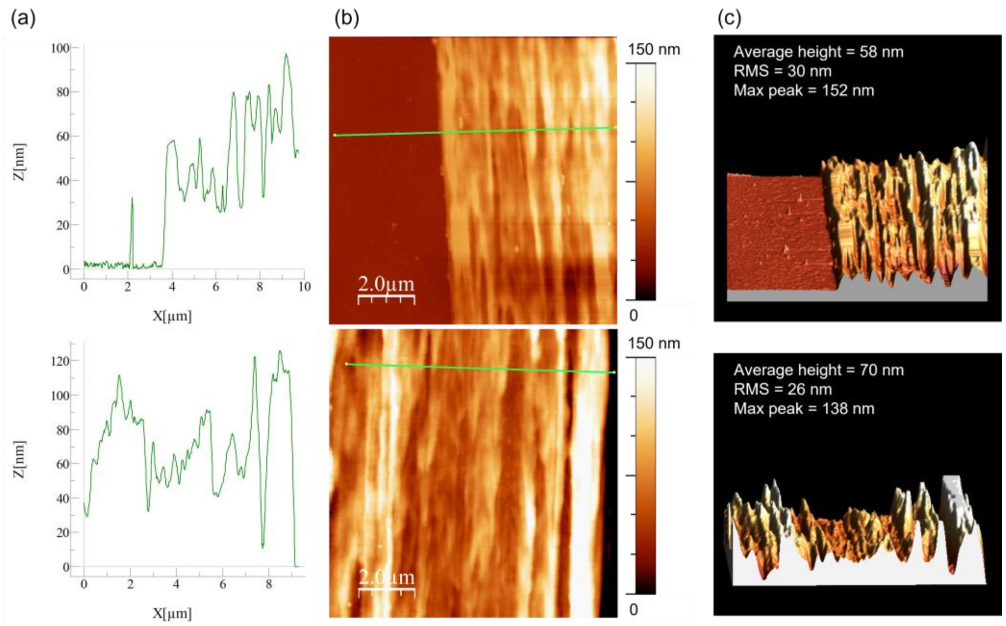

Figure S4. Atomic Force Microscope (AFM) images of aligned PBTTT-^{8}O films. (a) Height profile. (b) 10 x 10 μm^2 height images. (c) 3D views highlighting the bulk effect of the high-temperature mechanical rubbing technique *for such film thickness* and the resulting high roughness of the aligned films (root mean square (RMS) roughness of 37-52% of the average thickness).

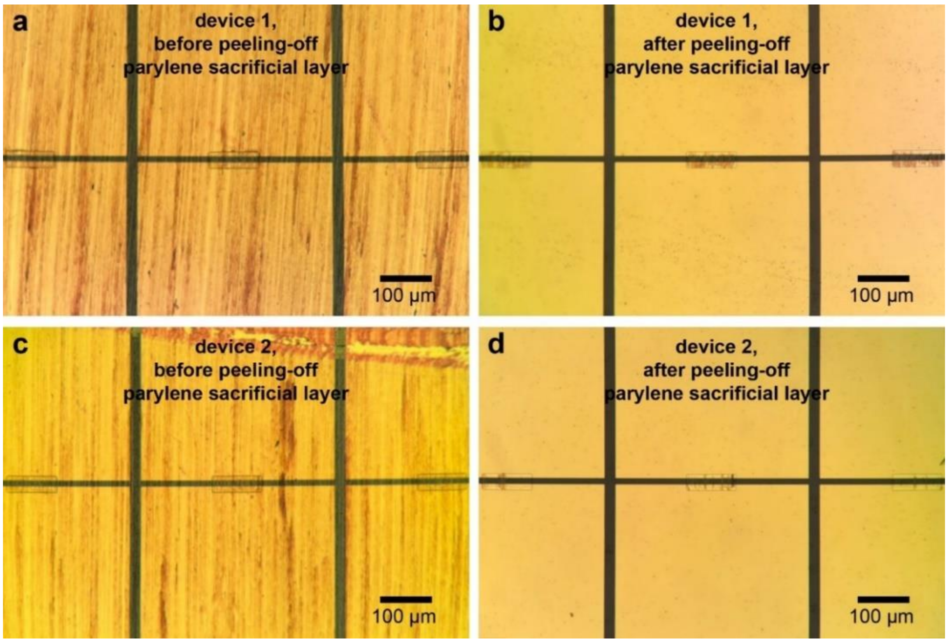

Figure S5. Microscopic images of μm-long aligned OECTs (W/L = 100 μm/10 μm). (a) Device 1, before peeling-off, (b) device 1, after peeling-off, (c) device2, before peeling-off, (d) device 2, after peeling-off. The material coverage within the channel is damaged during the peeling-off of the sacrificial layer, which prevented appropriate characterization of these devices.

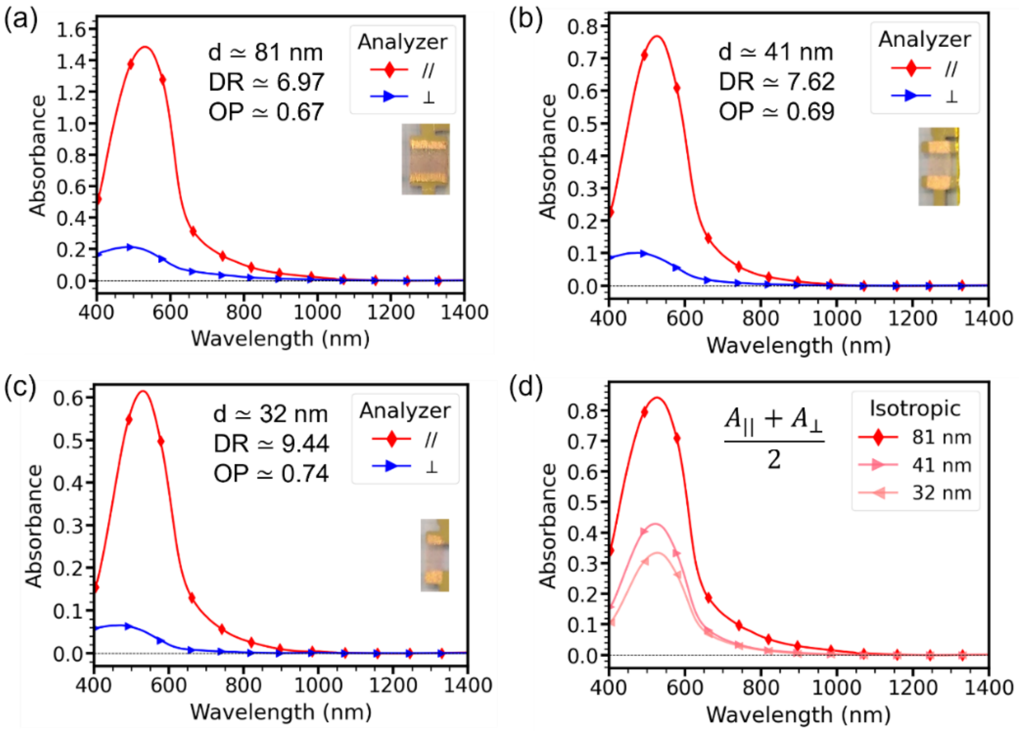

Figure S6. Polarized Vis-NIR absorbance spectra of dry aligned films of PBTTT-^{8}O. (a-c) with an analyzer parallel (//) or perpendicular (⊥) to the rubbing direction. Inset: average dry thickness (d), dichroic ratio (DR), and 3D order parameter (OP) calculating from: $DR = \frac{A_{\parallel}}{A_{\perp}}$ and $OP = \frac{\mathrm{DR}-1}{\mathrm{DR}+2}$.[2] (d) Reconstructed isotropic spectra.

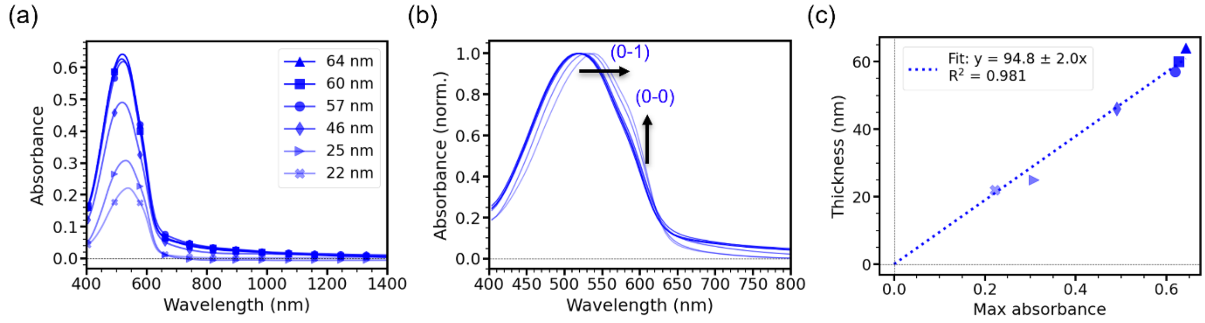

Figure S7. Vis-NIR absorbance spectra of dry pristine films of **PBTTT-^{8}O** spin-coated from 110 °C o-dichlorobenzene solutions. (a) Relative absorbance highlighting the difference thicknesses obtained and (b) normalized absorbance highlighting the bathochromic 0-1 shift and hyperchromic 0-0 shift of the thinner films. (c) Resulting absorbance-thickness calibration curve (dotted line = linear fit).

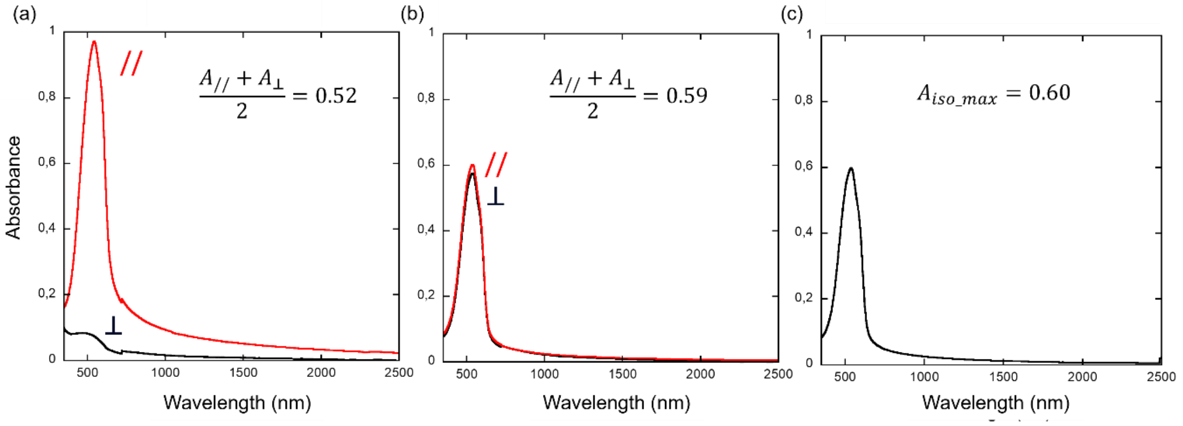

Figure S8. Polarized Vis-NIR spectra recorded for light polarized parallel (//) and perpendicular (⊥) to the rubbing direction for (a) as-rubbed aligned PBTTT-^{8}O (T_R =170°C) and (b) the same sample after thermal annealing to randomize the in-plane chain orientation (350°C, 5 min). For the sake of comparison, we show in (c) the Vis-NIR absorbance of the isotropized sample recorded with unpolarized light.

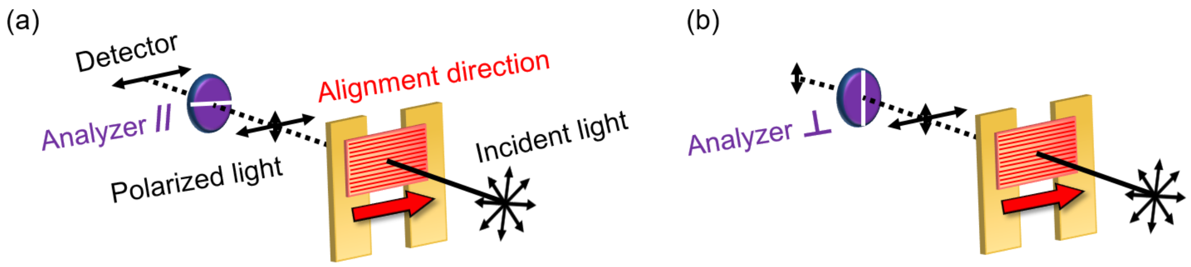

Figure S9. Illustration of polarized absorbance spectroscopy with (a) an analyzer parallel (//) and (b) perpendicular (⊥) to the rubbing direction.

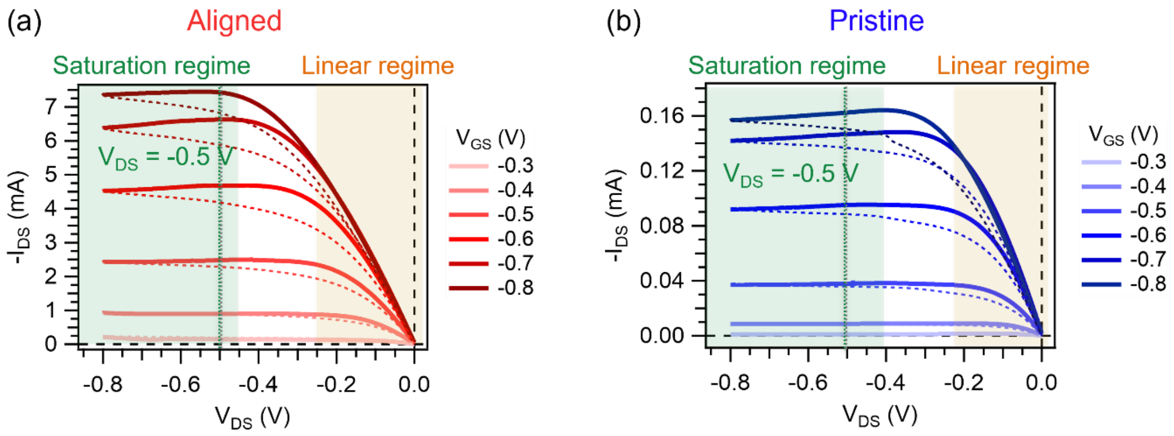

Figure S10. Output characteristics of (a) aligned vs. (b) pristine **PBTTT-^{8}O** OECTs in degassed 0.1 M KPF_6/H_2O electrolyte. V_{DS} was swept at 2 mV s^{-1} with fixed V_{GS} from -0.3 V to -0.8 V (solid lines) and from -0.8 V to -0.3 V (dashed lines). The channel geometric factors

(Wd/L) of the aligned and pristine OECTs are 92 nm and 30 nm, respectively. These dimensions translate into maximum I_{DS} currents of about 7.5 mA and 0.17 mA, respectively, indicating a **14× enhancement** when normalized by Wd/L. The fixed V_{DS} used to perform the transfer characteristics is represented by a vertical green dotted line to highlight that the transistor operates in saturation regime (green region).

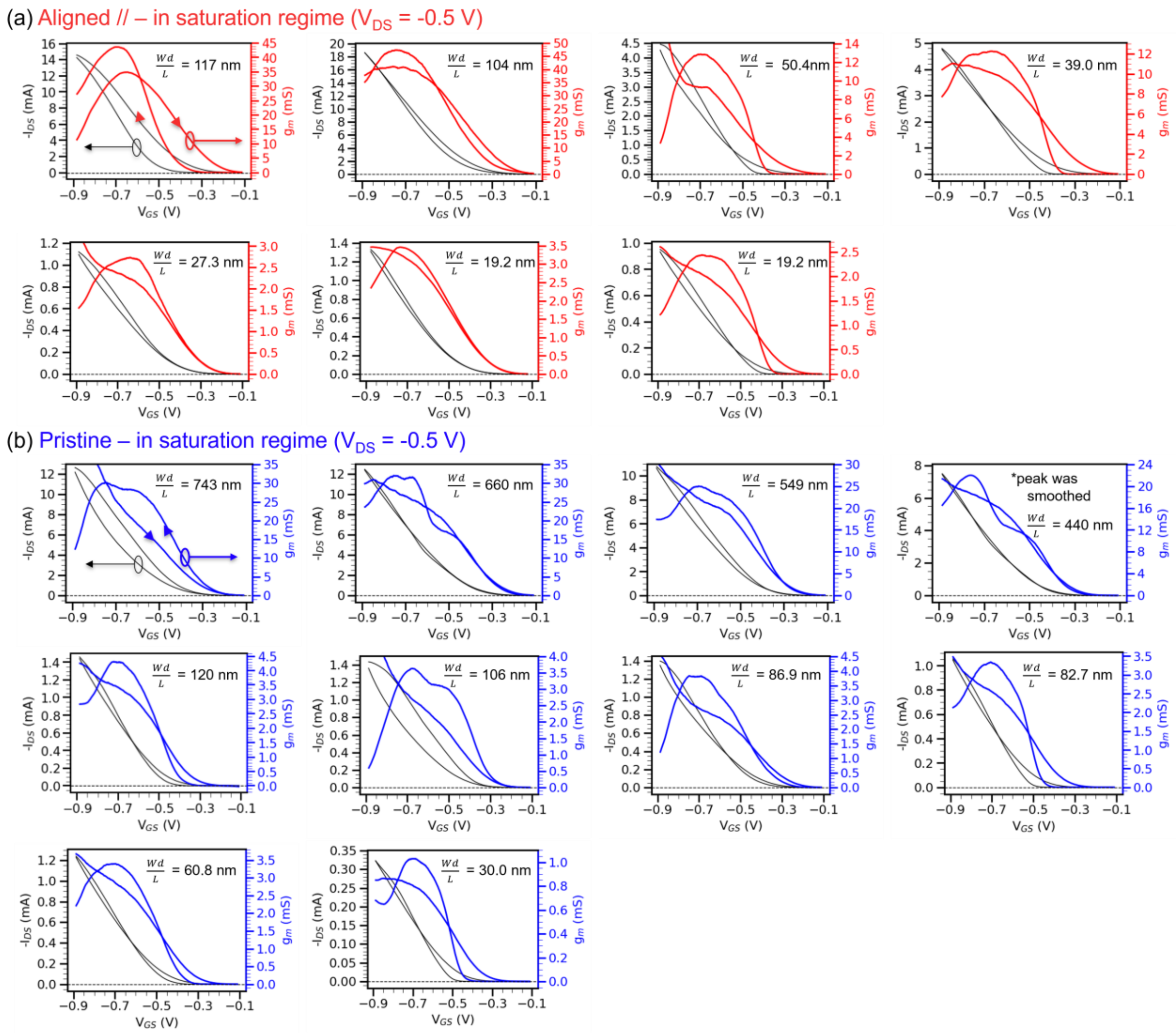

Figure S11. Transfer characteristics of (a) aligned and (b) pristine **PBTTT-**$^{\mathbf{8}}$**O** OECTs in degassed 0.1 M KPF_6/H_2O electrolyte. V_{GS} was swept back and forth at 1 or 2 mV s^{-1} with constant V_{DS} = -0.5 V to be in the saturation regime (**Figure S10**). I_{DS} data points are averaged every 30 points and the curves are smoothed using a Savitzky–Golay filter in Python (window length of 101, polynomial order of 1). g_m is calculated as the derivative of the resulting I_{DS}^{Sat} curve over V_{GS}, according to equation (2). Inset: channel geometric factor Wd/L.

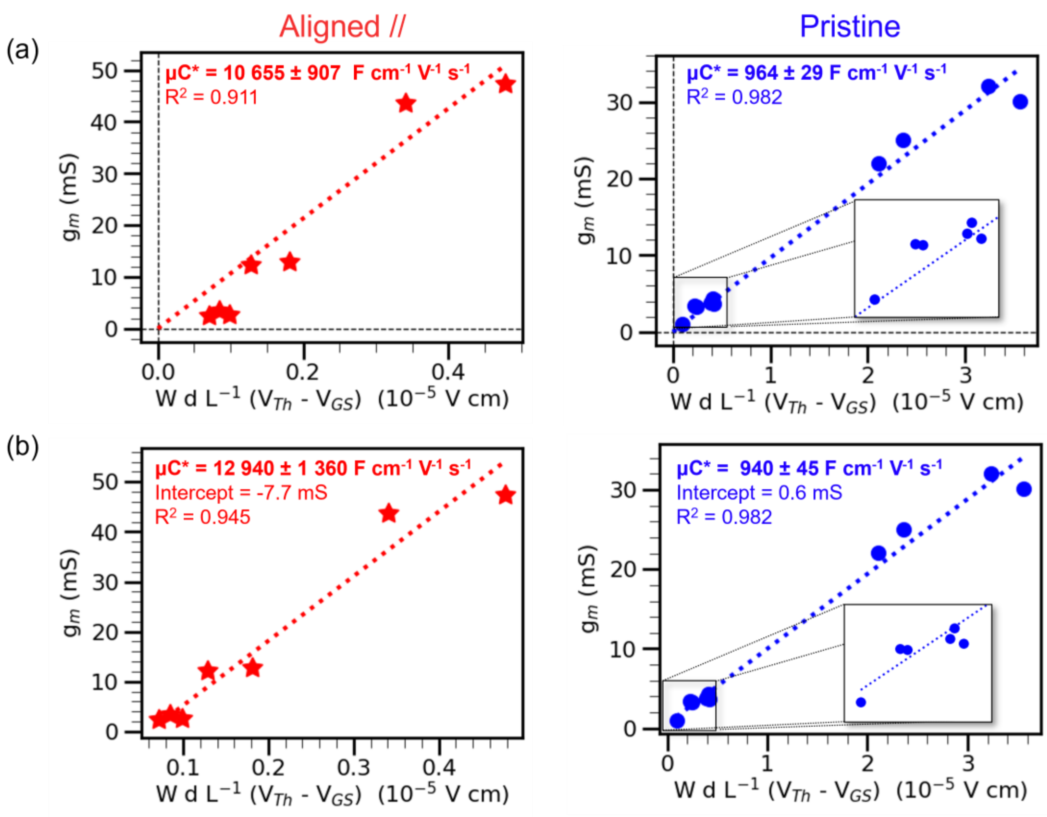

Figure S12. Comparison of the fitting methods to extract *μC**. (left, red) Aligned vs. (right, blue) pristine **PBTTT-^{8}O**. (a) y = ax, *with zero intercept (b = 0)* following the Bernards-Malliaras model, as commonly done in literature.[3] (b) y = ax + b, *with non-zero intercept (b ≠ 0)*. Dashed lines are the linear fits. The error corresponds to one standard deviation. Satisfying R^2 over 0.91 are obtained. The slightly better fit achieved for y = ax + b suggests the assumptions of the Bernards-Malliaras model of zero contact resistance and the channel resistance being the reciprocal of the gate voltage are not satisfied.[4] Keeping this in mind, it is extracted here for quantifying the **11× enhancement** achieved by alignment and for comparing the results with the literature.

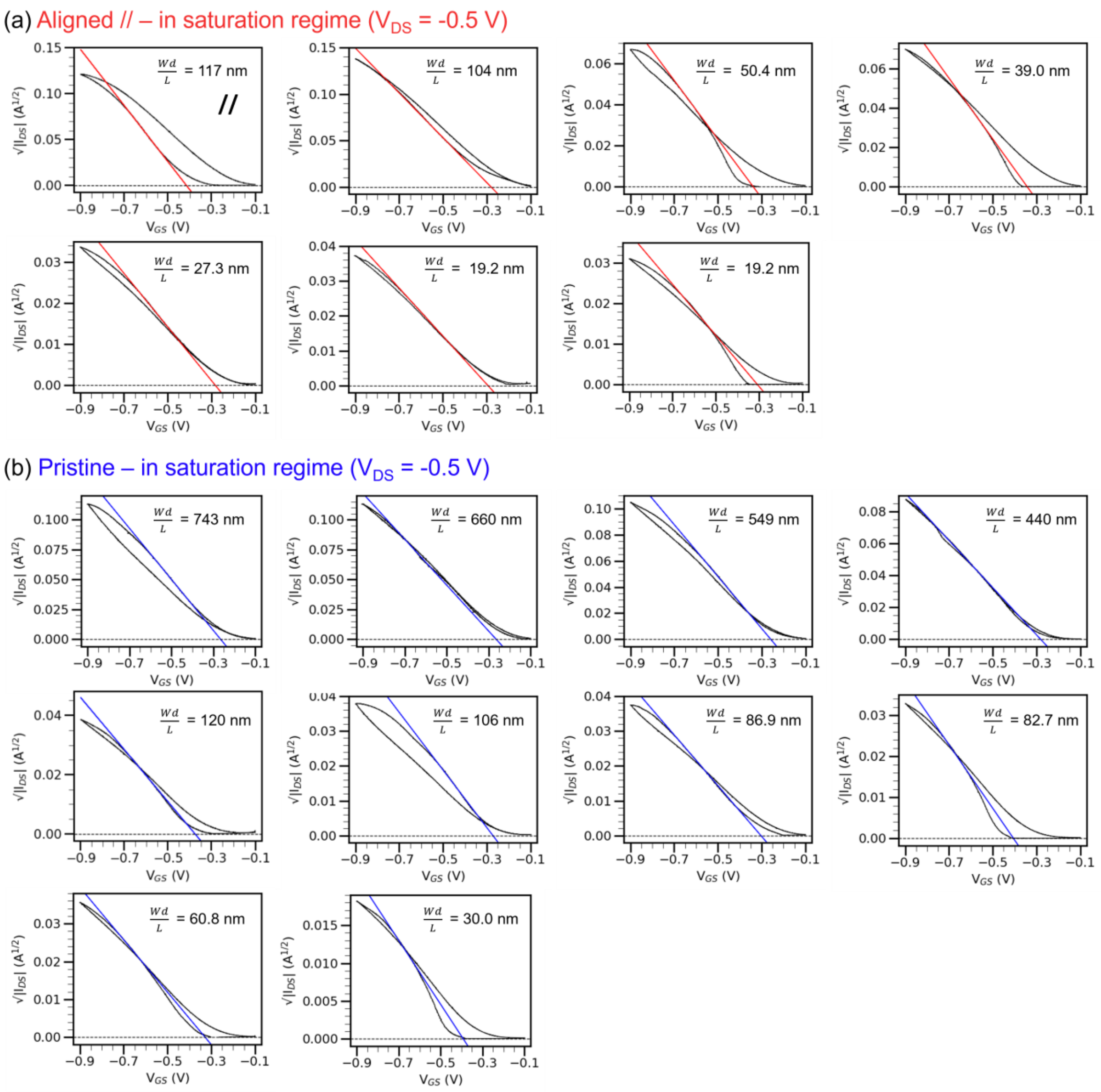

Figure S13. Transfer characteristics: $\sqrt{I_{DS}^{Sat}}$ vs V_{GS} plots. V_{Th} is defined as the x-intercept of the linear fits of the forward $\sqrt{I_{DS}^{Sat}}$ (colored lines), in accordance with the linear extrapolation method. Note that the non-ideality of the √ $\sqrt{I_{DS}^{Sat}}$ vs V_{GS} plot is predicted by the thermodynamic model of Cucchi et al.[5]

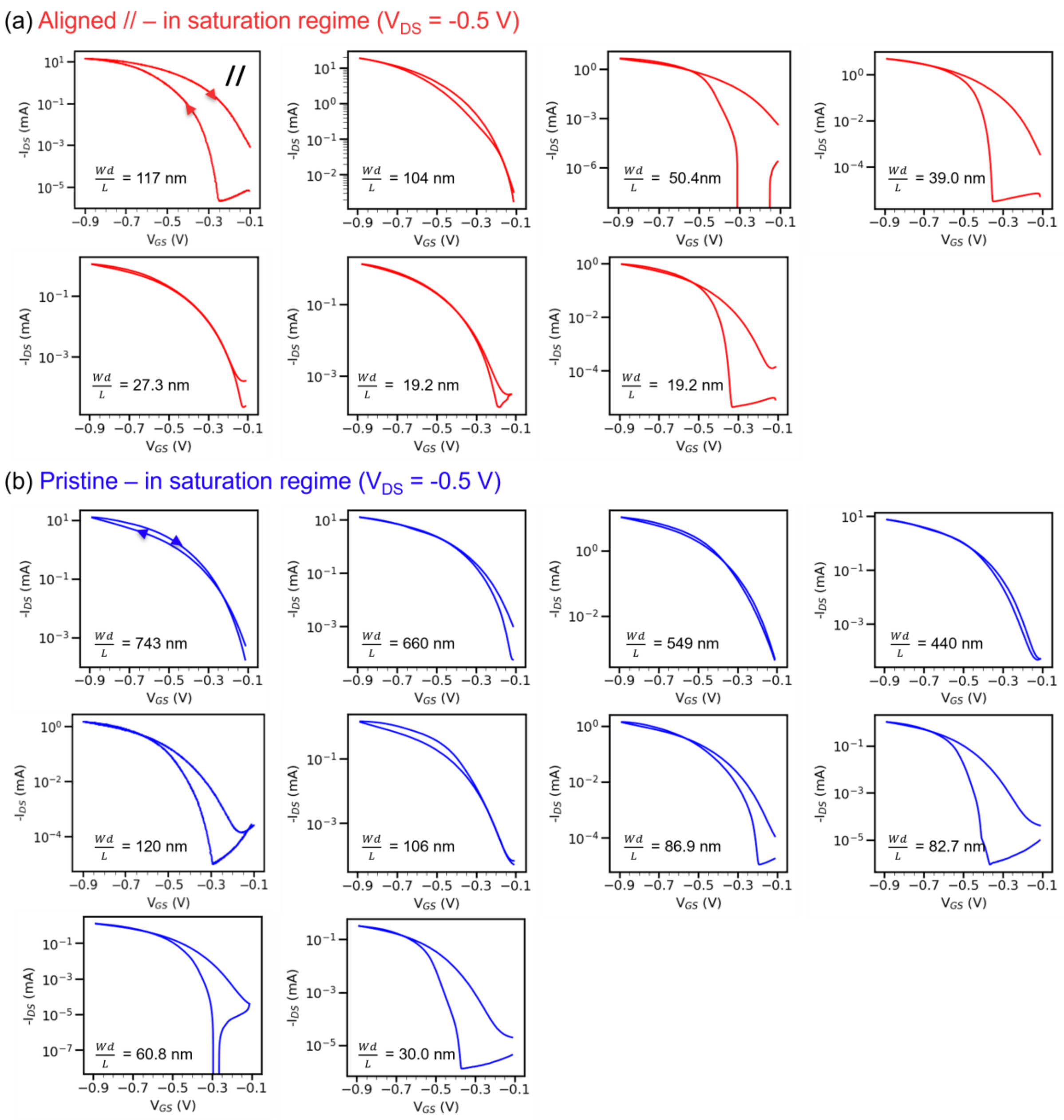

Figure S14. Transfer characteristics in logarithmic y-scale. Same data as **Figure S11** but plotted in log to highlight the I_{ON}/I_{OFF} ratio achieved for each device. Inset: channel geometric factor Wd/L.

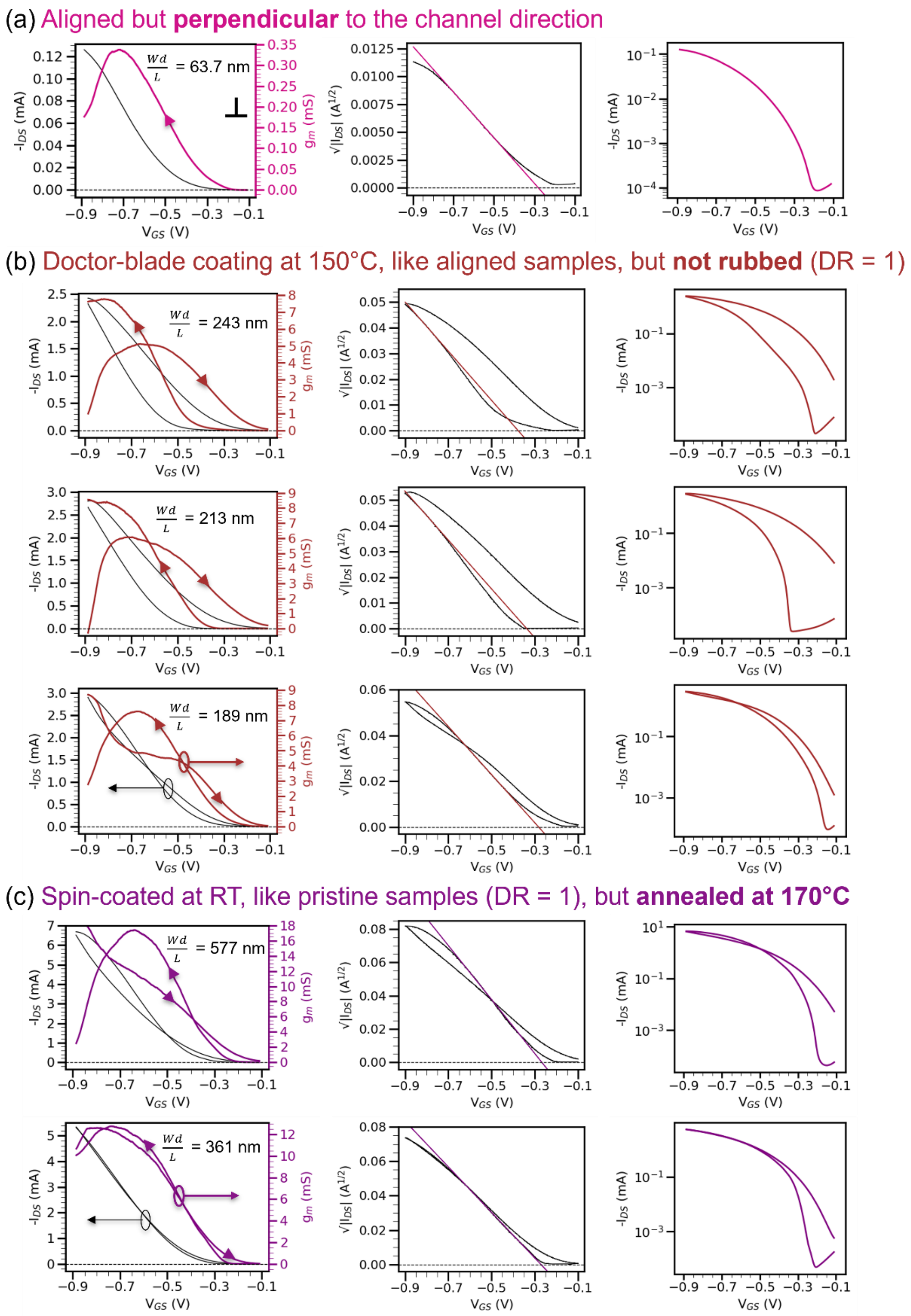

Figure S15. Control transfer characteristics. (a-b) Samples prepared by doctor-blade at 160 °C, as the aligned **PBTTT-^{8}O** OECTs, but (a) rubbed and deposited perpendicular to the channel direction (and not parallel to it), and (b) tested before rubbing (*i.e.*, DR = 1). (c) Pristine **PBTTT-^{8}O** OECTs (meaning spin-coated at 110 °C), but subjected to post-treatment annealing at 170 °C/10 min, like aligned samples. (left) Transfer characteristics, (middle) $\sqrt{I_{DS}^{Sat}}$ vs V_{GS} plot, (right) logarithmic y-scale to highlight the I_{ON}/I_{OFF} ratio.

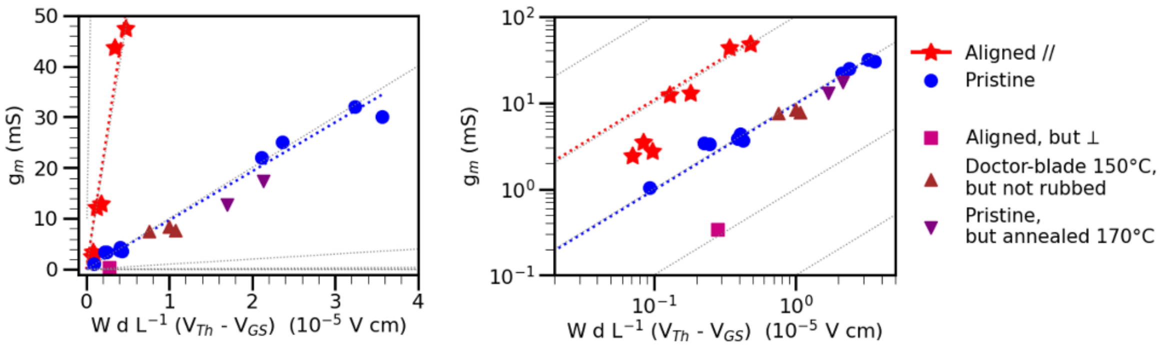

Figure S16. Comparison of μC* found for aligned // and pristine OECTs vs. the control experiments. Resulting transconductance g_m as a function of channel geometry and voltage parameters, underlining that (i) the alignment itself is not sufficient to afford high I_{DS} amplification, as the **perpendicularly aligned OECT performs two orders of magnitude less** (g_mL/Wd = 53 S cm^{-1}, pink square) **than when aligned parallelly** (g_mL/Wd = 2 580 ± 1 216 S cm^{-1}, red stars); and that (ii) **doctor-blade non-rubbed OECTs** (g_mL/Wd = 372 ± 37 S cm^{-1}, μC^* = 839 ± 63 F cm^{-1} V^{-1} s^{-1}, brown up triangles) and the **pristine post-deposition annealed at 170 °C /10 min OECTs** (g_mL/Wd = 330 ± 26 S cm^{-1}, μC^* = 792 ± 22 F cm^{-1} V^{-1} s^{-1}, purple down triangles) **both afford similar g_mL/Wd and μC^* than the pristine (spin-coated and non-annealed) OECTs** (g_mL/Wd = 430 ± 69 S cm^{-1}, μC^* = 964 ± 29 F cm^{-1} V^{-1} s^{-1}, purple blue circles). These results unequivocally **attribute the improved electrical performance** to the **high anisotropy**, **parallel to the channel direction,** of the aligned // OECTs, and ***not* to differences in sample manufacture** (doctor-bladed vs. spin-coated) or **beneficial recrystallization during annealing at 170 °C** (as-cast vs. rubbed at 170°C). Details are given in **Table S3**.

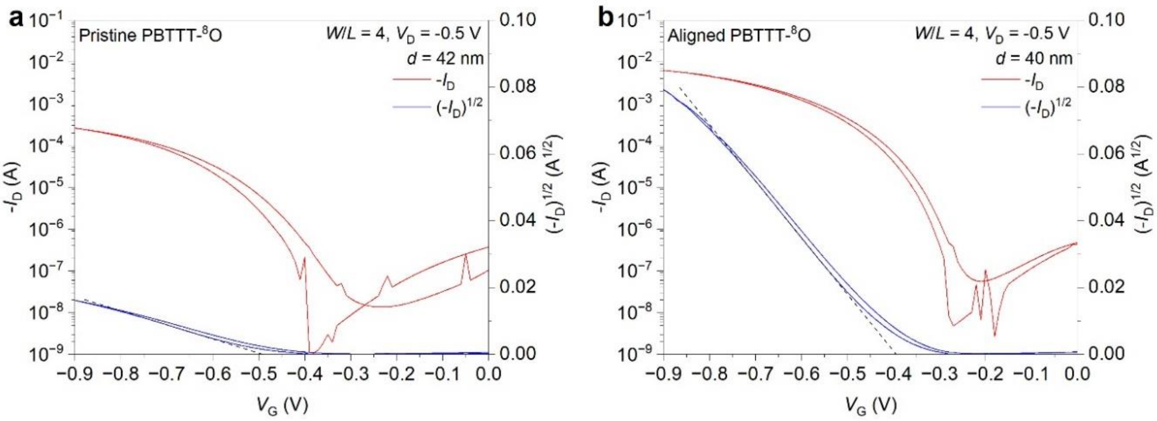

Figure S17. Transfer characteristics of 500-µm-long channel OECTs (L = 500 µm, W = 2000 µm). (a) Pristine and (b) aligned **PBTTT-^{8}O** OECT in degassed 0.1 M KPF_6/H_2O electrolyte. Both devices are made with channel thickness (d) of about 40 nm. For equivalent channel dimensions, the pristine OECT reaches a maximum I_{DS} of 0.26 mA, while the aligned OECT reaches a maximum I_{DS} of 6.1 mA at identical V_G = -0.9 V, which represents a **23× enhancement** of I_{DS} thanks to anisotropic alignment of the **PBTTT-^{8}O** chains. These data acquired on OECTs with 500-µm-long channels in a different laboratory confirms the benefit of alignment induced by high-temperature rubbing observed on OECTs with mm-long channels. After the characterization, the hole mobility is estimated from the slope of $\sqrt{I_{DS}^{Sat}}$ vs. V_{GS} as detailed in section **1.1.ii** below: for pristine OECTs, slope (dashed black line) = 0.042 $A^{1/2}$ V^{-1}, $Wd/2L\cdot\mu C^*$ = slope^2 = 0.00178 A V^{-2}, $\boldsymbol{C^*_{pristine}}$ **= 390 ± 40 F cm^{-3}**, implies a $\boldsymbol{\mu_{pristine}}$ **of 0.54 ± 0.06 cm^2 V^{-1} s^{-1}**; while for aligned OECTs, slope = 0.17 $A^{1/2}$ V^{-1}, $Wd/2L\cdot\mu C^*$ = slope^2 = 0.0289 A V^{-2}, $\boldsymbol{C^*_{aligned}}$ **= 560 ± 70 F cm^{-3}** , implies a $\boldsymbol{\mu_{aligned}}$ **= 6.5 ± 0.8 cm^2 V^{-1} s^{-1}**.

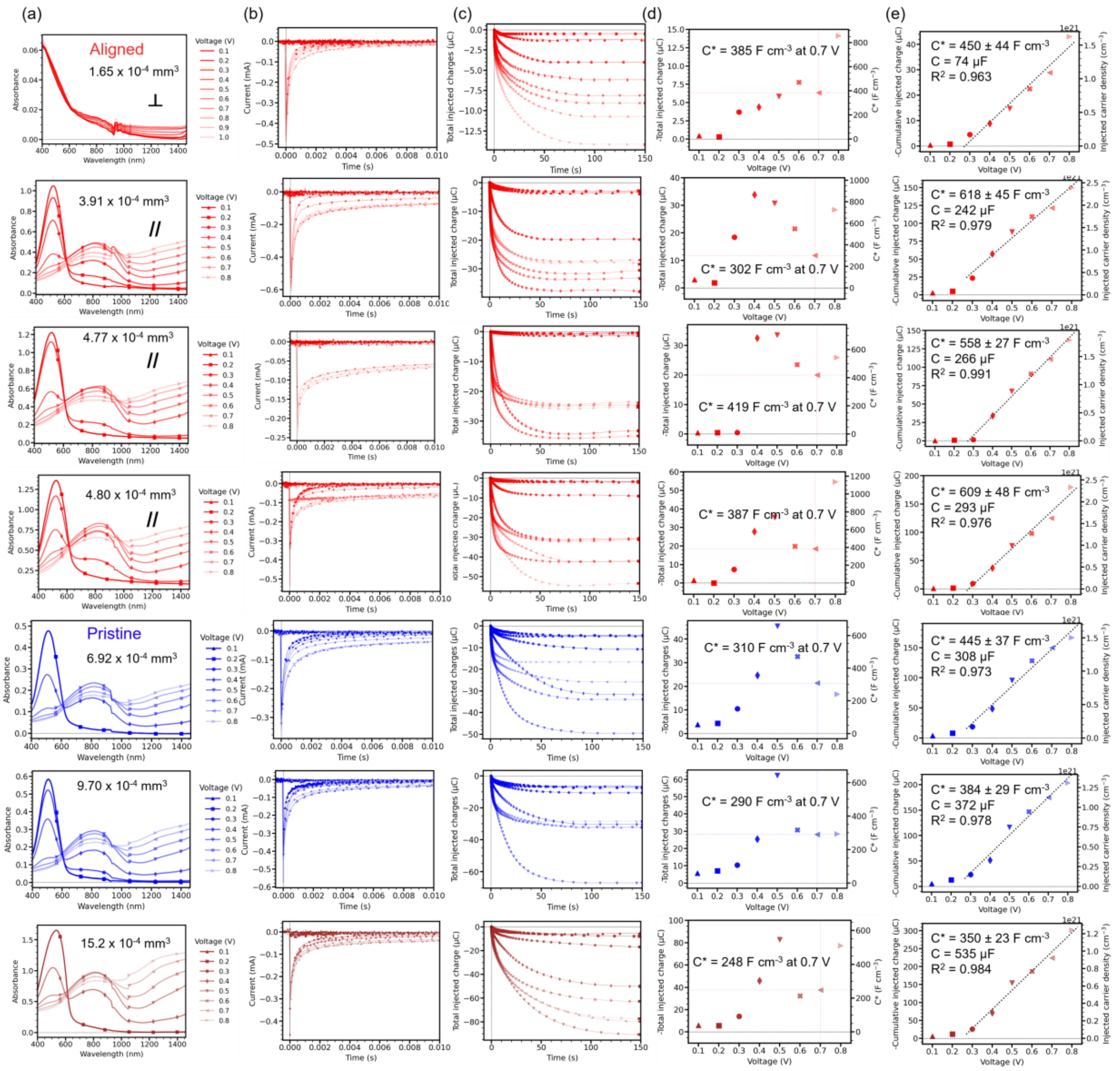

Figure S18. Chronoamperometry measurements coupled with Vis-NIR spectroelectrochemistry. (a) Polarized steady-state Vis-NIR absorbance spectra of (top x4, red) aligned and (bottom x3, blue and brown) pristine **PBTTT-^{8}O**. The absorbance of the first aligned sample is probed with an analyzer perpendicular (⊥) to the rubbing direction, while the three other samples are probed with an analyzer parallel (//) to the rubbing direction. Inset: Total film volume (*i.e.*, channel + SD overlap). (b) Corresponding temporal evolution of the gate current. The non-zero residual current reached in the steady state was averaged and subtracted to correct for constant electrical noise. (c) Integral of the current over time. The values reached at 150 s (steady state) gives the total number of charges injected upon each voltage step (ΔV = +0.1 V). (d) Corresponding total injected charge per voltage step. (e) Corresponding total cumulative injected charge as a function of the applied voltage. An average absolute capacitance C and the volumetric *C** over the doping voltage range is extracted from a linear fit (from +0.3 V to +0.8 V). The corresponding *C** values are reported in **Table S4**.

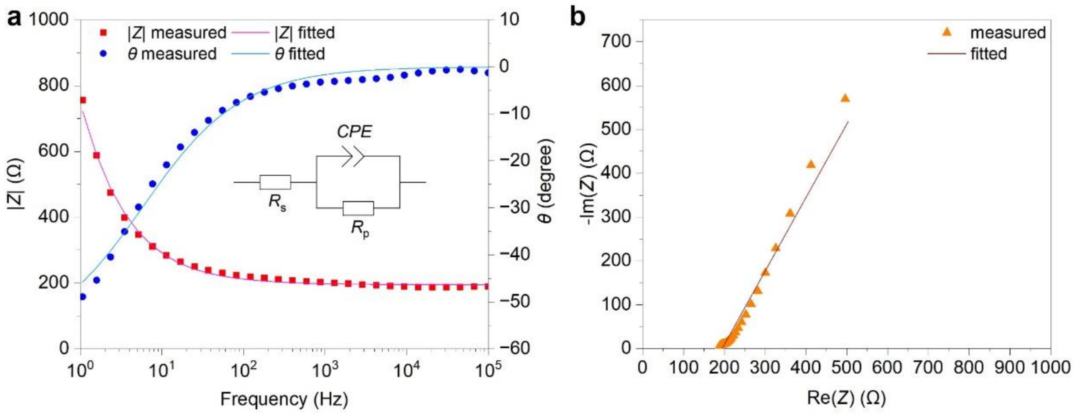

Figure S19. Electrochemical Impedance Spectroscopy (EIS) results. (a) Bode impedance plot and (b) Nyquist impedance plot. The capacitance was fitted to be 1.37×10^{-4} F, the volume of the pristine **PBTTT-^{8}O** film is 3.91×10^{-7} cm^3, resulting in a calculated C^* of 350 F cm^{-3} for this specific sample.

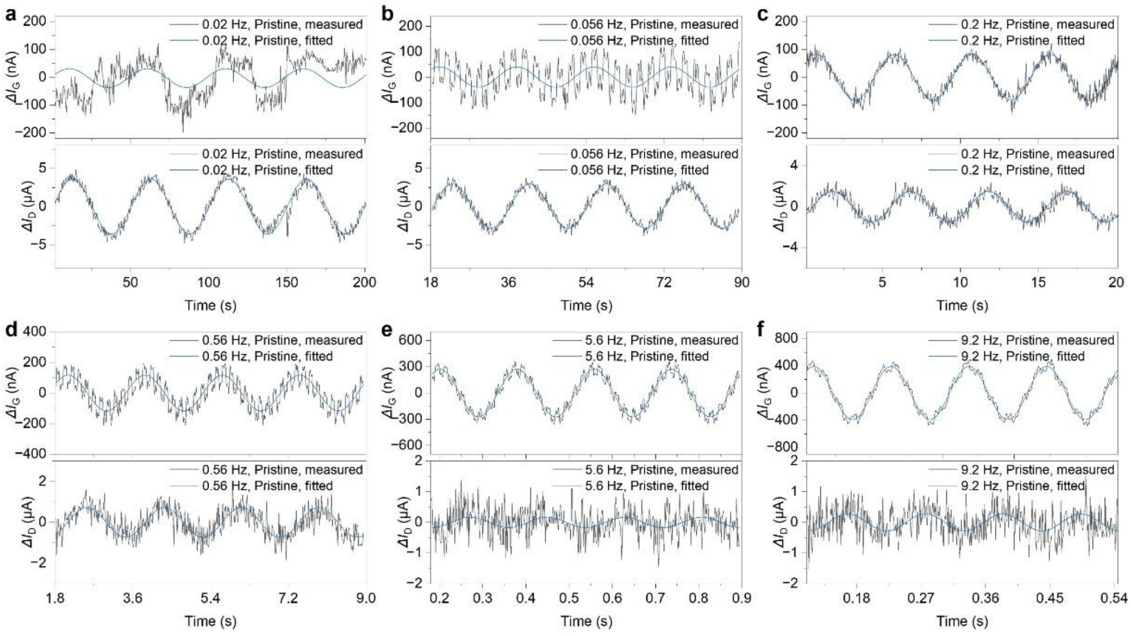

Figure S20. Impedance matching of pristine OECTs. (a-f) Sinusoidal ΔI_{GS} and ΔI_{DS} at frequencies of 0.02 Hz to 9.2 Hz. V_{DS} was biased to -0.5 V and V_{GS} was subjected to a sinusoidal signal with a baseline of -0.9 V and an amplitude of 10 mV. The frequency f of the sinusoidal V_{GS} voltage was swept from 0.010 Hz to 200 Hz. ΔI_{GS} at $f < 0.056$ Hz is lower than the detection limit of the instrument.

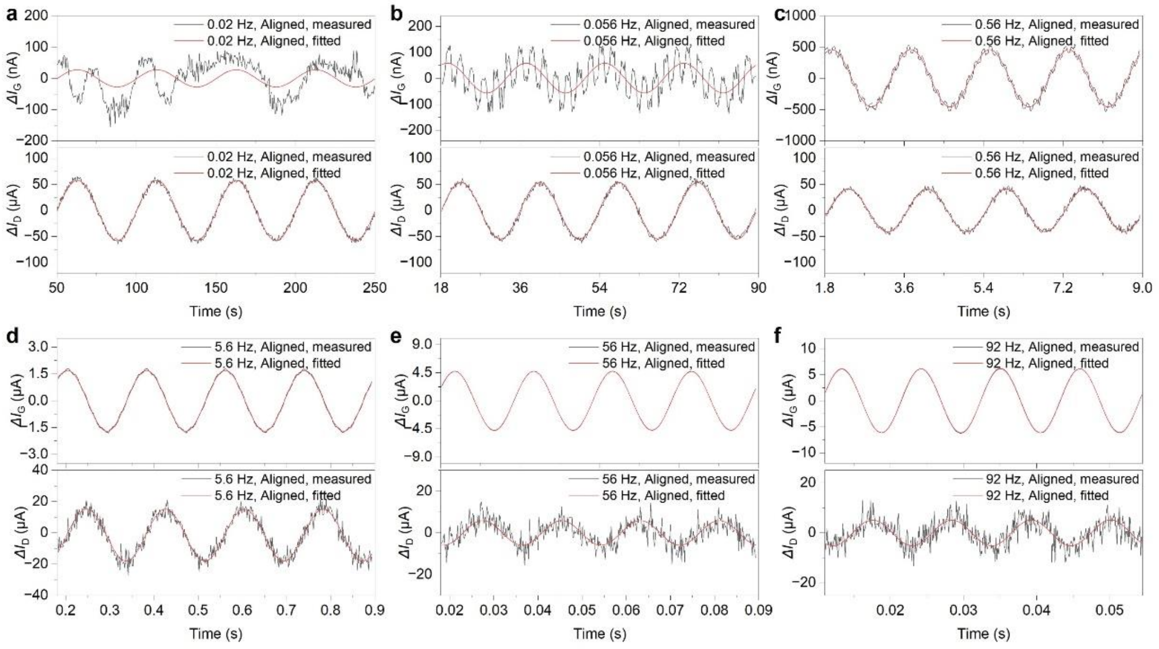

Figure S21. Impedance matching of aligned OECTs. (a-f) Sinusoidal ΔI_{GS} and ΔI_{DS} at frequency of 0.02-9.2 Hz, measured in the same conditions than pristine samples (**Figure S20**).

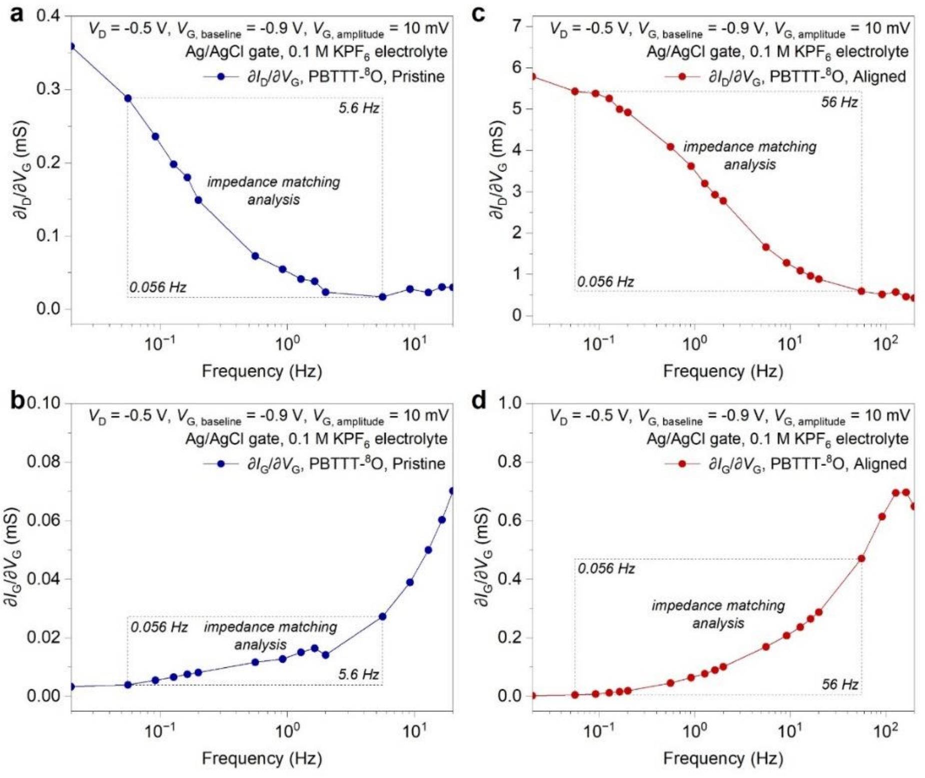

Figure S22. Frequency dependence of transconductance $\partial I_{\mathrm{D}}/\partial V_{\mathrm{G}}$ and gate conductance $\partial I_{\mathrm{G}}/\partial V_{\mathrm{G}}$ of (a, b) pristine and (c, d) aligned **PBTTT-^{8}O** OECTs. For **pristine**, data in the range of 0.056 Hz ≤ f ≤ 5.6 Hz fall within the detection limit of the instrument. For **aligned**, data in the range of 0.056 Hz ≤ f ≤ 56 Hz fall within the detection limit of the instrument.

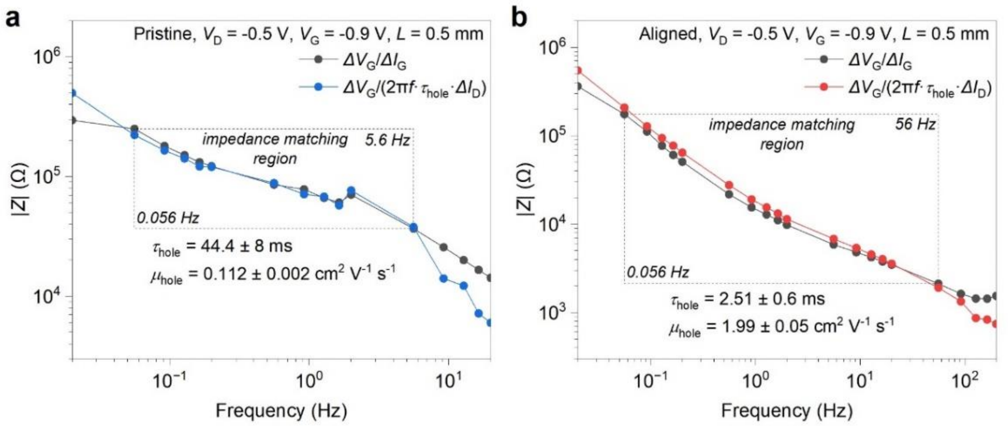

Figure S23. Frequency dependence of impedance |Z| of (a) pristine and (b) aligned **PBTTT-^{8}O** OECTs. All the fitting and calculation are based on the consistent part of the data (shown in dashed-line box).

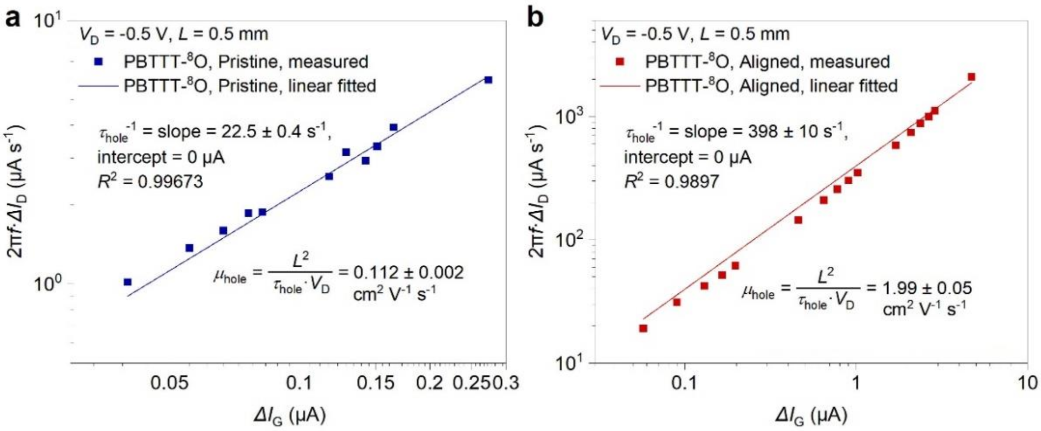

Figure S24. Linear fitting of τ_{hole} of (a) pristine and (b) aligned **PBTTT-^{8}O** OECTs. The τ_{hole} of **pristine** is calculated to be 44.4 ms, whereas for **aligned** it is 2.51 ms. With L = 500 μm, the hole mobilities extracted by impedance matching are thus $\boldsymbol{\mu_{pristine}}$ **= 0.11 cm^2 V^{-1} s^{-1}** and $\boldsymbol{\mu_{aligned}}$ **= 2.0 cm^2 V^{-1} s^{-1}** for pristine and aligned devices, respectively.

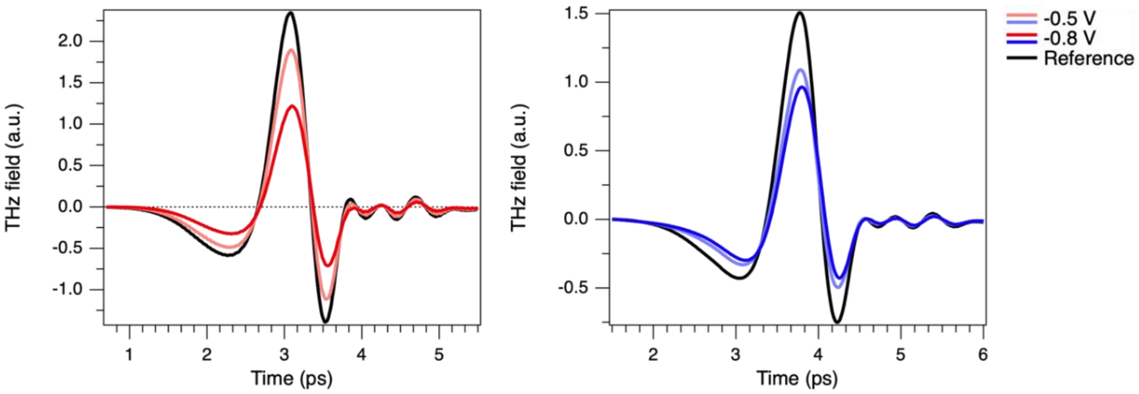

Figure S25. THz waveform of (left, red) aligned and (right, blue) pristine **PBTTT-^{8}O** measured at -0.5 V and -0.8 V. The black curves correspond to the respective dedoped states measured at +0.3 V and used as reference. All data are measured for SD short-circuited OECTs with a channel length of 3 mm.

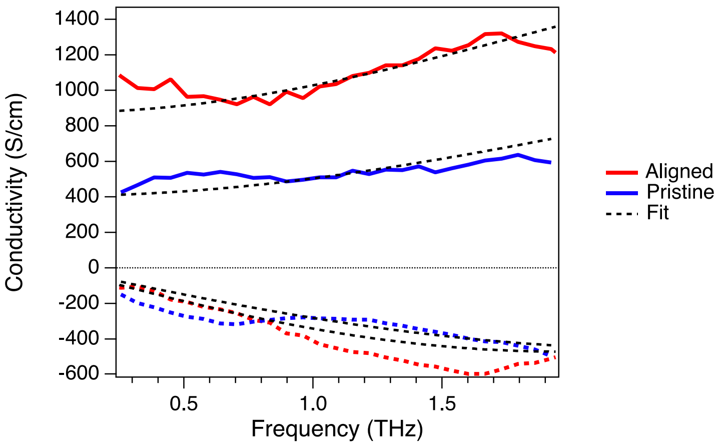

Figure S26. Complex THz conductivity spectra of (red) aligned and (blue) pristine **PBTTT-^{8}O** OECTs at a doping voltage of -0.8 V. The real conductivities are positive and presented as solid lines. The imaginary conductivities are negative and presented as dashed lines. The black dashed lines correspond to the Drude-Smith fit.

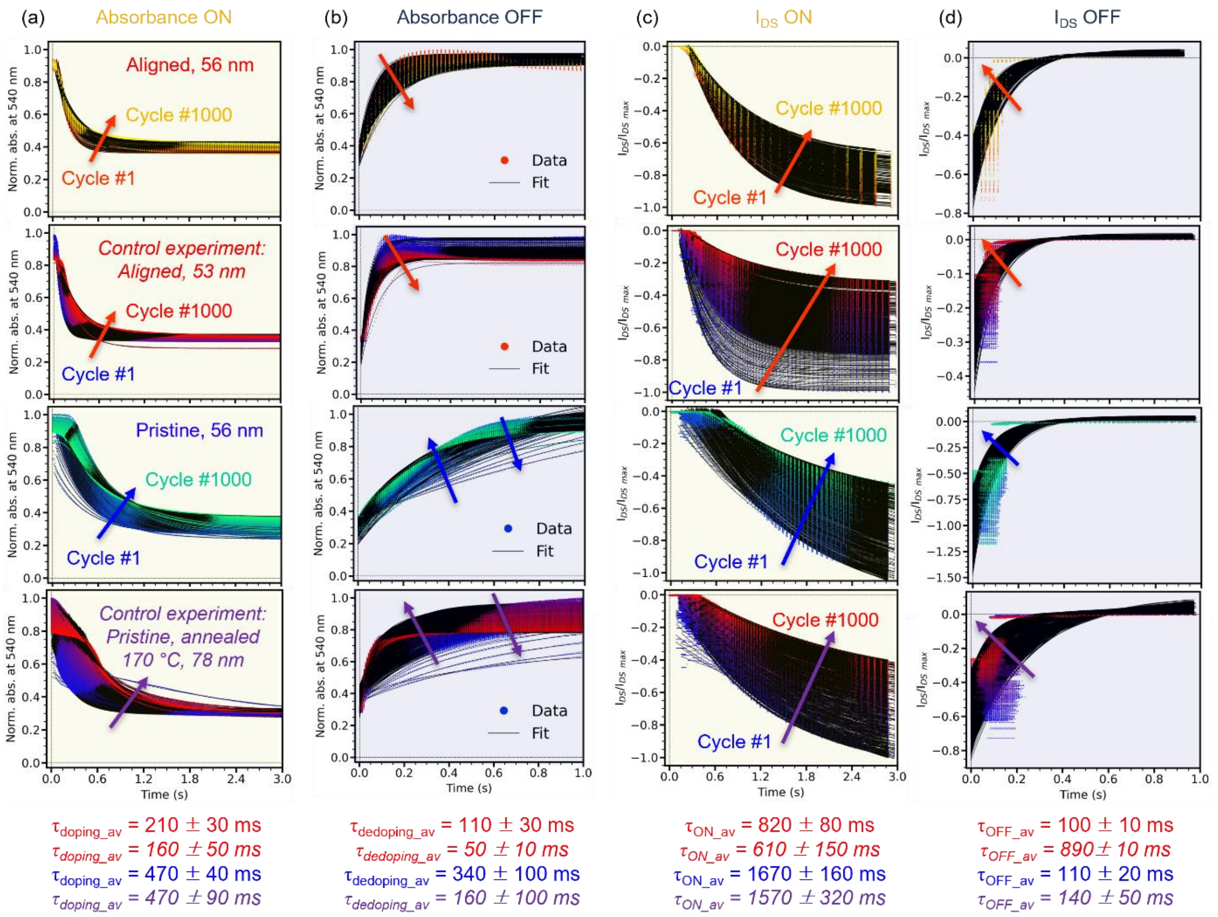

Figure S27. Control experiments and fits of time-resolved OECT response over 1000 ON and 1000 OFF cycles. Evolution of (a-b) the normalized absorbance at 540 nm (~neutral band, wavelength sampling) and (c-d) resulting I_{DS} current upon (a-c) ON and (b-d) OFF switching. Colored scattered points = data. Black lines = exponential fits. (From top to bottom) Aligned (56 nm), control aligned (53 nm), pristine (56 nm), control pristine but annealed at 170 °C (78 nm) **PBTTT-^{8}O** OECTs. The time constants, averaged over 1000 cycles, extracted from control experiments are presented in *italic*.

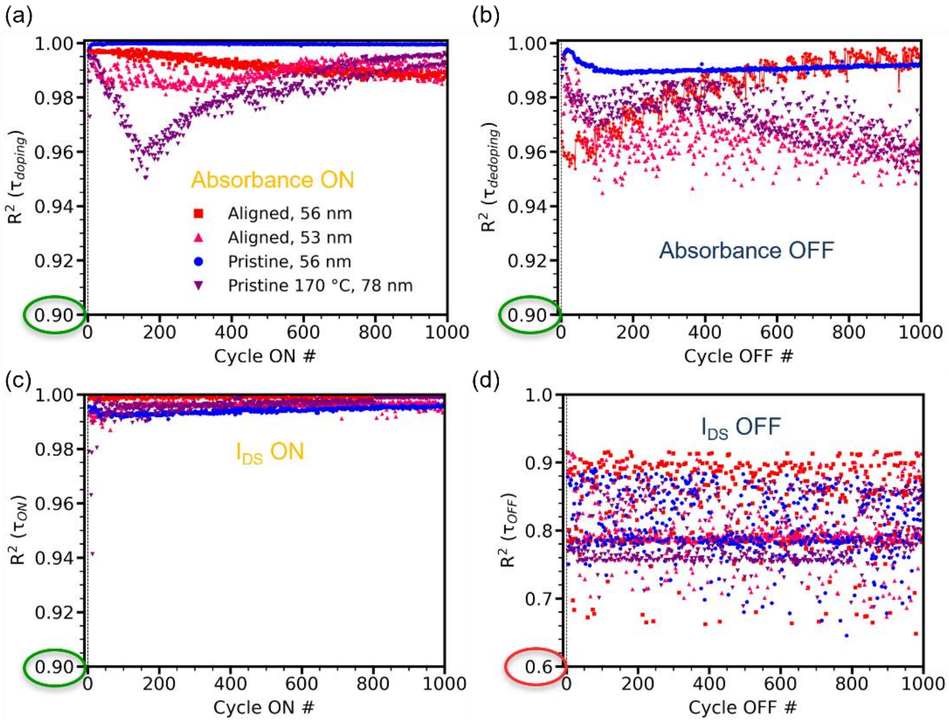

Figure S28. Cycle-after-cycle evolution of the R^2 of all fits of Figure S27. The panels a, b and c show the appropriate fit quality ($R^2 > 0.95$) to extract τ_{doping}, $\tau_{dedoping}$, τ_{ON}, t_{p_doping} and t_{p_ON}. However, panel d shows poor fit quality ($0.65 < R^2 < 0.95$) to extract τ_{OFF} due to slow time resolution. τ_{OFF} is hence accurately extracted from **Figure S30** below.

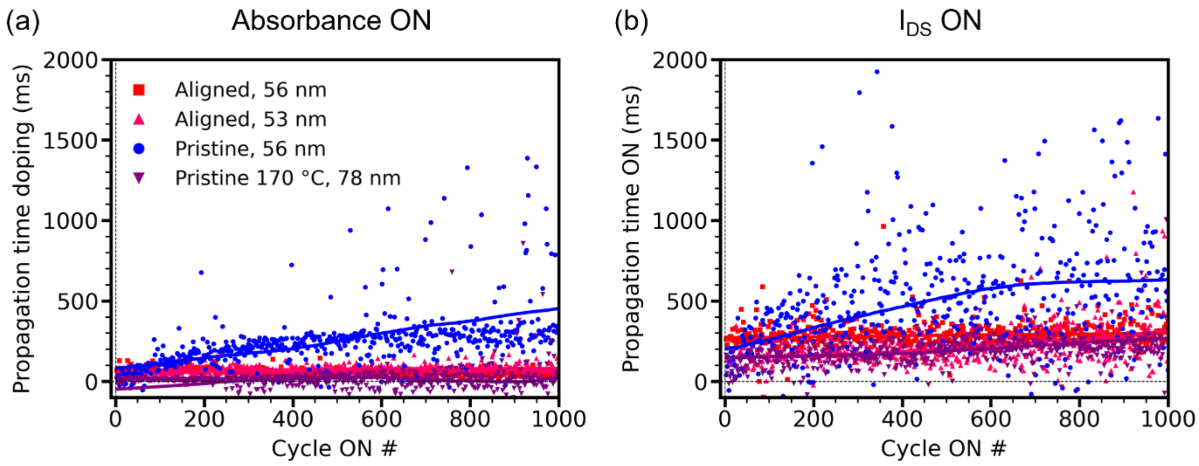

Figure S29. Cycle-after-cycle evolution of the optical doping front propagation time (t_{p_doping}) and I_{DS} ON "propagation time" (t_{p_ON}) of (red and pink) aligned, (blue) pristine and (purple) pristine but annealed OECTs over 1000 ON and OFF cycles. Scattered points = data. Solid line = Smoothed data using a Savitzky–Golay filter in Python (window length of 1000, polynomial order of 5).

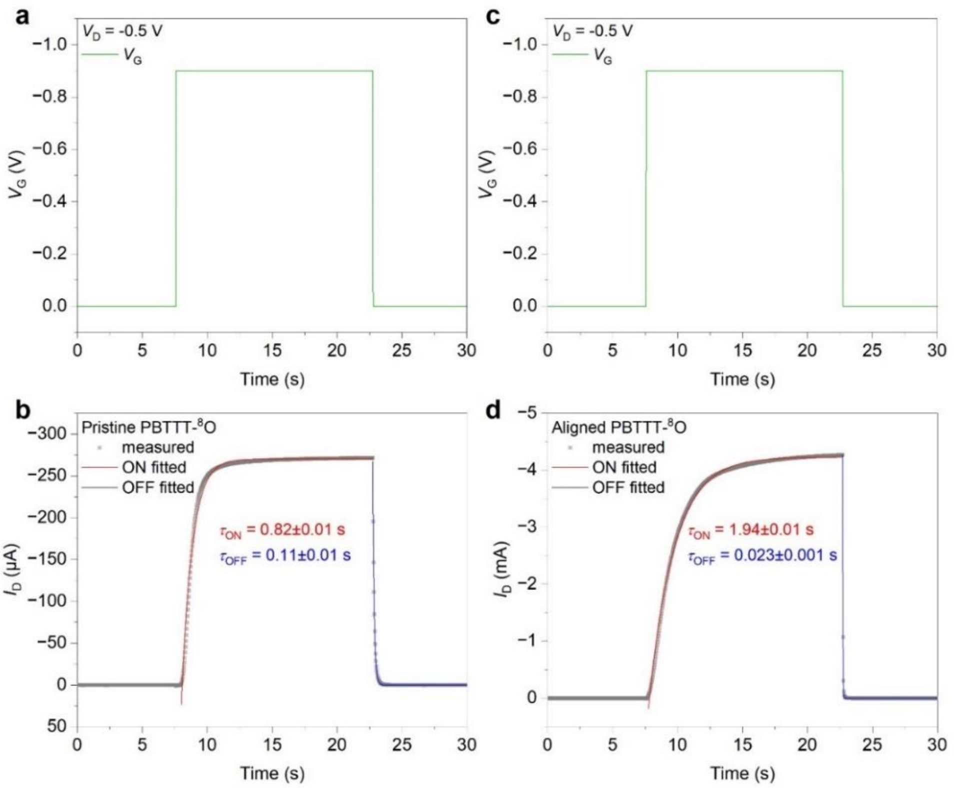

Figure S30. Time-resolved I_{DS} response of µm-long OECTs (W/L = 2000 µm/500 µm) of (a-b) pristine, (c-d) aligned **PBTTT-^{8}O** OECTs (d = 42 nm and 40 nm, respectively). V_{DS} was biased to -0.5 V, while V_{GS} was applied as a square wave pulse with an amplitude of -0.9 V and a duration of 15 s. The OECT $I_{DS,\,max}$ was 280 µA for pristine and increased to 4.5 mA for aligned **PBTTT-^{8}O (16× enhancement)**. The τ_{ON} increased from 820 ms for pristine to 1940 ms for aligned **PBTTT-^{8}O**. However, τ_{OFF} decreased from 110 ms for pristine to 23 ms for aligned **PBTTT-^{8}O**. In other words, aligned OECTs switches ON **2.4× slower** than the pristine ones but switches OFF **4.8× faster**. Note that the slower switching ON is in contradiction with the values extracted from the pulsing stability experiments done on 2-mm-channel OECTs and confirmed on 4 samples (**Figure S27**). Uneven degradations of the pristine and aligned samples during shipment to Sweden under ambient conditions and/or geometrical effects of the channel length (L = 2000 *vs.* 500 µm) may partly explain this difference.

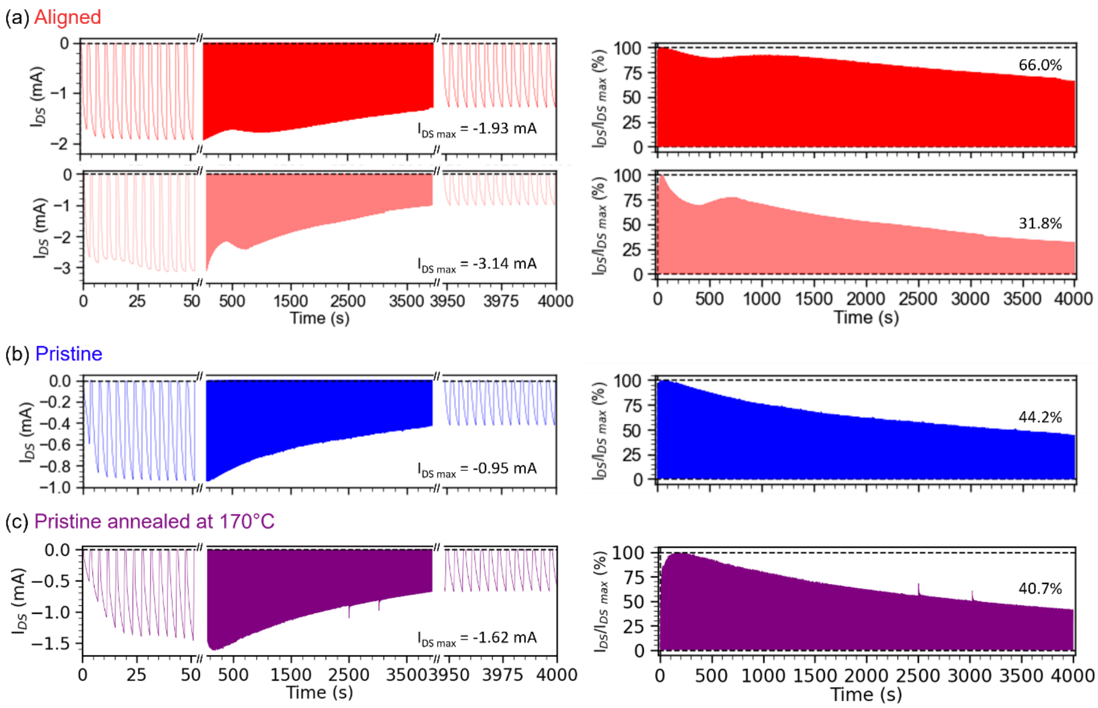

Figure S31. Pulsing stability of I_{DS} current over 1000 ON and 1000 OFF cycles of (a) aligned, (b) pristine, (c) 170 °C-annealed pristine **PBTTT-^{8}O** OECTs (Cr/Au SD electrodes, dwell ON/OFF = 3/1 s, V_{GS} = -0.8/+0.5V, V_{DS} = -0.5/0V, stress duration = 1 h 6 min). Temporal evolution of (left) absolute I_{DS} and (right) I_{DS} normalized by $I_{DS,max}$, the maximum I_{DS} reached for all cycles.

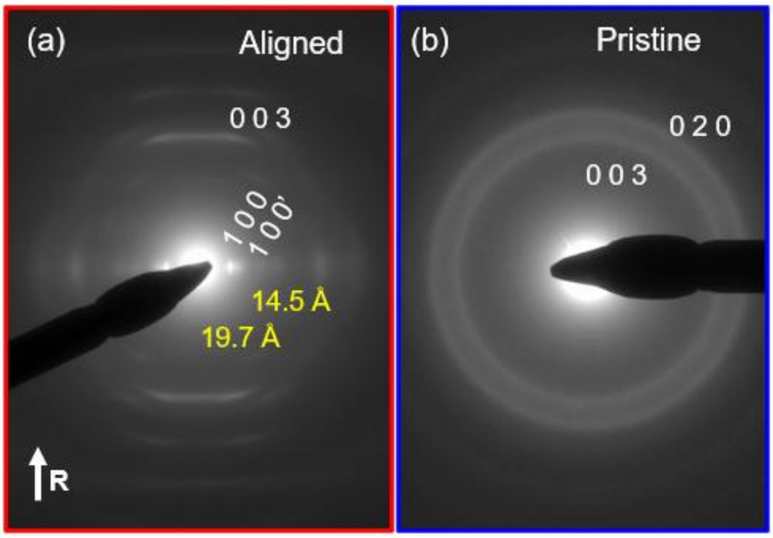

Figure S32. Electron diffraction patterns of (a) aligned and (b) pristine, non-rubbed, PBTTT-^{8}O films. The white arrow indicates the direction of rubbing R at 170 °C. The most intense reflections are indexed. Corresponding reticular distances are noted in yellow. Please note that rub-aligned PBTTT-^{8}O films are polymorphic with coexistence of two phases with characteristic 1 0 0 and 1 0 0' reflections at 19.7 Å and 14.5 Å, respectively.

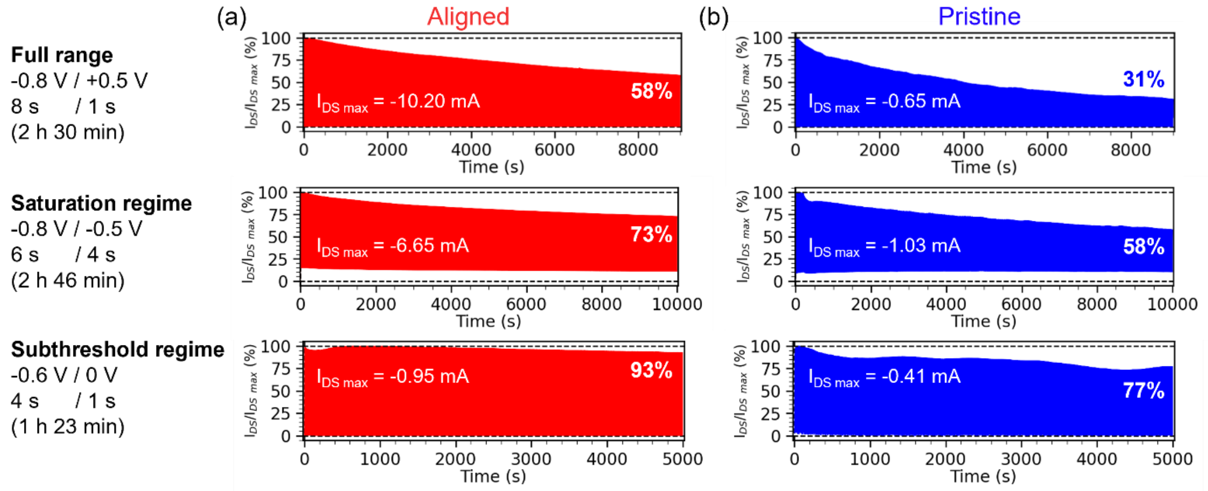

Figure S33. Pulsing stability of I_{DS} current, normalized by $I_{DS,max}$, over 1000 ON and 1000 OFF cycles of (a) aligned, (b) pristine **PBTTT-^{8}O** OECTs in (from top to bottom) the full operating range, the saturation regime and the subthreshold regime (**Ti**/Au SD electrodes, degassed 0.1 M KPF_6/H_2O electrolyte, V_{DS} = -0.5, d = 45–57 nm, W = 2.7–3.1 mm, L = 2.0 mm, $\frac{Wd}{L}$ = 68–81 nm, dwell adapted for each regime tested to reach steady-state for the aligned OECTs, stress duration indicated in parentheses). Inset: I_{DS} retention after 1000 ON/OFF cycles.

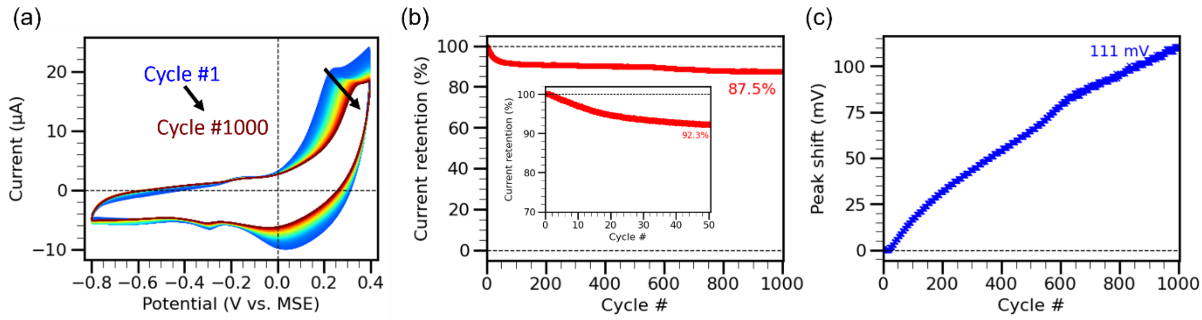

Figure S34. (a) Cyclic voltammetry of PBTTT-^{8}O in 0.1 M KPF_6/H_2O electrolyte over 1000 cycles at a scan rate of 100 mV s^{-1} (about 6 h 40 min). (b) Evolution of the current of the oxidation peak over cycles, normalized by the maximum current of the first cycle, showing a current retention of **88%** from 20.6 µA to 18.0 µA. Inset: Zoom on the first 50 cycles, highlighting that, of the 12% lost over 1000 cycles, 8% are already lost on the 50 first cycles. (c) Evolution of the peak position over cycles, normalized by the position of the peak of the first cycle, showing a shift of 111 mV from 0.252 V to 0.363 V, assigned to an increase of contact resistance between the working electrode and the polymer film.

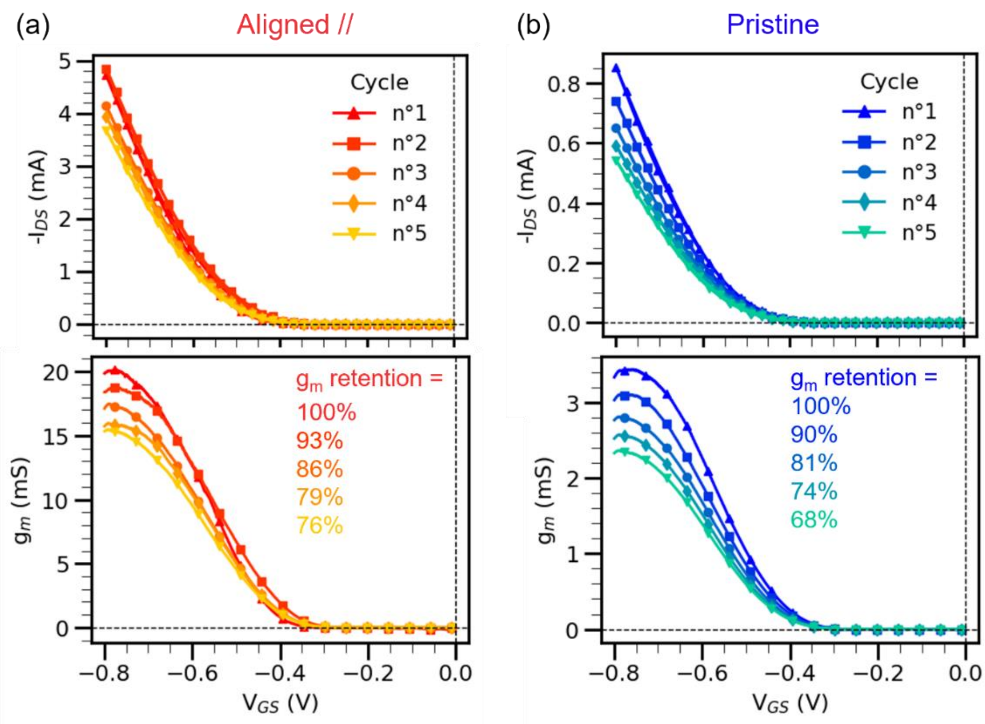

Figure S35. Stability over five consecutive transfer characteristics of (a) aligned (d* = 54 nm, W = 2.5 mm, L = 2.0 mm, OP = 0.75) and (b) pristine (d = 56 nm, W = 3.1, mm, L = 2.0 mm) **PBTTT-^{8}O** OECTs in degassed 0.1 M KPF_6/H_2O electrolyte (**Ti**/Au SD electrodes, V_{DS} = -0.5 V, V_{GS} swept from 0 V to -0.8 V back to 0 V at a scan rate of 1 mV s^{-1}, stress duration = 2 h 13 min). (top) Transfer curves. (bottom) Corresponding transconductance curves. We observe an **76%** retention of the transconductance (g_m) after five consecutive transfer characteristics for **aligned** OECTs *vs.* **68%** retention for **pristine** OECTs. The values normalized by the channel dimensions are reported in **Table S7**. Note that limiting the range of V_{GS} sweep would likely increase the stability over transfer cycles, as shown for the pulsing stability in the subthreshold regime only.[6]

Supplementary Tables

Table S1. Literature summary of the performance of p-type OMIECs in OECTs.

Polymer	g_mL/Wd (S cm^{-1})	*μC** (F cm^{-1} V^{-1} s^{-1})	*C** [a-b-c] (F cm^{-3})	*μ* [d-e-f] (cm^2 V^{-1} s^{-1})	V_{Th} (V)	Year	Ref
Aligned **PBTTT-^{8}O**	2 580 ± 1 220†	10 660 ± 910	560 ± 70[bi]	19 ± 4.0[d]<br>15 ± 9.8[e]<br>2.0[f]	-0.32 ± 0.04[g]	2025	This work
Pristine **PBTTT-^{8}O**	430 ± 70†	960 ± 30	390 ± 40[bi]	2.5 ± 0.3[d]<br>3.3 ± 0.8[e]<br>0.11[f]	-0.32 ± 0.06[g]	2025	This work
P(g2T-TT), Mn-selected + Pd-purified	146 ± 0.6*	2 008 ± 130	308 ± 20[ai]	6.53 ± 0.07[d]	0.07 ± 0.003[g]	2022	[7]
P(g2T-TT)	238 ± 21*†	458 ± 57	/	/	0.33 ± 0.03	2019	[8]
	123 ± 16*	299.42 ± 5.15	171.4 ±8.4[ai]	1.82 ± 0.09[e]	0.00 ± 0.01[g]	2022	[9]
	135*	227 ± 107	241 ± 94[aii]	0.94 ± 0.25[f]	0.00	2016	[10]
	42*	/	270 ± 20[aii]	0.33[f]	/	2019	[11]
PgBTTT	687 ± 91	1 983 ± 169	/	/	-0.25 ± 0.02	2024	[12]
	194 ± 2*	502 ± 18	164 ± 7[aiv]<br>141[bii]	3.44 ± 0.13[e]	-0.24[g]	2021	[13]
PE_2-OE_4	453 ± 70	830 ± 37	207 ± 19[bi]	4.0 ± 0.6[d]	0.24 ± 0.03[g]	2024	[14]
Aligned P3HT	*g_m not reported in relation to W, d, L*	717 ± 157†	250[bii]	2.8[d]	-0.43	2024	[15]
Pristine P3HT		93 ± 12†	150[bii]	0.9[d]	-0.50	2024	[15]
		10.4 ± 3.1†	/	/	/	2019	[16]
Porous P3HT	341 ± 33†	349 ± 43			−0.72 ± 0.05	2021	[17]
P(3g2T-T) + CB + BCF	130 ± 6.8	556.3 ± 43.7	166[aii]	3.35 ± 0.26[e]	-0.06[g]	2022	[18]
P(g2T2-g4T2)	144 ± 36*	522	187 ± 8[ai]	1.72 ± 0.31[e]	+0.02[g]	2020	[19]
PEDOT:PSS + EG	100 ± 10	406 ± 39	109 ± 5[ai]	2.5 ± 0.9[e]	+0.6[g]	2023	[20]
	460*	75 ± 51	39 ± 3[c]	1.9 ± 1.3[f]	/	2016	[21-22]
P(gDPP-T2)	70 ± 8*	342 ± 35	196[ai]	1.55 ± 0.17[e]	-0.52	2021	[23]

Aligned P(3g2T-T)	233	330 ± 20	175 ± 5^{aiii}	0.93^{f}	/	2022	[24]
Pristine P(3g2T-T)	48 ± 3*	135 ± 9	211 ± 18^{aii}	0.16 ± 0.01^{f}	-0.18 ± 0.01^{g}	2020	[25]
	77*	167 ± 65	220 ± 30^{ai}	0.28 ± 0.1^{f}	0.00	2016	[22, 26]
PProDOT-DPP	149*†	310	330^{bii}	0.94^{d}	+0.1^{g}	2021	[27]
ProDOT(g4)-DMP	76 ± 7	200 ± 8	213 ± 11^{biii}	0.94 ± 0.09^{d}	-0.36 ± 0.01^{g}	2023	[28]
P(bgDPP-MeOT2)	83 ± 3*	195 ± 21	120 ± 2.4^{ai}	1.63 ± 0.14^{d}	-0.33^{g}	2021	[29]
P3MEEMT	54*†	96.7 ± 10.2	294^{aii}	0.19 ± 0.03^{d}	/	2019	[16]
	/	/	259^{aii}	0.37^{d}	-0.42 ± 0.01^{g}	2022	[1]
PDPP[T]2(TEG)3-MEET	/	45	338^{ai}	0.133^{d}	-0.36^{g}	2021	[30]
P3APPT	11*†	41.3 ± 2.8	152 ± 21.1^{aiii}	0.27 ± 0.06^{d}	-0.27 ± 0.01^{g}	2022	[1]
PB2T-TEG	31*†	25 ± 4	195 ± 40^{ai}	0.13 ± 0.05^{d}	/	2020	[31]
P3MEEET	2.04*†	11.5 ± 1.4	242 ± 17^{ai}	0.05^{e}	-0.57^{g}	2020	[32]
p(gPyDPP-MeOT2)	19.5 ± 2.5	1.8^{a}	60^{ai}	0.030 ± 0.007^{e}	-0.5	2020	[33]
$PTHS^{-}$ TMA^{+}-co-P3HT 2	7.049*	1.7	100 ± 7^{ai}	0.017 ± 0.0036^{e}	-0.15^{g}	2019	[34]
P(gBDT-g2T)	4.3*	1.4 ± 0.6	77 ± 23^{ai}	0.018 ± 0.006^{e}	-0.3	2016	[22, 26]
PTHS + EG	1.3 ± 0.1*	0.16 ± 0.15	124 ± 38^{ai}	0.0013 ± 0.0011^{d}	/	2014	[22, 35]

^{a} *C** extracted by electrochemical impedance spectroscopy (EIS) $^{(ai)}$ at the voltage inducing the maximum g_m, $^{(aii)}$ at the voltage inducing the maximum *C**, $^{(aiii)}$ at a given voltage above V_{Th} but below maximum g_m, $^{(aiv)}$ averaged over voltages above the threshold voltage. ^{b} *C** extracted by chronoamperometry or coulometry $^{(bi)}$ from a linear fit of the injected charge as a function of voltages above V_{Th}, $^{(bii)}$ at a given voltage above V_{Th}, $^{(biii)}$ at the voltage inducing the maximum *C** in accordance with EIS. ^{c} *C** extracted from $\mu C^*/\mu$. ^{d} Hole mobility μ inferred from $\mu C^*/C^*$. ^{e} μ extracted from the slope of $\sqrt{I_{DS}^{Sat}}$ vs. V_{GS} plot in the saturation regime and *C**. ^{f} μ measured independently using the impedance matching method. Note that regardless of the method used to extract μ (^{d}, ^{e} or ^{f}) the μ found for aligned **PBTTT-^{8}O** OECTs systematically outperform previous μ reported with the same method. ^{g} Determined as the x-axis intercept of $\sqrt{I_{DS}^{Sat}}$ vs. V_{GS} plot based on the linear extrapolation method, otherwise extraction method not

specified. [†]OECTs operating in an aqueous electrolyte, but different than $NaCl/H_2O$ electrolyte. *Calculated from $g_m \pm \Delta g_m$, $d \pm \Delta d$, L, and W reported in the text or estimated from the figures of the references.

Table S2. Results of the transfer characteristics in the saturation regime (Figure S12). Devices used to determine the averaged geometry-normalized transconductance $g_m L/Wd$ and μC^* products. The error given is one standard deviation. The SD overlap values in percent correspond to the film area covering the source and drain contacts compared to the channel area. A SD contact overlap of >60% was chosen to reduce carrier injection barrier. The threshold voltage V_{Th} is defined as the intercept of the linear regression of $\sqrt{I_{DS}^{Sat}}$ vs. V_{GS} plot and the abscissa (**Figure S13**). V_{GS} is here the gate-source voltage inducing the maximum transconductance g_{m_max}. Note that the normalized transconductance $g_m L/Wd$ is independent of any model which facilitates OECT-OECT comparison with the literature.

OP (if aligned)	d (nm)	W (mm)	L (mm)	Wd/L (nm)	SD overlap (%)	V_{Th} (V)	V_{GS} (V)	g_m (mS)	$g_m L/Wd$ (S cm^{-1})	I_{ON}/I_{OFF}
Aligned 0.67	81*	2.9	2.0	117	66	-0.41	-0.70	43.68	3719	7 x 10^6
0.66	80*	2.6	2.0	104	122	-0.28	-0.74	47.40	4558	1 x 10^4
0.79	42*	2.4	2.0	50.4	94	-0.34	-0.70	12.88	2556	1 x 10^4
0.69	41*	1.9	2.0	39.0	111	-0.34	-0.67	12.27	3150	2 x 10^6
0.74	24*	1.6	2.0	19.2	84	-0.29	-0.73	3.46	1802	1 x 10^4
0.74	32*	1.2	2.0	19.2	115	-0.31	-0.68	2.44	1271	2 x 10^5
0.79	21*	2.6	2.0	27.3	97	-0.28	-0.64	2.73	1000	6 x 10^4
Pristine	79	4.7	0.5	743	193	-0.27	-0.75	30.14 bimodal	405	8 x 10^4
	66	5.0	0.5	660	177	-0.26	-0.75	32.02 bimodal	485	2 x 10^5
	56	4.9	0.5	549	210	-0.26	-0.69	25.00 bimodal	456	5 x 10^4
	44	5.0	0.5	440	218	-0.28	-076	22.01 bimodal	500	2 x 10^5
	46	2.6	1.0	120	248	-0.38	-0.72	4.31	360	1 x 10^5
	19	2.8	0.5	106	152	-0.27	-0.67	3.63	341	3 x 10^4
	79	2.2	2.0	86.9	88	-0.30	-0.75	3.83	441	1 x 10^5
	57	2.9	2.0	82.7	80	-0.41	-0.71	3.34	404	2 x 10^6
	64	1.9	2.0	60.8	95	-0.33	-0.70	3.40	559	2 x 10^6
	60	1.0	2.0	30.0	85	-0.39	-0.70	1.03	343	3 x 10^5

Table S3. Results of the control transfer characteristics (Figure S15).

	d (nm)	W (mm)	L (mm)	Wd/L (nm)	SD overlap (%)	V_{Th} (V)	V_{GS} (V)	g_m (mS)	g_mL/Wd (S cm^{-1})	I_{ON}/I_{OFF}
Aligned, but ⊥	67*	1.9	2.0	63.7	60	-0.28	-0.72	0.34	53.4	1 x 10^3
Doctor-blade but not rubbed	162*	3.0	2.0	243	62	-0.38	-0.82	7.77	320	1 x 10^5
	163*	2.6	2.0	213	79	-0.34	-0.81	8.34	393	1 x 10^5
	164*	2.3	2.0	189	85	-0.28	-0.68	7.59	402	4 x 10^4
Pristine 170°C	78	3.7	0.5	577	130	-0.27	-0.64	17.41	302	2 x 10^5
	42	4.3	0.5	361	215	-0.27	-0.74	12.77	353	1 x 10^5

Table S4. Results of the chronoamperometry measurements. '*C** at +0.7 V' extracted from **Figure S18**c, resulting in average $C^*_{0.7V\text{-aligned}}$ = 373 ± 43 F cm^{-3} and $C^*_{0.7V\text{-pristine}}$ = 283 ± 26 F cm^{-3} for aligned and pristine **PBTTT-^{8}O**, respectively. The C* used to estimate μ below is the '*C** from fit' extracted from **Figure S18**d. Averaged over 4 and 3 independent samples for aligned and pristine **PBTTT-^{8}O** the values are $\boldsymbol{C^*_{aligned}}$ **= 560 ± 70 F cm^{-3}** and $\boldsymbol{C^*_{pristine}}$ **= 390 ± 40 F cm^{-3}**, respectively. The error given is the combination of (i) the standard deviation on each initial value with (ii) the combined error of each initial standard deviation. We assume the errors to be independent and propagate them using the quadrature method (*i.e.*, taking the square root of the sum of the squares of errors (i) and (ii)). Total volume = dLW(1+ SD_overlap/100).

OP (if aligned)	d (nm)	W (mm)	L (mm)	SD overlap (%)	Total volume (10^{-4} mm^3)	*C** at +0.7 V (F cm^{-3})	*C** from fit (F cm^{-3})
Aligned 0.74	32*	1.2	2.0	115	1.65	385	**450 ± 44**
0.78	47*	2.3	2.0	81	3.91	302	**618 ± 45**
0.79	61*	2.4	2.0	63	4.77	419	**558 ± 27**
0.80	56*	2.2	2.0	95	4.80	387	**609 ± 48**
Pristine	57	1.9	4.5	42	6.92	310	**445 ± 37**
	58	4.45	2.0	88	9.70	290	**384 ± 29**
Doctor-blade but not rubbed	163*	2.6	2.0	79	15.2	248	**350 ± 23**

Table S5. Fitting parameters from the Drude-Smith model of the complex THz conductivity spectra (Figure S26).

PBTTT-^{8}O	d (nm)	DR (OP)	N_{DS} (cm^{-3})	τ (fs)	c_1	μ_{eff} (cm^2 V^{-1} s^{-1})	σ_{THz} (S cm^{-1})	$\frac{\mu_{eff}\, g_m L}{\sigma_{THz} W d}$ (cm^2 V^{-1} s^{-1})^a
Aligned	101*	13.38 (0.81)	8.45E+20	28	-0.78	6.5 (1.8×)	1022 (2.0×)	16 (5.2×)
Pristine	123	1	6.93E+20	24	-0.85	3.7	510	3.1

^{a} Note that the σ_{THz} scale in the same range than the geometry-normalized transconductance g_mL/Wd. For **aligned** OECTs, g_mL/Wd = 2 580 ± 1 216 S cm^{-1} was found, being 2.5× superior to σ_{THz}. For **pristine** OECTs, g_mL/Wd = 430 ± 69 S cm^{-1} was found, being 0.84× inferior to σ_{THz}. If we account for this shift between THz measurements and OECT measurements, one can estimate a corrected 'macroscale' mobility $\mu_{macro} = \frac{\mu_{eff}\, g_m L}{\sigma_{THz} W d}$ found to be 16 cm^2 V^{-1} s^{-1} and 3.1 cm^2 V^{-1} s^{-1} for aligned and pristine OECTs, respectively. This calculation allows to exploit the reliable long mm-range OECT measurement of the transconductance, independent of any model, and effective short nm-range mobility to estimate a 'corrected macroscale mobility', confirming a significant **5.2× enhancement** of the carrier transport properties upon polymer alignment.

Table S6. Geometric factors and normalized I_{DS} current retention over 1000 ON and 1000 OFF cycles (Figure S31). When the OECT was switched OFF before reaching steady-state, a higher stability is found for aligned OECTs (66% vs. 44%). The stability decreases if steady-state - associated to higher extent of doping - is reached, in accordance with recent literature from Keene *et al.*.[6]

PBTTT-^{8}O	d (nm)	W (mm)	L (mm)	Wd/L (nm)	SD overlap (%)	$I_{DS,max}$ (mA)	I_{DS} retention over 1000 cycles (%)
Aligned	56*	2.2	2.0	61.6	95	-1.93	**66.0**
	53*	2.3	2.0	61.0	73	-3.14	**31.8**
Pristine	56	2.4	2.0	67.2	98	-0.95	**44.2**
Pristine 170°C	78	2.5	2.0	97.5	60	-1.62	**40.7**

Table S7. Results of the stability over five consecutive transfer characteristics (Figure S35).

Cycle number	Stress duration (min)	V_{Th} (V)	V_{GS} (V)	g_m (mS)	g_mL/Wd (S cm^{-1})
Aligned 1	26.7	-0.38	-0.77	20.17	2990
2	26.7	-0.35	-0.77	18.79	2780
3	26.7	-0.36	-0.79	17.48	2590
4	26.7	-0.36	-0.79	15.98	2370
5	26.7	-0.36	-0.79	15.49	2290
Pristine 1	26.7	-0.36	-0.76	3.45	480
2	26.7	-0.36	-0.77	3.12	440
3	26.7	-0.37	-0.79	2.82	400
4	26.7	-0.37	-0.79	2.58	360
5	26.7	-0.37	-0.79	2.37	330

Supplementary Notes

Note S1. OECT fabrication.

OECTs with mm-long channels (L = 2000 μm): The fabrication of both pristine and aligned OECTs is detailed in the **Experimental Section** of the main text. Cr/Au source-drain electrodes are replaced by Ti/Au electrodes for transfer and pulsing stability experiments to avoid

OECTs with μm-long channels (L = 10 μm or 500 μm): OECTs with two different geometries were employed (*W/L* = 100 μm/10 μm and *W/L* = 2000 μm/500 μm). The ones with *W/L* = 100 μm/10 μm were fabricated following a previously reported procedure.[36] The microscope glass slides underwent thorough cleaning, involving successive sonication in acetone, deionized water, and isopropanol, followed by drying with nitrogen. The source/drain electrodes were thermally deposited with 5 nm of Cr and 50 nm of Au and then patterned using photolithography followed by wet etching. A first layer of parylene C (2 μm) was coated along with a drop of 3-(trimethoxysilyl) propyl methacrylate (A-174 Silane), serving as an insulating layer between the metal electrode and the electrolyte. Subsequently, a dilution of industrial surfactant (2% Micro-90) was spin-coated before depositing the second layer (sacrificial layer) of parylene C (2 μm). In order to shield the parylene C layers from the following plasma reactive ion etching step (150 W, O_2 = 500 sccm, CF_4 = 1000 sccm, 380 s), a thick positive photoresist (5 μm, AZ 10XT 520CP) was applied via spin-coating atop the parylene C layers. A second photolithographic patterning step was conducted, followed by an application of AZ developer to define the contact pads and the OECT channels. The following plasma reactive ion etching step was applied to uniformly remove the organic layers, including the photoresist and parylene C, until the contact pads and the OECT channel area were exposed to the air, while the remainder of the device retained its coverage with two layers of parylene C. The channel between the source and drain was patterned to have dimensions of *L* = 10 μm and W = 100 μm. The deposition of pristine and aligned **PBTTT-^{8}O** on these substrates is identical to the mm-long OECTs. The sacrificial layer of parylene C was peeled off, removing the **PBTTT-^{8}O** film on top of it and leaving the polymer in the patterned area, but comprised the OECT channel and the electrode pads. Images of aligned **PBTTT-^{8}O** OECTs with a 10-μm-long channel are shown in **Figure S5**. The OECTs with *W/L* = 2000 μm/500 μm were fabricated by sequentially thermally depositing 5 nm of Cr and 50 nm of Au onto cleaned microscope glass slides using a shadow mask and after **PBTTT-^{8}O** deposition, the channel area was manually patterned using swabs. The OECTs were characterized with a Keithley 4200A-SCS on an independent setup.

Note S2. Thickness determination of aligned films.

An accurate determination of the film thickness is paramount as (i) the μC^* product scales linearly with the thickness d, and (ii) the (de)doping kinetics depend on the thickness the charge carriers/ions should diffuse through. As a result, a dry thickness-absorbance calibration curve is reported to validate the thickness determination. Different thicknesses were obtained by diluting the stock o-dichlorobenzene solution at 12 mg mL^{-1} to 10 and 5 mg mL^{-1}. The Vis-NIR absorbance spectra of these dry films were acquired in inert atmosphere using a halogen lamp (HL-2000), a Vis-NIR spectrophotometer (Flame Vis-NIR), and an NIR spectrophotometer (Flame NIR) from Ocean Optics. The blank quartz-coated glass substrate was subtracted from the transmission spectra to afford the absorbance spectra of the polymer only (**Figure S7a**). In the solid-state away from ambient species, dry **PBTTT-^{8}O** absorbance spectra exhibit a Gaussian-like profile centered at ~540 nm and a shoulder centered at ~580 nm. Similar bands were observed for PBTTT-C_{14} in solution and were respectively attributed to π→π* transition (0-1 transition) and to weak coupling of π→π* with C=C bond of the thiophene rings (0-0 vibronic transition).[37] We note a bathochromic shift (~20 nm) of the 0-1 band, and an hyperchromic shift of the 0-0 shoulder for thinner films casted from more dilute solutions (**Figure S7b**). We tentatively attribute these shifts to a higher degree of semi-crystallinity (more ordered microstructure) afforded by less entangled polymer chains in more dilute solutions. The "max absorbance" of the spectra at ~520-540 nm is considered for the calibration curve. The thicknesses were measured using a Bruker Contour GT-K optical profiler using light interferometry (green light, PSI mode) controlled by Vision 64 software. Each value used for the calibration curve is the average of 4-6 thickness measurements. The results are given in **Figure S7c**. The max absorbance shows a linear dependence on the film thickness in accordance with Beer-Lambert law. The high $R^2 > 0.98$ validates the accurate determination of the dry thicknesses. The slope of the thickness-absorbance calibration curve (= 94.8) extracted from pristine films is then used to estimate the dry thickness of aligned films of **PBTTT-^{8}O**. According to Beer-Lambert law, this slope is proportional to the product of absorbing species concentration times the extinction coefficient. The isotropic absorbance of aligned films is obtained by polarized Vis-NIR spectroscopy from $A_{iso} = \frac{A_{\parallel} + A_{\perp}}{2}$.[38] Assuming the concentration-extinction coefficient product similar between pristine and aligned films on average, the averaged thickness of the aligned film can be estimated from: $d_{av} = A_{iso} * 94.8$. The absorbance spectra obtained with an analyzer parallel (//) or perpendicular (⊥) to the rubbing direction are given in **Figure S6a-d** for some of the OECTs used for the determination

of the μC* product (**Figure S12a**). A linear background correction was applied to account for scattering.

Note S3. Steady-state Vis-NIR spectroelectrochemistry

All steady-state Vis-NIR spectroelectrochemistry experiments were performed on SD short-circuited OECTs in degassed 0.1 M KPF_6/H_2O electrolyte. Square-wave doping/dedoping voltage pulses were applied using a data acquisition system (USB-6211 from National Instrument Corp.) synchronized with the Vis-NIR spectrophotometers using LabVIEW program. The Vis-NIR absorbance spectra were acquired with a Flame UV-Vis spectrometer and a FlameNIR spectrometer (Ocean Optics) triggered by the data acquisition card. The incident white light was generated by a halogen light source (HL-2000, Ocean Optics). It was collimated through a glass slide (0.55 mm), 2 mm of the electrolyte, the transistor channel and the S151 substrate (1.1 mm). The dedoping voltage was kept constant at V_{OFF} = 0 V for 150 s. The doping voltages were increased stepwise from $V_{ON} \simeq$ 0.1 to 1.0 V (ΔV = +0.1 V), each held for 150 s. Simultaneously, the Vis-NIR absorbance spectra were recorded continuously with a time-resolution of about 5 ms. The blank quartz-coated glass substrate was subtracted from the transmission spectra to afford the absorbance spectra of the polymer only. The steady-state Vis-NIR voltage-dependent absorbance spectra were obtained by averaging the last 20 spectra of each voltage step. For pristine samples no analyzer was used (**Figure 1c** main text). For aligned samples, an analyzer placed after the samples was added and oriented parallel and perpendicular to the rubbing direction of **PBTTT-^{8}O** as illustrated in **Figure S9** (**Figure 1d-e** main text).

Note S4. Output characteristics, transfer characteristics and Bernards-Malliaras model.

All output characteristics were performed on pre-cycled OECTs in degassed 0.1 M KPF_6/H_2O electrolyte. Fixed V_{GS} voltage was applied using a data acquisition system (USB-6211 from National Instrument Corp.) and decreased stepwise from -0.3 V to -0.8 V (ΔV = -0.1 V). V_{DS} is swept using a Keithley 2400 SMU (Tektronix) from 0 V to -0.8 V at a scan rate of 2 mV s^{-1}. The resulting I_{DS} current was recorded with the Keithley 2400 SMU. Here the voltage sign follows the transistor convention where the source is grounded (*i.e.*, $V_{GS} < 0$ induces p-doping).

In the Bernards-Malliaras model, the drain-source I_{DS} current in the *linear regime* follows: $I_{DS} = \frac{Wd}{L}\mu C^* \cdot V_{DS}\left(V_{Th} - V_{GS} + \frac{V_{DS}}{2}\right)$ according to literature[39] (**Figure S10**, orange region).

And, the I_{DS} current in the *saturation regime* (**Figure S10**, green region) is described by: [39]

$$I_{DS}^{Sat} = \frac{Wd}{2L} \mu C^{*} (V_{Th} - V_{GS})^{2} \qquad \text{Equation (1)}$$

With W, d, and L, the channel width, depth (*i.e.*, average thickness), and length respectively, μ the charge carrier mobility, C^* the volumetric capacitance, V_{Th} the threshold voltage, and V_{GS} the gate-source voltage affording maximum transconductance.

Transfer characteristics were performed to quantify the geometry-normalized transconductance (g_mL/Wd) and the μC^* product. All experiments were carried out on OECTs in degassed 0.1 M KPF_6/H_2O electrolyte. The I_{DS} enhancement achieved for mm-long channel OECTs was confirmed by testing an OECT with a 500-µm-long channel (**Figure S17**). Prior to the experiment, the electrolyte was degassed by nitrogen bubbling for 10 min. The electrolyte was injected in the closed cell and the device held at +0.5 V until steady dedoped state is reached. V_{DS} was applied using a Keithley 2400 SMU (Tektronix) and fixed at -0.5 V prior applying V_{GS}. A negative V_{DS} allows to extract electron holes, as it increases the conduction band level ($\vec{\mathcal{E}} = -\overrightarrow{grad}\, V$). Then, V_{GS} was applied using a data acquisition system (USB-6211 from National Instrument Corp.) and swept back and forth from -0.1 V to -0.9 V back to -0.1 V at a scan rate of 1 or 2 mV s^{-1}. The resulting I_{DS} current was recorded with the same Keithley 2400 SMU. The transfer curves exhibit low hysteresis, which confirms that the selected scan rate is slow enough for the transistors to operate in a steady state (no limitation by ion diffusion, charge injection/extraction, electrolyte polarization, etc.).[40] Low hysteresis is favorable for most applications (except neuromorphics for instance) as it indicates limited charge trapping over a doping/dedoping cycle.[41] For these experiments, the voltage sign is based on the transistor convention where the source is grounded (*i.e.*, $V_{GS} < 0$ induces p-doping).

The apparent *"μC*"* is extracted for comparison purposes as commonly done in the literature from equation (2) below[3], despite recent criticism[4]. Note that the above-mentioned equation (1) is valid only if the transistor operates in the saturation regime. In this regime, the threshold voltage V_{Th} is typically estimated from the x-intercept of the $\sqrt{I_{DS}^{Sat}}$ vs V_{GS} plot using the linear extrapolation method (**Figure S13**). This method is chosen for comparison purpose with the literature (**Table S1**) but the authors would like to stress that other methods based on the second derivative of I_{DS} could afford more robust estimation of V_{Th}, especially when comparing OECTs with short and long channels.[42] V_{GS} is typically estimated from the voltage required

to reach maximum transconductance. To facilitate comparison with the literature, we report the normalized transconductance (g_mL/Wd) equivalent to an electrical conductivity. The authors argue for the use of g_mL/Wd rather than g_m/d, seen in literature, so that the resulting conductivity values are independent of the channel dimensions of the transistor. The average normalized transconductance found for aligned and pristine **PBTTT-⁸O** doped with aqueous KPF_6 are 2 580 ± 1 216 S cm^{-1} and 430 ± 69 S cm^{-1}, respectively (**Table 1**). Note that the relatively large standard deviation observed for aligned OECTs comes from lower conductivities for thinner films despite higher 3D order parameters (**Table S2**, **Figure S6**). An oxygen reduction reaction (ORR)-mediated degradation could explain this observation as diffusion of oxidative species scales with thickness.[43]

In the Bernards-Malliars model, the transconductance (g_m) is defined as the first derivative of equation (1):

$$g_m = \frac{dI_{DS}^{Sat}}{dV_{GS}} = \frac{Wd}{L}\mu C^* \cdot (V_{Th} - V_{GS}) \qquad \text{Equation (2)}$$

According to equation (2), the evolution of g_m scales linearly with the channel dimensions and bias factors, giving μC^* as the slope of **Figure S12**a.[44] The linear regressions over 7-10 devices show high R^2 values > 0.91, confirming high reproducibility in OECT manufacture and fabrication. High R^2 indicates a mathematical linear correlation between g_m and the geometric and bias factors[45], and therefore a high confidence in the extracted slope values reported in **Table 1**. Importantly, note that if a linear correlation is clear, the nature of the slope cannot be defined and may be a term more complex than the product of μ and C^*.[4]

Note S5. Determination of *C*.*

Two distinct methods were employed to determine the volumetric capacitance C^*: (i) chronoamperometry, and (ii) electrochemical impedance spectroscopy (EIS).

First, chronoamperometry measurements coupled with Vis-NIR spectroelectrochemistry were performed on mm-channel SD short-circuited OECTs (V_{DS} = 0) in degassed 0.1 M KPF_6/H_2O electrolyte to estimate the volumetric capacitance C^*. It consists of recording the temporal evolution of the gate-source I_{GS} current upon application of square pulses of V_{GS}. V_{GS} is increased from +0.1 V to +0.8 V by steps of 0.1 V. Each step is held for 150 s (dwell time) while recording the I_{GS} current transients (**Figure S18**b). At the end of each V_{GS} step, the device

is briefly exposed to light to record a steady-state Vis-NIR absorbance spectrum of the channel (**Figure S18**a). The light is blocked by a shutter before applying the next V_{GS} step to avoid the contribution of potential photo-generated charges in the I_{GS} current. By integrating the current over time, we estimate the total injected charge (**Figure S18**c). Then, *C** is found from the slope of the carrier density as a function of voltages above the threshold voltage (**Figure S18**e). For sake of accuracy, the experiments were performed on an unmeasured OECT obtained by the same deposition as the OECT used for transfer characteristics (*i.e.* one spin-coating on one substrate provides two OECTs: one used for *C** evaluation via chronoamperometry and the other one for *μC** evaluation via transfer characteristics). This allows us to avoid sample-to-sample divergences, especially morphology variations, in order to more accurately determine μ_{OECT}. In chronoamperometry, the voltage sign is based on the electrochemistry convention where the quasi-reference Ag/AgCl 'gate' is grounded (*i.e.*, $V_{GS} > 0$ induces p-doping). The voltage was applied using a data acquisition system (USB-6211 from National Instrument). To increase time-resolution, the resulting current was converted into a voltage using a low-noise current preamplifier (SR570 from Stanford Research Systems, sensitivity 200 or 500 μA V^{-1}) and measured with a USB-6211 acquisition system, thereby affording a time-resolution of 8 μs.

Second, EIS measurements were conducted following a previously reported procedure.[36] Briefly, EIS were performed using a BioLogic SP-200 potentiostat in three-electrode mode, with an Ag/AgCl electrode serving as the pseudo-reference electrode, a Pt mesh as the counter electrode, **PBTTT-^{8}O** thin film on Au substrates as the working electrode, in an 0.1 M KPF_6 aqueous electrolyte. The voltage bias was set to -0.9 V vs. Ag/AgCl, and the frequency range was 1 Hz–100 kHz. A Randles circuit model Rs + Rp/CPE was used to fit the curves and to extract the capacitance. In this circuit, Rs is the resistance of active electrolyte, Rp represents the resistance in charge transfer, and CPE is a constant phase element. Both pristine and aligned **PBTTT-^{8}O** thin films were fabricated with a series of volumes and all measured the same way. However, no linear correlation between volume and capacitance is found which indicates that EIS is not suitable for an exact quantification of *C**. EIS measurement focuses on frequency-based AC impedance. The response frequency of **PBTTT-^{8}O** is too low and does not match the typical EIS frequency range. Under low-frequency testing, complex ion-diffusion impedance greatly interferes with testing. Nonetheless, for the sample with the smallest volume and at frequencies superior to 1 Hz, a value of *C** of 350 F cm^{-3}, in the same range than the *C** from chronoamperometry, was found for pristine **PBTTT-^{8}O** (**Figure S19**).

Note S6. Determination of μ_{hole}.

Four distinct methods were employed to determine the **hole mobility in the swollen state μ_{hole}**: (i) inferred from μC^* and C^*, (ii) inferred from the slope of $\sqrt{I_{DS}^{Sat}}$ vs. V_{GS} plot and C^* (independent of V_{Th} and V_{GS} extractions), and **two direct measurements** (*i.e.*, independent of C* extraction) (iii) using impedance matching, and (iv) using *in-situ* THz spectroscopy. The measurements are discussed below.

i. Inferred from μC^* and C^*

This method is commonly employed in literature, as shown by the many articles with the note [d] in **Table S1**. The drawback is that it relies on the assumptions of the Bernards-Malliaras model which likely leads to an **overestimation of mobility**. For the mm-long channel, a $\mu_{pristine}$ of 2.5 ± 0.3 cm^2 V^{-1} s^{-1} is found for pristine OECTs, and a **7.6× enhancement** is found for aligned OECTs with a $\mu_{aligned}$ of 19 ± 4.0 cm^2 V^{-1} s^{-1}.

ii. Inferred from the slope of $\sqrt{I_{DS}^{Sat}}$ vs. V_{GS} plot and C^*

This method exists but is rare in literature, as shown by the few articles with the note [e] in **Table S1**. This method also **overestimates mobility**. It is extracted from the $\sqrt{I_{DS}^{Sat}}$ vs V_{GS} plot (**Figure S13**) using the slope of the linear extrapolation, according to:

$$\sqrt{I_{DS}^{Sat}} = \sqrt{\frac{Wd}{2L}\mu C^*} \cdot (V_{Th} - V_{GS}) \Rightarrow \text{slope} = \sqrt{\frac{Wd}{2L}\mu C^*} \Leftrightarrow \mu C^* = \frac{2L}{Wd} \cdot \text{slope}^2 \quad \text{Equation (3)}$$

For the mm-long channel OECTs, a $\mu_{pristine}$ of 3.3 ± 0.8 cm^2 V^{-1} s^{-1} is found for pristine OECTs, and a **4.5× enhancement** is found for parallelly aligned OECTs with a $\mu_{aligned\_para}$ of 15 ± 9.8 cm^2 V^{-1} s^{-1}, averaged over 7-10 samples (**Figure S13**). For comparison, when then film is aligned but *perpendicular* to the channel direction, $\mu_{aligned}$ drops by a factor /67 to $\mu_{aligned\_perp}$ of 0.24 ± 0.03 cm^2 V^{-1} s^{-1} (**Figure S15**). For the 500-µm-long OECTs, a $\mu_{pristine}$ of 0.54 ± 0.06 cm^2 V^{-1} s^{-1} is found for pristine OECTs, and a **12× enhancement** is found for aligned OECTs with a $\mu_{aligned}$ of 6.5 ± 0.8 cm^2 V^{-1} s^{-1} (**Figure S17**). The lower values compared to the mm-long channel OECTs may be due to the film deterioration which occurred during the parylene-C lift-off (**Figure S5**). Loss of performance during shipping under ambient conditions cannot be excluded as well.

iii. Directly measured from impedance matching

This method is common in the literature to independently estimate μ, as shown by the many

articles with the note [f] in **Table S1**. In our case, this method likely **underestimates the mobility** given the low frequency range on which matching was achieved. For the 500-µm-long channel OECTs, a $\mu_{pristine}$ of 0.11 cm^2 V^{-1} s^{-1} is found for the pristine OECT, and a **17× enhancement** is found for aligned OECTs with a $\mu_{aligned}$ of 2.0 cm^2 V^{-1} s^{-1} (**Figure S24**).

The impedance matching measurements were conducted following a previously reported procedure.[46] Briefly, the measurement was conducted using a PXIe-4163 functional unit of a National Instruments (NI) PXI system, and controlled by a custom LabVIEW program. The gate-source I_{GS} current and the drain-source I_{DS} current were recorded with the NI PXI system. The relationship between I_{GS} and I_{DS} can be described as $\Delta I_{GS}(f) = 2\pi f\,\tau_{\mathrm{hole}}\,\Delta I_{DS}(f)$, where f is the frequency of the sinusoidal gate voltage, $\Delta I_{GS}(f)$ represents the change in I_{GS}, $\Delta I_{DS}(f)$ represents the change in I_{DS}, τ_{hole} is the hole transit time in the conducting polymer channel, which can be derived from the equation.[46] Thus, hole mobility μ_{hole} is calculated with the equation $\mu_{\mathrm{hole}} = L^2/(V_{DS}\times\tau_{\mathrm{hole}})$,[44] where L is the channel length.

iv. Directly measured from *in-situ* THz spectroscopy

Due to the technical difficulty, this method is rarely applied to OECTs and has only been reported once for pristine P3HT in the literature.[47] It allows to independently estimate μ, but conversely to impedance matching, *in-situ* electrochemical THz spectroscopy only probes the short-range μ of mobile charge carriers on the nanometer-scale. This method probes the charge transport within the THz electric field pulse, thus without impact of electrodes nor of the ionic mobility. The *in-situ* THz spectroscopy samples were coated on quartz substrates purchased from Technical Glass Products. The area of the polymer film was defined using a precision tip cotton swap to 5x5 mm, including an overlap of 1 mm on each electrode (*i.e.*, W = 5 mm and L = 3 mm). Kapton tape was put on the electrodes to minimize leakage current. A home-built electrochemical cell was used, with a similar Ag/AgCl electrode than for OECTs and the same KPF_6 electrolyte. The THz waveforms were acquired at a dedoping voltage of +0.3 V (reference) and doping voltages of -0.5 V and -0.8 V (signal), with SD short-circuited contacts to have uniform electrochemical doping across the channel. The voltages were applied for a dwell time of 150 s before recording the transmitted THz signal. For aligned samples, the alignment of the polymer was set parallel to the polarization of the THz beam to selectively probe the transport in the direction of chain alignment. The THz waveforms (**Figure S25**) show a decrease in amplitude and shift when electrochemically doping the films in both pristine and aligned **PBTTT-^{8}O** at increasing doping voltage. The decrease of the THz field is attributed to

the formation of conductive charges, which absorb part of the signal, while the phase shift is due to changes of the refractive index. For the aligned samples, the reduction of the signal is more prominent compared to the pristine ones, indicating a higher conductivity.

After applying Fourier transformation to these waveforms, the complex conductivity was obtained (**Figure S26**). The real part (solid lines) tells us about the conductivity of the charges when they are displaced over a short distance (a few nanometers) by the THz electric field in the short duration of the laser pulse. The imaginary part (dashed lines) informs about the delocalization of the charge carriers. For aligned **PBTTT-^{8}O**, a real conductivity of 1022 S cm^{-1} at 1 THz was obtained, being a **2.0× enhancement** compared to the real conductivity of 510 S cm^{-1} at 1 THz found for pristine samples. These results indicate that polymer alignment improves electrical transport even over a distance of a few nanometers.

To fit the complex conductivities, the phenomenological Drude-Smith model was applied. The fits are shown in **Figure S26** and the parameters are given in **Table S5**. The Drude-Smit model described the complex conductivity as follows: $\tilde{\sigma} = \frac{\varepsilon_0 \omega_P^2 \tau}{1-\mathrm{i}\omega\tau}\left[1 + \frac{c_1}{1-\mathrm{i}\omega\tau}\right]$, where ε_0 is the free space permitivity, ω_P is the plasma frequency, τ the scattering time and c_1 the localization parameter, which gives a value between 0 and -1. Whereas the Drude model applies if c_1 equals 0, showing the presence of free charges, the closer the value is to -1 the stronger localized the charges are. The scattering time τ was set to 24 fs and 28 fs for pristine and aligned films respectively, which is usual for conjugated polymers. The conductive charge density $N_{Drude\text{-}Smith}$ (typically abbreviated N_{DS}) can be extracted from the Drude-Smith model by using the following equation: $\omega_P = \sqrt{\frac{N_{DS}\, e^2}{\varepsilon_0 m^*}}$. A comparable charge density N_{DS} of 7-8 x 10^{20} cm^{-3} is found for both samples, so that the difference in conductivity stems mainly from a higher carrier mobility for aligned **PBTTT-^{8}O**. In the next step, the effective "short-range" THz mobility, over the nanometer-length scale, can be calculated from the parameters obtained with the Drude-Smith model, following: $\mu_{eff} = \frac{e\tau}{m^*}(1 + c_1)$. Assuming an effective mass m^* of 1.7 m_e (as for P3HT[48]), an effective short-range mobility $\mu_{eff\_pristine}$ of 3.7 cm^2 V^{-1} s^{-1} is found for the pristine OECT, and a **1.8× enhancement** is found for aligned OECTs with a $\mu_{eff\_aligned}$ of 6.5 cm^2 V^{-1} s^{-1}. This is in agreement with the trend seen with the σ_{THz} conductivity measured at 1 THz. The main reason for the higher mobility in the aligned film is the less negative value of c_1, indicating a higher charge delocalization due to better nanoscale order.

Note S7. Determination of doping, dedoping, ON and OFF time constants

All time-resolved pulsing stability coupled with Vis-NIR spectroelectrochemistry experiments were performed in degassed 0.1 M KPF_6/H_2O electrolyte *but without inert gas blanket*. All OECTs were cycles ON and OFF 1000 times each by applying square-wave V_{GS} and V_{DS} pulses with continuous recording of the resulting I_{DS} current and Vis-NIR absorbance spectra using a LabVIEW program. V_{GS} was applied using a USB-6211 acquisition card (National Instrument Corp.), while a Keithley 2400 SMU (Tektronix) is used to apply V_{DS} and record I_{DS}. The Vis-NIR absorbance spectra were acquired with a Flame UV-Vis and a FlameNIR spectrometers (Ocean Optics). In the ON state, V_{GS} = -0.8 V and V_{DS} = -0.5 V were applied to operate in the saturation regime and held for 3 seconds. In the OFF state, V_{GS} = +0.5 V and V_{DS} = 0 V were applied and held for 1 second. The total stress duration was hence of 4000 seconds, *i.e.* 1 h 6 min.

The temporal evolution of both the **absorbance at 540 nm** (~neutral band, wavelength sampling) and resulting **I_{DS} current** are fitted with the sum of a mono-exponential with amplitude A and a constant (k) similarly to as done by *Guo et al.*[49] and described below.:

(i) The doping time constant (τ_{doping}) and the doping front propagation time (t_{p_doping}) are extracted from the decay of the absorbance normalized by the maximum absorbance at 540 nm for all cycles, using:

$$Abs(t) = A \cdot e^{-\frac{(t-t_{p\_doping})}{\tau_{doping}}} + k \qquad \text{Equation (4)}$$

(ii) The dedoping time constant ($\tau_{dedoping}$) is extracted from the rise of the absorbance normalized by the maximum absorbance at 540 nm for all cycles, using:

$$Abs(t) = -A \cdot e^{-\frac{t}{\tau_{dedoping}}} + k \qquad \text{Equation (5)}$$

(iii) The ON time constant (τ_{ON}) and the I_{DS} ON "propagation time" (t_{p_ON}) are extracted from the decay of the I_{DS} current normalized by abs($I_{DS,max}$), the absolute value of the maximum I_{DS} reached for all cycles, using:

$$I_{DS}(t) = A \cdot e^{-\frac{(t-t_{p\_ON})}{\tau_{ON}}} + k \qquad \text{Equation (6)}$$

(iv) The OFF time constant (τ_{OFF}) is extracted from the rise of the I_{DS} current normalized by abs($I_{DS,max}$), the absolute value of the maximum I_{DS} reached for all cycles, using:

$$I_{DS}(t) = -A \cdot e^{-\frac{t}{\tau_{OFF}}} + k \qquad \text{Equation (7)}$$

Absorbance decay and I_{DS} decay are fitted after 5% loss of the maximum value to exclude the initial propagation time. The resulting curves and fit are presented in **Figure S27**. The fit quality is evaluated by the cycle-after-cycle evolution of R^2 shown in **Figure S28**.

Note S8. Cyclic voltammetry

The cyclic voltammetry (CV) is performed with a conventional 3-electrode system using a VSP-300 Potentiostat (BioLogic). PBTTT-^{8}O is drop-casted from 1 mg/mL chloroform solution at RT on a platinum micro disk (2 mm diameter) working electrode. A platinum wire is used as counter electrode. A mercury sulfate electrode (MSE) with saturated K2SO4 solution is used as reference electrode (+0.64 V vs SHE; RE-2CP BAS Inc.) . The 0.1 M KPF_6/H_2O electrolyte is degassed before running the experiments by argon-bubbling. An argon blanket is kept during the whole duration of the experiment. The polymer film is pre-cycled 3 times to ensure fair comparison with the pulsing stability tests. The CV is then run at a scan rate of 100 mV s^{-1} over 1000 cycles (about 6 h 40 min).

References

[1] S. E. Chen, L. Q. Flagg, J. W. Onorato, L. J. Richter, J. Guo, C. K. Luscombe, D. S. Ginger, *J. Mater. Chem. A* **2022**, 10, 10738.

[2] P. Durand, H. Zeng, T. Biskup, V. Vijayakumar, V. Untilova, C. Kiefer, B. Heinrich, L. Herrmann, M. Brinkmann, N. Leclerc, *Adv. Energy Mater.* **2022**, 12, 2103049.

[3] D. A. Bernards, G. G. Malliaras, *Adv. Funct. Mater.* **2007**, 17, 3538.

[4] M. Shahi, V. N. Le, P. Alarcon Espejo, M. Alsufyani, C. J. Kousseff, I. McCulloch, A. F. Paterson, *Nat. Mater.* **2024**, 23, 2.

[5] M. Cucchi, A. Weissbach, L. M. Bongartz, R. Kantelberg, H. Tseng, H. Kleemann, K. Leo, *Nat. Commun.* **2022**, 13, 4514.

[6] S. T. Keene, L. W. Gatecliff, S. L. Bidinger, M. Moser, I. McCulloch, G. G. Malliaras, *MRS Commun.* **2023**, 1.

[7] S. Griggs, A. Marks, D. Meli, G. Rebetez, O. Bardagot, B. D. Paulsen, H. Chen, K. Weaver, M. I. Nugraha, E. A. Schafer, J. Tropp, C. M. Aitchison, T. D. Anthopoulos, N. Banerji, J. Rivnay, I. McCulloch, *Nature Communications* **2022**, 13, 7964.

[8] C. Cendra, A. Giovannitti, A. Savva, V. Venkatraman, I. McCulloch, A. Salleo, S. Inal, J. Rivnay, *Adv. Funct. Mater.* **2019**, 29, 1807034.

[9] M. Moser, Y. Wang, T. C. Hidalgo, H. Liao, Y. Yu, J. Chen, J. Duan, F. Moruzzi, S. Griggs, A. Marks, N. Gasparini, A. Wadsworth, S. Inal, I. McCulloch, W. Yue, *Mater. Horiz.* **2022**, 9, 973.

[10] A. Giovannitti, D.-T. Sbircea, S. Inal, C. B. Nielsen, E. Bandiello, D. A. Hanifi, M. Sessolo, G. G. Malliaras, I. McCulloch, J. Rivnay, *Proc. Natl. Acad. Sci.* **2016**, 113, 12017.

[11] A. Savva, C. Cendra, A. Giugni, B. Torre, J. Surgailis, D. Ohayon, A. Giovannitti, I. McCulloch, E. Di Fabrizio, A. Salleo, J. Rivnay, S. Inal, *Chem. Mater.* **2019**, 31, 927.

[12] A. Makhinia, L. Bynens, A. Goossens, J. Deckers, L. Lutsen, K. Vandewal, W. Maes, V. Beni, P. Andersson Ersman, *Adv. Funct. Mater.* **2024**, 34, 2314857.

[13] R. K. Hallani, B. D. Paulsen, A. J. Petty, R. Sheelamanthula, M. Moser, K. J. Thorley, W. Sohn, R. B. Rashid, A. Savva, S. Moro, J. P. Parker, O. Drury, M. Alsufyani, M. Neophytou, J. Kosco, S. Inal, G. Costantini, J. Rivnay, I. McCulloch, *J. Am. Chem. Soc.* **2021**, 143, 11007.

[14] O. Bardagot, B. T. DiTullio, A. L. Jones, J. Speregen, J. R. Reynolds, N. Banerji, *Adv. Funct. Mater.* **2024**, n/a, 2412554.

[15] L. Q. Flagg, W. Cho, J. Woodcock, R. Li, H. W. Ro, D. M. Delongchamp, L. J. Richter, *Chem. Mater.* **2024**, 36, 1352.
[16] L. Q. Flagg, C. G. Bischak, J. W. Onorato, R. B. Rashid, C. K. Luscombe, D. S. Ginger, *J. Am. Chem. Soc.* **2019**, 141, 4345.
[17] L. Huang, Z. Wang, J. Chen, B. Wang, Y. Chen, W. Huang, L. Chi, T. J. Marks, A. Facchetti, *Adv. Mater.* **2021**, 33, 2007041.
[18] T. C. Hidalgo Castillo, M. Moser, C. Cendra, P. D. Nayak, A. Salleo, I. McCulloch, S. Inal, *Chem. Mater.* **2022**, 34, 6723.
[19] M. Moser, T. C. Hidalgo, J. Surgailis, J. Gladisch, S. Ghosh, R. Sheelamanthula, Q. Thiburce, A. Giovannitti, A. Salleo, N. Gasparini, A. Wadsworth, I. Zozoulenko, M. Berggren, E. Stavrinidou, S. Inal, I. McCulloch, *Adv. Mater.* **2020**, 32, 2002748.
[20] P. O. Osazuwa, C.-Y. Lo, X. Feng, A. Nolin, C. Dhong, L. V. Kayser, *ACS Appl. Mater. Interfaces* **2023**, 15, 54711.
[21] J. Rivnay, S. Inal, B. A. Collins, M. Sessolo, E. Stavrinidou, X. Strakosas, C. Tassone, D. M. Delongchamp, G. G. Malliaras, *Nat. Commun.* **2016**, 7, 11287.
[22] S. Inal, G. G. Malliaras, J. Rivnay, *Nat. Commun.* **2017**, 8.
[23] M. Moser, A. Savva, K. Thorley, B. D. Paulsen, T. C. Hidalgo, D. Ohayon, H. Chen, A. Giovannitti, A. Marks, N. Gasparini, A. Wadsworth, J. Rivnay, S. Inal, I. McCulloch, *Angew. Chem. Int. Ed.* **2021**, 60, 7777.
[24] Y. Dai, S. Dai, N. Li, Y. Li, M. Moser, J. Strzalka, A. Prominski, Y. Liu, Q. Zhang, S. Li, H. Hu, W. Liu, S. Chatterji, P. Cheng, B. Tian, I. McCulloch, J. Xu, S. Wang, *Adv. Mater.* **2022**, 34, 2201178.
[25] M. Moser, L. R. Savagian, A. Savva, M. Matta, J. F. Ponder, Jr., T. C. Hidalgo, D. Ohayon, R. Hallani, M. Reisjalali, A. Troisi, A. Wadsworth, J. R. Reynolds, S. Inal, I. McCulloch, *Chem. Mater.* **2020**, 32, 6618.
[26] C. B. Nielsen, A. Giovannitti, D.-T. Sbircea, E. Bandiello, M. R. Niazi, D. A. Hanifi, M. Sessolo, A. Amassian, G. G. Malliaras, J. Rivnay, I. McCulloch, *J. Am. Chem. Soc.* **2016**, 138, 10252.
[27] X. Luo, H. Shen, K. Perera, D. T. Tran, B. W. Boudouris, J. Mei, *ACS Macro Letters* **2021**, 10, 1061.
[28] B. T. Ditullio, L. R. Savagian, O. Bardagot, M. De Keersmaecker, A. M. Österholm, N. Banerji, J. R. Reynolds, *J. Am. Chem. Soc.* **2023**, 145, 122.
[29] H. Jia, Z. Huang, P. Li, S. Zhang, Y. Wang, J.-Y. Wang, X. Gu, T. Lei, *J. Mater. Chem. C* **2021**, 9, 4927.
[30] G. Krauss, F. Meichsner, A. Hochgesang, J. Mohanraj, S. Salehi, P. Schmode, M. Thelakkat, *Adv. Funct. Mater.* **2021**, 31, 2010048.
[31] C. G. Bischak, L. Q. Flagg, K. Yan, T. Rehman, D. W. Davies, R. J. Quezada, J. W. Onorato, C. K. Luscombe, Y. Diao, C.-Z. Li, D. S. Ginger, *J. Am. Chem. Soc.* **2020**, 142, 7434.
[32] P. Schmode, A. Savva, R. Kahl, D. Ohayon, F. Meichsner, O. Dolynchuk, T. Thurn-Albrecht, S. Inal, M. Thelakkat, *ACS Appl. Mater. Interfaces* **2020**, 12, 13029.
[33] A. Giovannitti, R. B. Rashid, Q. Thiburce, B. D. Paulsen, C. Cendra, K. Thorley, D. Moia, J. T. Mefford, D. Hanifi, D. Weiyuan, M. Moser, A. Salleo, J. Nelson, I. McCulloch, J. Rivnay, *Adv. Mater.* **2020**, 32, 1908047.
[34] P. Schmode, D. Ohayon, P. M. Reichstein, A. Savva, S. Inal, M. Thelakkat, *Chem. Mater.* **2019**, 31, 5286.
[35] S. Inal, J. Rivnay, P. Leleux, M. Ferro, M. Ramuz, J. C. Brendel, M. M. Schmidt, M. Thelakkat, G. G. Malliaras, *Adv. Mater.* **2014**, 26, 7450.
[36] H.-Y. Wu, J.-D. Huang, S. Y. Jeong, T. Liu, Z. Wu, T. Van Der Pol, Q. Wang, M.-A. Stoeckel, Q. Li, M. Fahlman, D. Tu, H. Y. Woo, C.-Y. Yang, S. Fabiano, *Mater. Horiz.* **2023**, 10, 4213.

[37] M. K. Singh, A. Kumar, R. Prakash, *Org. Electron.* **2017**, 50, 138.
[38] V. Untilova, J. Hynynen, A. I. Hofmann, D. Scheunemann, Y. Zhang, S. Barlow, M. Kemerink, S. R. Marder, L. Biniek, C. Müller, M. Brinkmann, *Macromolecules* **2020**, 53, 6314.
[39] J. Rivnay, P. Leleux, M. Ferro, M. Sessolo, A. Williamson, D. A. Koutsouras, D. Khodagholy, M. Ramuz, X. Strakosas, R. M. Owens, C. Benar, J.-M. Badier, C. Bernard, G. G. Malliaras, *Sci. Adv.* **2015**, 1, e1400251.
[40] X. Tian, D. Liu, J. Bai, K. S. Chan, L. C. Ip, P. K. L. Chan, S. Zhang, *Anal. Chem.* **2022**, 94, 6156.
[41] S. Kim, H. Yoo, J. Choi, *Sensors* **2023**, 23, 2265.
[42] L. Dobrescu, M. Petrov, D. Dobrescu, C. Ravariu, 2000.
[43] T. Nunes Domschke, O. Bardagot, A. Benayad, R. Demadrille, A. Carella, R. Clerc, A. Pereira, *Synth. Met.* **2020**, 260, 116251.
[44] D. Ohayon, V. Druet, S. Inal, *Chem. Soc. Rev.* **2023**, 52, 1001.
[45] D. L. J. Alexander, A. Tropsha, D. A. Winkler, *Journal of Chemical Information and Modeling* **2015**, 55, 1316.
[46] J. Rivnay, M. Ramuz, P. Leleux, A. Hama, M. Huerta, R. M. Owens, *Applied Physics Letters* **2015**, 106, 043301.
[47] D. Tsokkou, P. Cavassin, G. Rebetez, N. Banerji, *Mater. Horiz.* **2022**, 9, 482.
[48] J. E. Northrup, *Phys. Rev. B* **2007**, 76, 245202.
[49] J. Guo, S. E. Chen, R. Giridharagopal, C. G. Bischak, J. W. Onorato, K. Yan, Z. Shen, C.-Z. Li, C. K. Luscombe, D. S. Ginger, *Nat. Mater.* **2024**, 23, 656.
